# Proteome Analysis of *Borrelia burgdorferi* Response to Environmental Change

**DOI:** 10.1371/journal.pone.0013800

**Published:** 2010-11-02

**Authors:** Thomas E. Angel, Benjamin J. Luft, Xiaohua Yang, Carrie D. Nicora, David G. Camp, Jon M. Jacobs, Richard D. Smith

**Affiliations:** 1 Biological Sciences Division, Pacific Northwest National Laboratory, Richland, Washington, United States of America; 2 Division of Infectious Diseases, School of Medicine, State University of Stony Brook, Stony Brook, New York, United States of America; National Institutes of Health, United States of America

## Abstract

We examined global changes in protein expression in the B31 strain of *Borrelia burgdorferi,* in response to two environmental cues (pH and temperature) chosen for their reported similarity to those encountered at different stages of the organism's life cycle. Multidimensional nano-liquid chromatographic separations coupled with tandem mass spectrometry were used to examine the array of proteins (i.e., the proteome) of *B. burgdorferi* for different pH and temperature culture conditions. Changes in pH and temperature elicited *in vitro* adaptations of this spirochete known to cause Lyme disease and led to alterations in protein expression that are associated with increased microbial pathogenesis. We identified 1,031 proteins that represent 59% of the annotated genome of *B. burgdorferi* and elucidated a core proteome of 414 proteins that were present in all environmental conditions investigated. Observed changes in protein abundances indicated varied replicon usage, as well as proteome functional distributions between the *in vitro* cell culture conditions. Surprisingly, the pH and temperature conditions that mimicked *B. burgdorferi* residing in the gut of a fed tick showed a marked reduction in protein diversity. Additionally, the results provide us with leading candidates for exploring how *B. burgdorferi* adapts to and is able to survive in a wide variety of environmental conditions and lay a foundation for planned *in situ* studies of *B. burgdorferi* isolated from the tick midgut and infected animals.

## Introduction

*B. burgdorferi*, the causative agent of Lyme disease, has a complex life cycle in which the microbe resides in widely divergent environments that include the midgut of the *Ixodes* tick, tick salivary glands, and in its pathogenic state within the mammalian reservoir when passed to the inadvertent human host [1]. The principle environmental conditions of pH and temperature constitute important signals leading to alterations in gene transcription [2], [3], [4], [5], [6], [7], [8], [9], [10], [11] and protein expression [4], [12], [13], [14], [15] thought to facilitate the bacterial transmission process from tick to mammalian host. For example, the temperature and pH environment of the *Ixodes* tick midgut is altered following a blood meal [3]. In addition to the principle environmental conditions of temperature and pH, a multitude of host related factors, for example those derived from blood from tick feeding, have been demonstrated to have effects on gene transcription and protein expression *in vitro* and likely play an important role *in vivo* [12], [16]. *In vitro* cell culture has been used to examine differential gene expression of *B. burgdorferi* in response to altering pH and temperature; however the broad changes in protein abundances under different pH and temperature conditions have not been previously elucidated.

Observed changes in protein abundance of *B. burgdorferi* grown in batch cultures representing established minimal models of bacterial growth in the unfed tick vector (23°C, pH 7.6) and the fed tick vector (34°C, pH 6.6) provide insights into global adaptation of the microbe resulting from changes in two important environmental conditions [7]. Although gel-based proteomics strategies have been employed to investigate the dynamics of the *B. burgdorferi* proteome [4], [5], [17], these pioneering studies identified only a limited number of proteins. As a result, insights into the global changes in protein expression based on changing environmental conditions were limited. The present study explores the effect of changing principle environmental conditions where there is a wealth of previously published orthogonal transcriptomic data [6], [7], [16], [18] allowing for comparative analyses.

Here, we present a non-gel, mass spectrometry-based study of the global changes in the proteome of the well-characterized infectious B31 strain of *B. burgdorferi* in response to culture conditions analogous in part to what the organism experiences during its life cycle, e.g., growth in the unfed or fed tick vectors. Previous mass spectrometry (MS)-based proteomics studies provided a foundation for the current effort in that Jacobs et al, described the detectable protein distribution for three different strains of *Borrelia* all grown in similar culture conditions that allowed for strain-to-strain comparisons [15]. New advances in MS technologies afford even greater depth of coverage than reported in the earlier study.

Armed with knowledge of the complete *B. burgdorferi* B31 genome [19], we applied multi-dimensional separations coupled with tandem mass spectrometry (MS/MS) to characterize the proteome of *B. burgdorferi*. We measured changes in proteins expressed by *B. burgdorferi* grown under different *in vitro* batch culture conditions and correlated the response between environmental factors and observed protein patterns. By combining knowledge of the proteome adaptation triggered by variation of principle environmental signals with existing transcriptome data, we obtained a more complete understanding of the changes occurring within *B. burgdorferi* as it responds to these environmental cues. This information provides us with important insight into potential candidate proteins which are changed as the organism prepares to infect the next host.

## Results

### The B. burgdorferi proteome

We analyzed the proteome of *B. burgdorferi* grown in a total of five different environmental conditions (i.e. varied pH and temperature) that included growth at mid-log phase at reduced temperature and pH 7.6 (RT; 23°C, pH 7.6) and at 34°C and reduced pH (RpH; 34°C, pH 6.6). Results were compared against control protein profiles in log culture (Log; 34°C, pH 7.6), high cell density (HD; 34°C, pH 7.6) and a low passage-high cell density culture (LP; 34°C, pH 7.6). Following strong cation exchange (SCX) fractionation of tryptic peptides (originating from proteins isolated from the 5 different growth conditions) and high resolution, reversed phase liquid chromatography LC-MS/MS analysis, 18798 unique peptides were identified with high confidence. These peptides represented 1031 proteins, with 837 proteins covered by multiple unique peptides.

**Figure 1:**
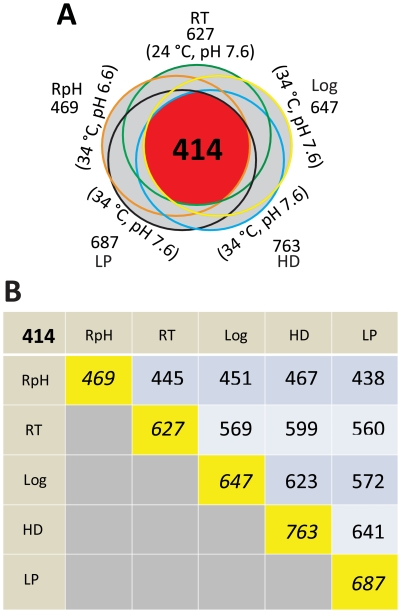
Overview and comparison of identified proteins for all culture conditions. A) Venn diagram depicting the number of unique *B. burgdorferi* proteins identified in each of the five culture conditions: RpH (reduced pH), RT (reduced temperature), Log, LP (low passage), and HD (high cell density). The 414 proteins that support growth across all culture conditions studied represent a core proteome for this organism. B) A pair wise comparison that depicts the number of proteins identified in common between two conditions, as well as the total number of proteins identified for each culture condition and across all culture conditions (i.e., the core proteome).

A comparison of the proteins detected in each of the growth conditions revealed 414 proteins common to all conditions (Figure 1A and B), which represents ∼24% of the theoretical annotated proteome. This subset of 414 proteins represents the core of detectable proteins that support growth across all culture conditions studied, i.e., a “core proteome”. A summary of detected proteins for each individual culture condition is presented in Figure 1B. (A detailed list is provided in Table S1). Note the marked reduction in the number of proteins identified in the RpH culture relative to all other environmental conditions. This observation was striking as the comparative analysis approach utilized identical initial peptide amounts for each LC-MS/MS analysis (see Figure S1 for representative chromatograms). Thus we are observing a reduction in protein diversity, not simply a reduction in protein abundance.

### Analysis of the functional distribution of the proteome

**Figure 2:**
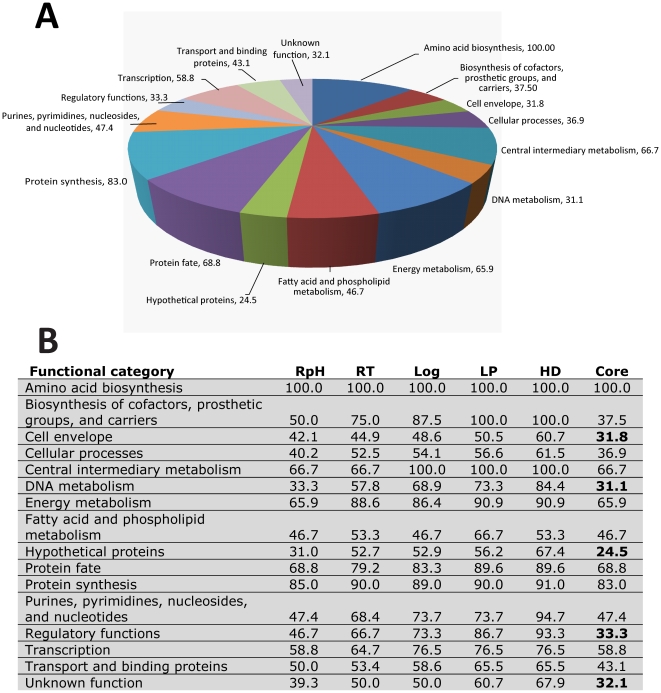
Analysis of the core proteome of *B. burgdorferi*. A) Functional distribution of the 414 proteins that represent the core proteome of *B. burgdorferi*. The percentage of possible proteins identified per functional category for the core proteome is shown. B) Comparison of the identified functional distribution of proteins across all culture conditions. A low percentage value in the core proteome reflects poor conservation or heterogeneity of these proteins across growth conditions.

The core proteome of *B. burgdorferi* in this study consists of 414 proteins detected in all cell culture conditions. The functional distribution of these “core” proteins is represented in Figure 2A. Proteins identified outside of the core proteome in the various culture conditions reflect proteome diversity and the necessary biological adaptation for growth in that condition. Comparing percentages of each functional category identified for each culture condition reveals the RpH culture has a reduced distribution of proteins across all functional categories (Figure 2B). The five categories under the core with the lowest percentages (highlighted in bold in Figure 2B) are cell envelope, DNA metabolism, hypothetical proteins, regulatory functions, and unknown functions. These categories represent those in which the highest diversity across all conditions is observed, i.e., the lowest amount of proteome overlap between the conditions. Increased diversity in cell envelope proteins across culture conditions reflects the well established paradigm of reciprocal expression of outer surface proteins such as OspC and OspA and highlights the importance of these findings given that changing protein expression patterns represent changing targets for clinical detection and therapeutic intervention as well as treatment development [20].

**Figure 3:**
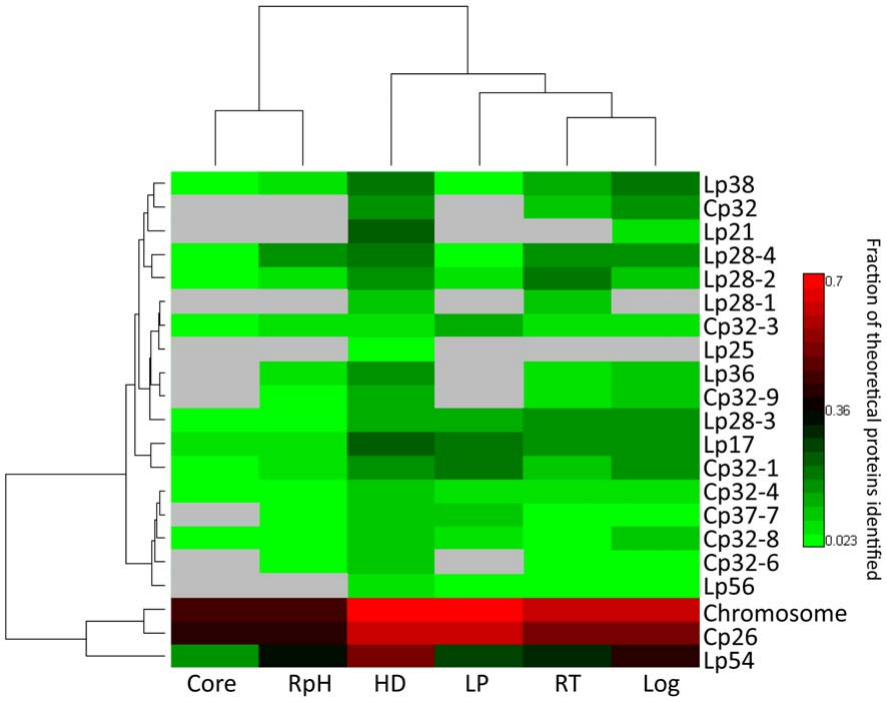
Hierarchical cluster analysis of replicon usage for each cell culture growth condition that depicts the fraction of the total possible proteins detected per replicon for each *in vitro* culture condition, as well as that of the core proteome. This comparative analysis shows that the RpH culture of *B. burgdorferi* exhibits a distinct pattern of replicon usage relative to the other culture conditions. This finding likely reflects different metabolic behavior and is descriptive of the cell culture phenotype in the RpH culture environment.

Proteins for each culture condition were also categorized by gene location. An unsupervised hierarchical cluster analysis was performed on the percentage of total proteins detected in each culture condition for each replicon (Figure 3). The genome of *B. burgdorferi* strain B31 is comprised of a single chromosome, 9 circular plasmids, and 12 linear plasmids. We detected proteins encoded on all plasmids with the exception of lp5. Comparing the distribution of proteins detected in *B. burgdorferi* across all conditions as a function of genetic element origin shows discriminate profiles between RpH and RT cultures (as shown in Figure 3). Observations include a notable increase in the number of proteins expressed on lp54 for RpH compared with RT, consistent with results from transcriptomic studies [7], [16], [18] and contrasting the overall observed reduction in expressed proteins for RpH. Additionally, we observed a lack of expressed proteins from lp56, lp28-1, and cp32 in RpH (34°C, pH 6.6) compared to RT (23°C, pH 7.6) cultures. Overall, the combined changes in pH and temperature appear to have a compound effect that potentially drives protein expression and genetic element usage in a unique manner for the bacteria growing in the RpH environment.

### Alterations in protein abundance in response to changes in temperature and pH

**Figure 4:**
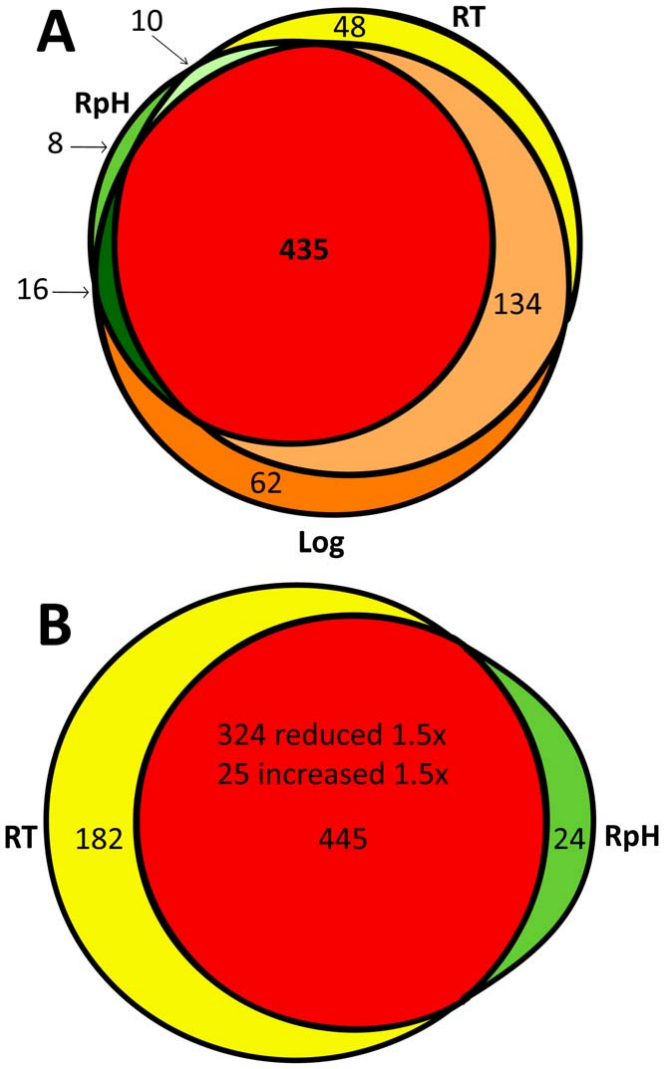
Comparison of proteins identified in three cultures at log phase. 435 proteins are in common to the three cultures, 8 are unique to the RpH culture (see Table S2), 48 are unique to the RT culture, and 62 are unique to the Log culture A). Subtractive analysis indicates 16 proteins are present because of increasing pH, and 134 are identified in cultures grown at elevated temperature. B) A comparison of proteins identified in the cultures that mimic growth in the RpH (reduced pH) and the RT (reduced temperature). 182 proteins are unique to the reduced temperature culture and 24 are unique to the reduced pH culture.

Because many environmental factors, including pH and temperature [3], [4], [5], [6], [7], orchestrate *B. burgdorferi* gene expression, we identified and quantified changes in relative protein abundance as a function of environmental conditions. The number of unique protein identifications in RT, RpH, and Log *in vitro* cultures grown to log stage are compared in Figure 4A, with 435 proteins found in common (see Table S2). Note the minimal overlap of protein identifications for the RpH culture with RT and Log cultures (48 and 62 unique proteins, respectively), a clear indication that the combined effect of increased temperature and lower pH reduce the total proteome diversity. We identified 66 proteins that were reported in previous transcriptomics studies to increase in transcript levels as a result of elevated cell culture temperature [6] (Table S3). Of these 66 proteins, 24 increased in abundance in the Log culture compared to the RT culture. In total, 77 proteins exhibited increases in abundance in Log compared to RT. Comparison of proteins identified in RpH and RT cultures (Figure 4B) reveals 445 proteins common to both conditions, 182 proteins unique to the RT culture, and 24 proteins unique to the RpH culture, all of which were identified with high confidence (covered by ≥2 unique peptides; see Table S4).

**Figure 5:**
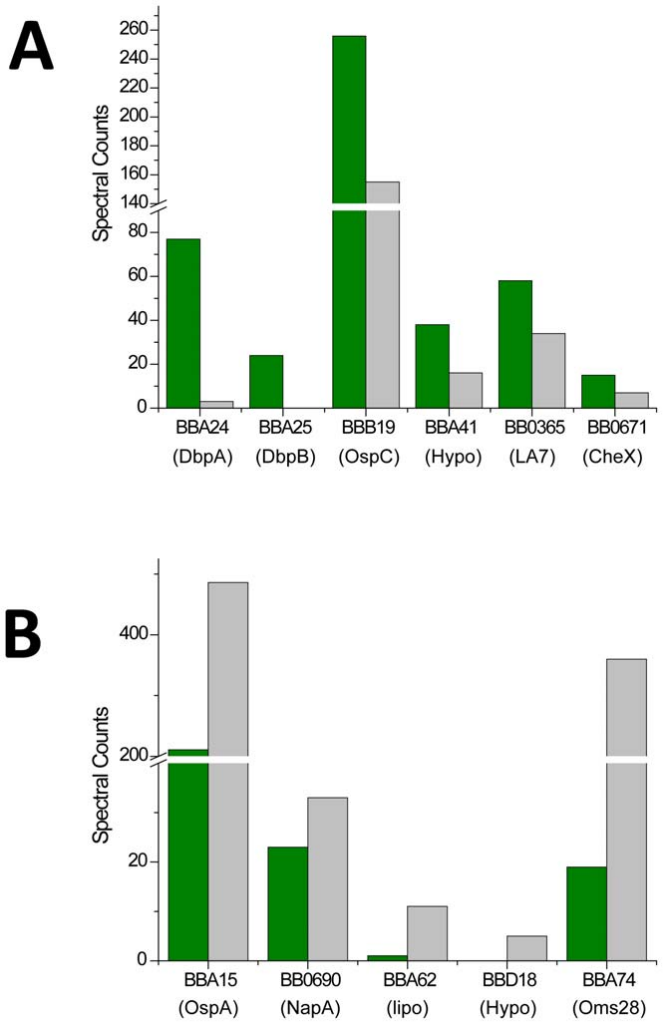
Semi-quantitative analysis of protein abundance by spectral counting. A) Representative protein spectral count values show an increased abundance for proteins isolated from *B. burgdorferi* grown in RpH (green bars) vs. RT (grey bars) culture conditions and B) representative proteins with increased spectral counts for RT culture conditions.

Quantification by spectral counting revealed changes in protein abundances in *B. burgdorferi* consistent with current models for *Borrelia* adaptation to changing growth conditions [21]. Figure 5 depicts observed increases and decreases in protein abundances for RpH and RT cultures. Changes in RpH are consistent with previously reported changes in *B. burgdorferi* following a tick taking a blood meal. For example, both decorin binding proteins (BBA24 and BBA25) and OspC (BBB19) clearly show increased abundance in the RpH culture. Interestingly, 17 ribosomal proteins in both cultures exhibited large differences in abundance, with changes ranging from 1.5 to 9 fold. Additionally, mediators of cell division FtsH (BB0789) and FtsZ (BB0229) were observed at reduced levels in the RpH culture.

**Figure 6:**
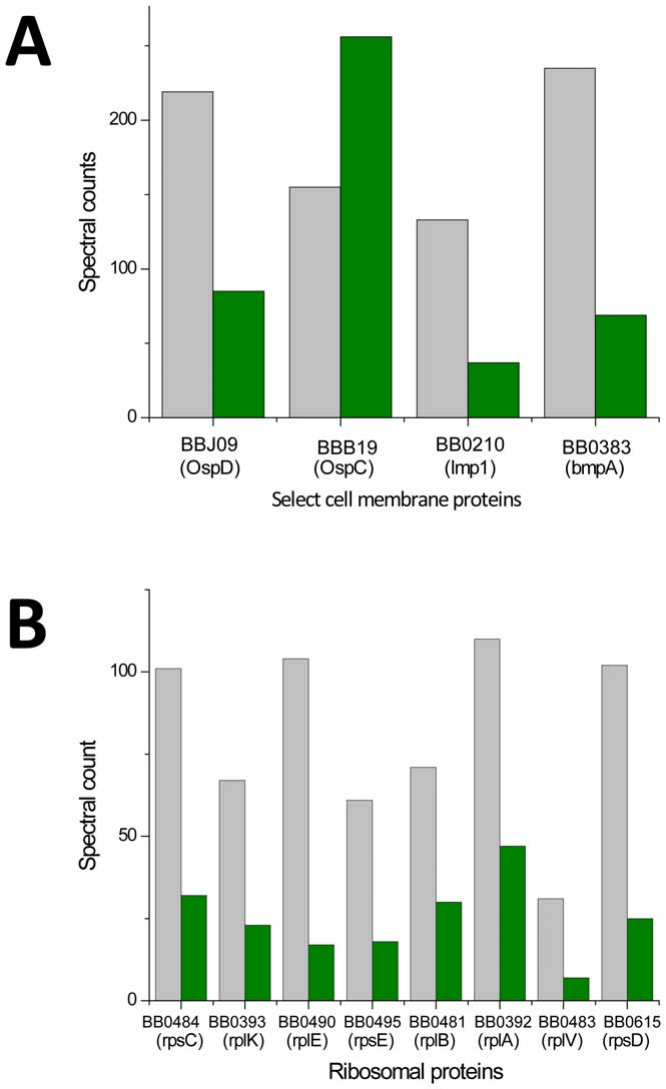
Changes in protein abundance inferred from spectral counts of peptides from proteins harvested from *in vitro* models of *B. burgdorferi* growth in RpH (green bars) vs. RT, (grey bars). Statistical significance was determined by ANOVA with p values ≤0.05. Changes in protein abundance for lipoproteins are consistent with previous observations, which led to recognition of reciprocal expression patterns for the outer surface proteins exemplified by Osp C (A). The statistically significant and considerable reduction in abundance of ribosomal proteins (B) is consistent with the net reduction in protein abundance observed for the *B. burgdorferi* in the RpH culture.

For further comparative analysis and verification, reprocessing and triplicate analyses of technical replicates of *B. burgdorferi* samples from the five different culture conditions were performed without SCX fractionation. Results were consistent with and verified our in-depth SCX fractionated sample dataset. Importantly, these results allowed a more rigorous statistical view of differential protein abundances and uncovered a metabolic shift in the *B. burgdorferi* grown in the RpH culture. Along with the expected changes in abundance of cell envelope proteins, we observed significant changes in abundance of ribosomal proteins, mediators of transcription, energy, and metabolism that were determined by analysis of variance (ANOVA) with p value ≤0.05 (Figure 6). An expected change observed was the increased abundance of OspC in the RpH culture, representing an important virulence factor given that it can bind tick salivary protein Salp15 (a protein that inhibits the activation of CD4^+^ T cells) that modulates host responses during bacterial infection [22].

## Discussion

### Membrane protein abundance changes in response to paralog families and gene location

Numerous alterations in the cell membrane protein profile of *B. burgdorferi* are reflected in the RpH compared to the RT conditions. Interestingly, the 39 up-regulated proteins in the RpH culture appear disproportionally distributed across the genome; 14 proteins originate from the chromosome, 15 lie on linear plasmid lp54, and the other 10 are scattered across seven other plasmids. Cell membrane proteins represent the largest fraction of this distinctive group, with 14 out of 39 annotated as cellular envelope (see Table S5). Additionally, paralog family 54 has the greatest presence in these proteins as well, with BBA64, BBA66, and BBA73 as cell envelope proteins. These results are consistent with previous reports which indicate that many members of paralog family 54 are affected by changing culture conditions [6], [7], [18]. In fact it has been recently reported that BBA64 plays an important role in bacterial transmission from tick to mammalian host [23].

In the *B. burgdorferi* genome paralog gene families are thought to have developed because of chromosome and plasmid duplication. Of the 161 paralogous gene families, a majority (107) are plasmid borne. Overall, observations showed an increase in the number of proteins identified between the RpH and RT cultures for paralog families 54, 33, 37, 44, and 74. Notably, paralog family 54, composed of 14 members of which 11 are annotated as lipoproteins, exhibited the greatest change in family member content between RpH and RT culture conditions. Given that these proteins appear distinctive for the RpH culture of *B. burgdorferi*, they represent new potential follow-on targets for further characterization and possible development of diagnostic tools and clinical intervention. Interestingly the culture conditions investigated here are sufficient for induction of expression of OspD at reduced temperatures, increasing the culture temperature and reducing pH leads to an observed reduction in OspD abundance (Figure 6) consistent with previously published results [6], [16], [24].

### Protein expression controlled by sigma factors

**Figure 7:**
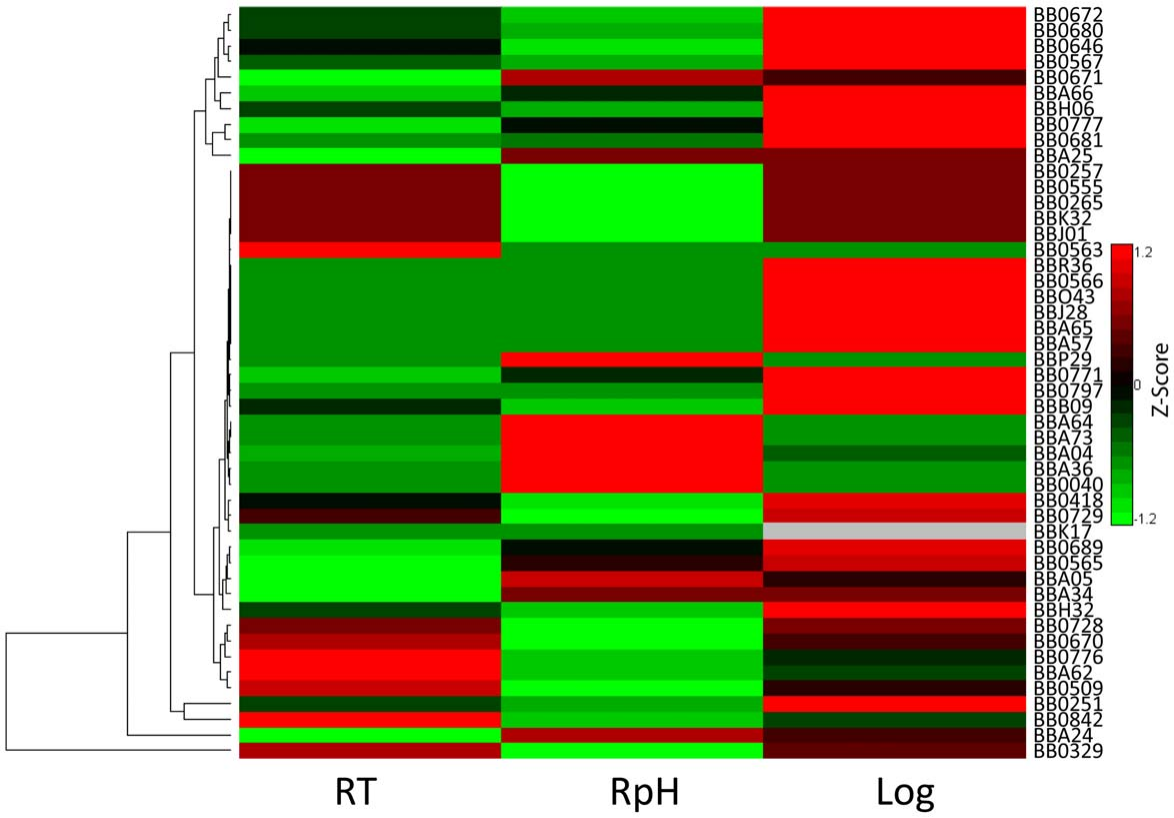
Hierarchical cluster analysis of protein abundances for proteins previously reported to be transcriptionally regulated by alternative sigma factors Rrp2, RpoN, RpoS. Protein abundance values were inferred from spectral counts of associated peptides. This analysis shows a discrete protein abundance pattern for each culture condition and demonstrates that adaptation to changing temperature and pH is regulated in part by the alternative sigma factors.

Prokaryotic transcription is controlled by several different sigma factors [25]. Changing environmental conditions lead to differential activation and usage of these sigma factors, which can result in changes in gene transcription and protein expression. In *B. burgdorferi*, RpoD functions as the housekeeping sigma factor and the alternative sigma factors Rrp2, RpoN, and RpoS direct the expression of many outer surface proteins and proteins associated with increased virulence [18], [26]. We identified RpoA (rna polymerase), RpoB (rna polymerase), RpoC (rna polymerase), Sigma factor 70 (RpoD) and RpoS (alternative sigma factor), as well as accessory proteins Rrp1 and Rrp2 (response regulators). We also identified 55 proteins whose gene transcripts were previously shown to be regulated by Rrp2, RpoN, and RpoS (Table S6). Fifteen of these proteins overlap with the 39 proteins that increased abundance in the RpH culture relative to the RT culture. Notably, decorin binding proteins DbpA and DbpB, as well as OspC and CheX (chemotaxis operon protein) are transcriptionally regulated by the alternative sigma factors [27], and levels increased in the RpH culture. The abundance patterns for the proteins transcriptionally controlled by the alternative sigma factors show discrete abundance patterns for RpH, RT and Log cultures (Figure 7). These results are consistent with our *in vitro* minimal model of *B. burgdorferi* that assumes a phenotype similar to what is required for host infection and associated with increased bacterial virulence [3]. Genes differentially regulated by Rrp2, RpoN, and RpoS are predominantly present on the main chromosome where we identified 24 proteins out of 34 possible regulated gene products. In plasmid lp54, these sigma factors control 21 genes of which we observed 15 corresponding proteins; OspA, OspB, BBA74 (Oms28), DbpA and DbpB were the most abundantly expressed. The presence of lipoproteins OspA and OspB, adherence proteins DbpA and DbpB [26], and outer membrane-spanning (Oms 28) protein implicate this plasmid as important for *B. burgdorferi* survival in both tick and mammalian hosts [28]. Consistent with previous reports BBA74, a virulent strain-associated outer membrane-spanning protein (Oms 28) shows a reduced abundance in RpH cultures relative to the RT and Log cultures, which further verifies our proteomics results. Moreover, reduced levels of BBA74 are due to transcriptional repression via RpoS [28], which we also identified in the RpH cultures.

### Lipoproteins

In *B. burgdorferi*, 137 predicted lipoproteins [19], [29] represent the most prominent proteins in the total membrane protein profile [30]. We identified 94 of the 137 lipoproteins, 54 of which encoded on the chromosome, and 40 on the plasmids. The most highly abundant lipoproteins identified were OspA, OspB, OspC, OspD, OppA (-I, -II, -III, and -IV), LA-7, DbpA, and BB0028. It was previously demonstrated that OspD expression is influenced by host derived factors i.e. blood being present in *in-vitro* transcriptomics studies [16], [24]. A subset of these lipoproteins in the RpH shows changes in abundance relative to the RT cultures, which reflects biological adaptation to environment change (Figure 5A). Differential expression of lipoproteins in *B. burgdorferi* hallmarks the transition to life inside the mammalian host [8]. Interestingly, we found that lipoprotein BB0028 is abundant in all cultures; however, its biological function remains unknown. Other lipoproteins we identified from lp54 are BBA60, BBA72, BBA74, and members of the paralogous gene family 54 (e.g., BBA64, BBA65, BBA66 and BBA73). Note, BBA65, BBA66, BBA71, and BBA73 were identified as membrane associated and cell surface localized proteins because they partitioned into the Triton X-114 detergent phase and were sensitive to protease treatment of intact cells [31]. Several of these lipoproteins, namely BBA65, BBA66, BBA71, BBA73, were detected previously in infectious *B. burgdorferi* B31 isolates, but not in non-infectious isolates [31]. Importantly, we observed DbpA and DbpB at increased levels in the RpH versus RT cultures. These lipoproteins are involved in mammalian cell attachment and expression is enhanced after mammalian host adaptation [32].

### Chromosome and plasmid usage analysis

The number of encoded proteins identified varied between the chromosome and plasmids (Figure 3). On average, we identified 61% of the proteins encoded by genes on the chromosome, but only 12% of the proteins encoded by genes on the plasmids. Among the plasmids, expression varied dramatically; for instance, we identified 34% and 54% of proteins encoded on lp54 and cp26, respectively, whereas proteins from the other 16 plasmids varied from 4% to 18%. The temperature shift from 23°C to 34°C led to increased protein expression on lp54 (11% increase in theoretical proteome expression, see Figure 3), more than on the chromosome or any other plasmid.

Plasmid cp26 encodes proteins with functions critical for survival and shares some features with the chromosome [33] in that it encodes housekeeping and virulence genes. Of note, OspC is the most abundantly expressed protein from cp26 that along with abundant proteins OppA IV, BBB29, and ResT confer vital function for maintaining cp26 plasmid stability both *in vitro* and *in vivo* [33]. Ten Erp proteins were identified, many Erp proteins bind host factor H, a regulator of complement activation, and thereby help protect the bacterium from the alternative pathway of complement-mediated killing [34], [35].

### Conclusions

This study demonstrates that complementing genomics with proteomics can enhance our understanding of *B. burgdorferi* response to changing *in vitro* culture conditions. Notably, the proteome information derived from different culture conditions provides an initial baseline for studies of systematic adaptations of the proteome that occur as *B. burgdorferi* moves from one environment to another. For this adaptation we observed a significant increase in abundance of many outer surface exposed proteins (OspC, LA7, P66, P83 and P35 antigens), decreased levels of ribosomal and metabolism proteins, and several hypothetical proteins with unknown Lyme disease functions. Additionally, the adhesins DbpA and DbpB and chemotaxis, CheX and CheW proteins showed an increase in abundance in culture conditions that resemble the pH and temperature in a fed tick vector (34°C, pH 6.6). Observations for the *in vitro* model of fed tick vector relative to the unfed tick vector reveal abundance changes in multiple key proteins that lead to changes in *B. burgdorferi* cell membrane, cell metabolism, and other basic functions, which enable adaption to its new environment. Of particular interest is the observed reduction of proteins in the RpH culture that coincides with an observed reduction in ribosomal proteins. These changes directly reflect the slowed growth rate for the bacterium cultured at lowered pH, and we believe that these culture conditions result in *B. burgdorferi* taking on a quiescent phenotype similar to one resulting in persistent human infection. Our studies were limited to only two (pH and temperature) of a myriad of cues that the organism is exposed to as it goes from the tick vector to the mammalian host. Future studies will extend this work to *in situ*, tick-centric proteome studies of *B. burgdorferi* isolated from the tick midgut at different stages of tick feeding.

## Materials and Methods

A low passage and culture adapted B31 strain of *B. burgdorferi* was used for these experiments. Strains grown under both conditions have been studied extensively and found to be both infectious and contained all of the plasmids identified in the genomic sequence of the organism. The cultures were grown *in vitro* in 5% CO_2_ at 34°C in BSK H (Barbour-Stoener-Kelly H) medium supplemented with 6% rabbit serum (Sigma, Saint Louis, MO) to mid-logarithmic stage (2×10^7^ cells/mL). To adapt spirochetes to culture conditions at 23°C, spirochetes first were diluted to 0.5×10^6^ cells/mL and grown to 5×10^7^ cells/mL. The 23°C-adapted spirochetes were inoculated at a final concentration of 1×10^3^ cells/mL and then incubated at a) 34°C and pH 6.6 for the *in vitro* model for *B. burgdorferi* growth in the fed tick midgut, b) 23°C and pH 7.6 for the *in vitro* model for *B. burgdorferi* growth in the unfed tick midgut, c) 34°C and pH 7.6 for the log, stationary and low passage cultures, each culture was grown to cell densities of 5×10^7^ cells/mL for log stage harvest or 2×10^8^ cells/mL for the stationary and low passage cultures respectively. At harvest pH was measured and remained unchanged (with the exception of stationary culture changing −0.2 pH units). Spirochetes were harvested at log and stationary phases by centrifugation, re-suspended in PBS, and then stored at -20°C until use.

### Protein digestion

A global digest was performed on the whole cell lysate for each sample. The sample material was suspended in an equal volume of 100 mM NH_4_HCO_3_ buffer. The resulting suspension (in 200- µL aliquots) was transferred to a 2.0-mL cryovial (with O-ring in cap), and 0.1-mm zirconia/silica disruption beads (BioSpec Products, Bartlesville, OK) were added to equal approximately half of the total volume in the tube. The tube was vigorously vortexed for 30 s and then cooled for 1 min at 4°C in a cold-block. The vortexing step was repeated five times, with a final cooling time of 5 min to reduce any possible aerosols that might contain pathogens. The solution was drawn off the top of the beads and transferred to a 2.0-mL low-binding micro centrifuge tube. A 100- µL aliquot of buffer was added to the beads as a rinse, and the tube was briefly vortexed and cooled for 1 min. The rinse was drawn off of the beads and transferred to the micro centrifuge tube that contained the original lysate. The rinse step was performed two more times, after which the rinse solution was clear. A protein assay (either bicinchoninic acid BCA(Pierce, Rockford, IL) or Coomassie Plus (Pierce, Rockford, IL) was performed on the resultant lysate, and the volume was noted. Urea and thiourea were added to the sample to obtain a solute concentration of 7 M and 2 M, respectively. A 50-mM solution of dithiothreitol (DTT) was used to obtain a 5 mM concentration in the sample. The sample was incubated at 60°C for 30 min to assist with denaturation and reduction of the proteins. The sample was diluted 10-fold with 100 mM NH_4_HCO_3_ buffer, after which trypsin was added in a 1∶50 (w:w) enzyme:protein ratio followed by CaCl_2_ to a final concentration of 1 mM. The sample was incubated for 3 h at 37°C, quick frozen to stop the digestion, and thawed. Solid phase extraction (SPE) cleanup was performed to prepare the sample for LC-MS/MS analysis. A Discovery C-18 SPE column (Supelco, Bellefonte, PA) was conditioned with 2 mL methanol and 3 mL of 0.1% trifluoroacetic acid TFA in water. After the sample was introduced onto the column, the column was rinsed with 4 mL of 95∶5 water:acetonitrile (ACN) with 0.1% TFA. The sample was eluted using 80∶20 acetonitrile:water with 0.1% TFA, and then concentrated in a Savant Speed-vac (ThermoFisher, Milford, MA) to ∼100 µL before performing a BCA protein assay to determine the final sample peptide concentration.

### SCX fractionation and proteome analysis

Tryptic peptides were re-susupended in 900 µL of 10 mM ammonium formate (pH 3.0)/25% ACN and fractionated by SCX chromatography. The Polysulfoethyl 2.1 mm × 200 mm, 5 µm particle size column (PolyLC, Columbia, MD) was preceded by a 2.1 mm × 10 mm guard column on an Agilent 1100 system (Agilent, Palo Alto, CA) equipped with a quaternary pump, degasser, diode array detector, and peltier-cooled autosampler, and fraction collector (both set at 4°C). Mobile phases consisted of (A) 10 mM ammonium formate, 25% ACN, pH 3.0 and (B) 500 mM ammonium formate, 25% ACN, pH 6.8. The gradient was achieved by maintaining mobile phase A at 100% for the first 10 min and then increasing mobile phase B from 0 to 50% over the next 40 min and from 50 to 100% over the following 10 min before sustaining 100% mobile phase B for a final 10 min. A flow rate of 0.2 mL/min was maintained throughout the gradient separation. Spectra were obtained at 280 nm, and fractions were collected over the first 70 min. 14 fractions were collected for each separation, and each fraction was dried under vacuum. The dried fractions were dissolved in 30 µL of 25 mM NH_4_HCO_3_ and 10 µL of each fraction were analyzed using LC-MS/MS. The analytical platform coupled a constant pressure (5000 psi) capillary LC system (75 µm i.d. ×360 µm o.d. ×65 cm capillary; Polymicro reversed phase Technologies Inc., Phoenix, AZ) with an LTQ ion trap mass spectrometer (ThermoFinnigan, San Jose, CA) and an electrospray ionization source manufactured in-house. The instrument was operated in data-dependent mode with an *m/z* range of 400–2000. The 10 most abundant ions from MS analysis were selected for further MS/MS analysis, using a normalized collision energy setting of 35%. A dynamic exclusion of 1 min was applied to reduce repetitive analysis of the same abundant precursor ion. For semi-quantitative analysis of unfractionated cell culture samples, tryptic peptides were prepared as described above and concentrations were determined by BCA assay. We analyzed 3.5 µg peptides for each culture condition, using the same approach described above for analyzing SCX fractions.

### Data analysis

ExtractMSn (version 4.0) and SEQUEST analysis software (Version v.27, Rev 12, Thermo Fisher Scientific, Waltham, MA) were employed to match MS/MS fragmentation spectra to sequences from the *B. burgdorferi* B31 protein database [36] that contains 1737 protein entries. The search was performed using default parameters with no-enzyme rules within a +/- 1.5 Da parent mass window, +/- 0.5 fragment mass window, average parent mass, and monoisotopic fragment mass. Criteria for filtering SEQUEST search results, were retention of: 1) fully tryptic peptide identifications provided ΔCn >0.1 and XCorr >1.5 for peptides assigned 1+ charge state, >1.9 for 2+ charge state, and >2.9 for 3+ charge state; and 2) partially tryptic peptides provided ΔCn >0.1 and XCorr >3.1 for peptides assigned 1+ charge state, >3.8 for 2+ charge state, and >4.7 for 3+ charge state. These criteria were selected based on a method that utilizes a reverse database false positive model [37]. Briefly, a false discovery rate of <1% was determined by searching a decoy reverse database created from the *B. burgdorferi* B31 protein database. The relative protein abundance was estimated based on the number of times peptides were identified for each given protein (referred to as spectral counting). An identified protein was required to be covered by ≥2 unique peptides to be considered for protein abundance comparisons. Clustering and statistical analysis were performed using DAnTE [38].

## Supporting Information

Figure S1 Representative base peak chromatograms from LC-MS/MS analysis of 3.5 µg of tryptic peptides from each cell culture sample condition. The similarity of the base peak chromatogram intensities across all analyses indicates that the large differences in the number of proteins identified among cell culture conditions is genuine.
(1.40 MB TIF)

Table S1 Supplementary Table S1
(1.56 MB DOC)

Table S2 Supplementary Table S2
(0.03 MB DOC)

Table S3 Supplementary Table S3
(0.10 MB DOC)

Table S4 Supplementary Table S4
(0.27 MB DOC)

Table S5 Supplementary Table S5
(0.07 MB DOC)

Table S6 Supplementary Table S6
(0.07 MB DOC)
