# Supplementary figures and images for: Proteome Analysis of Borrelia burgdorferi Response to Environmental Change
Source: PLoS One. 2010 Nov 2;5(11):e13800. doi: 10.1371/journal.pone.0013800 (PMC2970547; doi:10.1371/journal.pone.0013800)

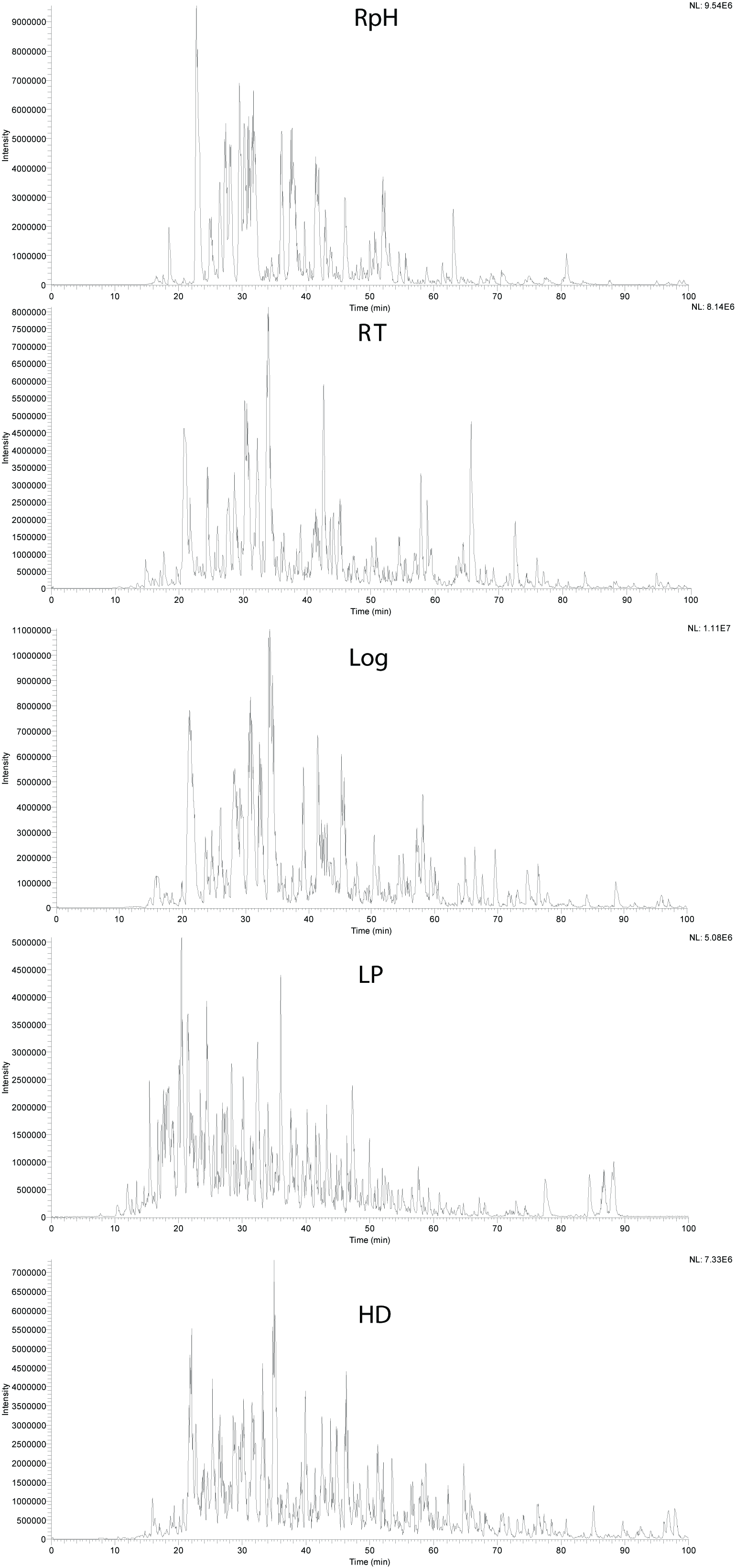

Supplement: Figure S1 — Representative base peak chromatograms from LC-MS/MS analysis of 3.5 µg of tryptic peptides from each cell culture sample condition. The similarity of the base peak chromatogram intensities across all analyses indicates that the large differences in the number of proteins identified among cell culture conditions is genuine. (1.40 MB TIF) [file pone.0013800.s001.tif]
