# Supplementary material for: Proteome Analysis of Borrelia burgdorferi Response to Environmental Change
Source: PLoS One. 2010 Nov 2;5(11):e13800. doi: 10.1371/journal.pone.0013800 (PMC2970547; doi:10.1371/journal.pone.0013800)
Supplement: Table S1 — Supplementary Table S1 (1.56 MB DOC) [file pone.0013800.s002.doc]

| heat shock protein | groEL | Protein fate | BB0649 | 9702 | 164 | x | x | x | x | x | x |
| --- | --- | --- | --- | --- | --- | --- | --- | --- | --- | --- | --- |
| outer surface protein B | ospB | Cell envelope | BBA16 | 7994 | 135 | x | x | x | x | x | x |
| glyceraldehyde 3-phosphate dehydrogenase | gap | Energy metabolism | BB0057 | 4658 | 96 | x | x | x | x | x | x |
| flagellar filament 41 kDa core protein | flaB | Cellular processes | BB0147 | 3868 | 105 | x | x | x | x | x | x |
| enolase | eno | Energy metabolism | BB0337 | 3658 | 104 | x | x | x | x | x | x |
| chaperonin | groES | Protein fate | BB0741 | 3637 | 35 | x | x | x | x | x | x |
| translation elongation factor TU | tuf | Protein synthesis | BB0476 | 3303 | 71 | x | x | x | x | x | x |
| outer surface protein A | ospA | Cell envelope | BBA15 | 3068 | 154 | x | x | x | x | x | x |
| membrane-associated protein p66 |  | Cell envelope | BB0603 | 2567 | 96 | x | x | x | x | x | x |
| outer surface protein C | ospC | Cell envelope | BBB19 | 2412 | 79 | x | x | x | x | x | x |
| DNA gyrase, subunit A | gyrA | DNA metabolism | BB0435 | 2154 | 141 | x | x | x | x | x | x |
| pyruvate kinase | pyk | Energy metabolism | BB0348 | 2024 | 90 | x | x | x | x | x | x |
| aminopeptidase I | yscI | Protein fate | BB0366 | 1834 | 104 | x | x | x | x | x | x |
| heat shock protein 70 | dnaK-2 | Protein fate | BB0518 | 1630 | 119 | x | x | x | x | x | x |
| DNA-directed RNA polymerase | rpoA | Transcription | BB0502 | 1612 | 78 | x | x | x | x | x | x |
| oligopeptide ABC transporter, periplasmic oligopeptide-binding protein | oppA-3 | Transport and binding proteins | BB0330 | 1563 | 110 | x | x | x | x | x | x |
| heat shock protein 90 | htpG | Protein fate | BB0560 | 1379 | 147 | x | x | x | x | x | x |
| DNA-directed RNA polymerase | rpoC | Transcription | BB0388 | 1318 | 197 | x | x | x | x | x | x |
| phosphoglycerate kinase | pgk | Energy metabolism | BB0056 | 1241 | 63 | x | x | x | x | x | x |
| glycerol kinase | glpK | Energy metabolism | BB0241 | 1181 | 74 | x | x | x | x | x | x |
| basic membrane protein A | bmpA | Cell envelope | BB0383 | 1154 | 59 | x | x | x | x | x | x |
| DNA-directed RNA polymerase | rpoB | Transcription | BB0389 | 1111 | 147 | x | x | x | x | x | x |
| translation elongation factor G | fus-1 | Protein synthesis | BB0540 | 1028 | 90 | x | x | x | x | x | x |
| periplasmic serine protease DO | htrA | Protein fate | BB0104 | 974 | 65 | x | x | x | x | x | x |
| oligopeptide ABC transporter, periplasmic oligopeptide-binding protein | oppA-2 | Transport and binding proteins | BB0329 | 950 | 81 | x | x | x | x | x | x |
| oligopeptide ABC transporter, periplasmic oligopeptide-binding protein | oppA-1 | Transport and binding proteins | BB0328 | 941 | 107 | x | x | x | x | x | x |
| antigen, p83/100 |  | Cell envelope | BB0744 | 894 | 143 | x | x | x | x | x | x |
| hypothetical protein |  |  | BB0323 | 891 | 93 | x | x | x | x | x | x |
| fructose-bisphosphate aldolase | fba | Energy metabolism | BB0445 | 864 | 41 | x | x | x | x | x | x |
| pyrophosphate--fructose 6-phosphate 1-phosphotransferase, beta subunit | pfpB | Energy metabolism | BB0020 | 863 | 59 | x | x | x | x | x | x |
| lipoprotein LA7 |  | Cell envelope | BB0365 | 812 | 27 | x | x | x | x | x | x |
| basic membrane protein D | bmpD | Cell envelope | BB0385 | 785 | 47 | x | x | x | x | x | x |
| glycerol-3-phosphate dehydrogenase, anaerobic | glpA | Central intermediary metabolism | BB0243 | 779 | 104 | x | x | x | x | x | x |
| outer surface protein D | ospD | Cell envelope | BBJ09 | 723 | 69 | x | x | x | x | x | x |
| triosephosphate isomerase |  | Energy metabolism | BB0055 | 710 | 39 | x | x | x | x | x | x |
| oligopeptide ABC transporter, periplasmic oligopeptide-binding protein | oppAIV | Transport and binding proteins | BBB16 | 694 | 70 | x | x | x | x | x | x |
| flagellar filament outer layer protein | flaA | Cellular processes | BB0668 | 677 | 46 | x | x | x | x | x | x |
| polyribonucleotide nucleotidyltransferase | pnpA | Transcription | BB0805 | 671 | 96 | x | x | x | x | x | x |
| phosphoenolpyruvate-protein phosphatase | ptsI | Transport and binding proteins | BB0558 | 644 | 69 | x | x | x | x | x | x |
| glycine betaine, L-proline ABC transporter, glycine/betaine/L-proline-binding protein | proX | Transport and binding proteins | BB0144 | 639 | 41 | x | x | x | x | x | x |
| outer membrane porin | oms28 | Transport and binding proteins | BBA74 | 636 | 71 | x | x | x | x | x | x |
| ribosomal protein L1 | rplA | Protein synthesis | BB0392 | 618 | 61 | x | x | x | x | x | x |
| ribosomal protein L3 | rplC | Protein synthesis | BB0478 | 587 | 44 | x | x | x | x | x | x |
| ribosomal protein L6 | rplF | Protein synthesis | BB0493 | 583 | 36 | x | x | x | x | x | x |
| 5'-methylthioadenosine/S-adenosylhomocysteine nucleosidase, putative | pfs-1 | Purines, pyrimidines, nucleosides, and nucleotides | BB0375 | 576 | 38 | x | x | x | x | x | x |
| surface-located membrane protein 1 | lmp1 | Cell envelope | BB0210 | 567 | 129 | x | x | x | x | x | x |
| ribosomal protein L7/L12 | rplL | Protein synthesis | BB0390 | 555 | 35 | x | x | x | x | x | x |
| cell division protein | ftsH | Cellular processes | BB0789 | 530 | 88 | x | x | x | x | x | x |
| ribosomal protein S3 | rpsC | Protein synthesis | BB0484 | 524 | 55 | x | x | x | x | x | x |
| ribosomal protein S2 | rpsB | Protein synthesis | BB0123 | 520 | 58 | x | x | x | x | x | x |
| hypothetical protein |  |  | BB0751 | 505 | 74 | x | x | x | x | x | x |
| heat shock protein | hslU | Protein fate | BB0295 | 495 | 69 | x | x | x | x | x | x |
| glucose-6-phosphate isomerase | pgi | Energy metabolism | BB0730 | 485 | 69 | x | x | x | x | x | x |
| phosphoglycerate mutase | gpmA | Energy metabolism | BB0658 | 479 | 50 | x | x | x | x | x | x |
| aminopeptidase II |  | Protein fate | BB0069 | 472 | 59 | x | x | x | x | x | x |
| zinc protease, putative |  | Protein fate | BB0536 | 472 | 98 | x | x | x | x | x | x |
| 5'-methylthioadenosine/S-adenosylhomocysteine nucleosidase, putative | pfs-2 | Purines, pyrimidines, nucleosides, and nucleotides | BB0588 | 460 | 47 | x | x | x | x | x | x |
| hypothetical protein |  |  | BB0238 | 450 | 58 | x | x | x | x | x | x |
| ribosomal protein L4 | rplD | Protein synthesis | BB0479 | 424 | 44 | x | x | x | x | x | x |
| N-acetylglucosamine-6-phosphate deacetylase | nagA | Energy metabolism | BB0151 | 422 | 42 | x | x | x | x | x | x |
| Lambda CII stability-governing protein | hflK | Protein fate | BB0203 | 419 | 39 | x | x | x | x | x | x |
| preprotein translocase subunit | secA | Protein fate | BB0154 | 417 | 97 | x | x | x | x | x | x |
| carboxyl-terminal protease | ctp | Protein fate | BB0359 | 413 | 80 | x | x | x | x | x | x |
| Lambda CII stability-governing protein | hflC | Protein fate | BB0204 | 402 | 67 | x | x | x | x | x | x |
| L-lactate dehydrogenase | ldh | Energy metabolism | BB0087 | 401 | 42 | x | x | x | x | x | x |
| ribosomal protein S8 | rpsH | Protein synthesis | BB0492 | 395 | 35 | x | x | x | x | x | x |
| ribosomal protein S1 | rpsA | Protein synthesis | BB0127 | 393 | 81 | x | x | x | x | x | x |
| IMP dehydrogenase | guaB | Purines, pyrimidines, nucleosides, and nucleotides | BBB17 | 387 | 62 | x | x | x | x | x | x |
| transcription elongation factor | greA | Transcription | BB0132 | 384 | 67 | x | x | x | x | x | x |
| N-utilization substance protein A | nusA | Transcription | BB0800 | 384 | 61 | x | x | x | x | x | x |
| long-chain-fatty-acid CoA ligase |  | Fatty acid and phospholipid metabolism | BB0593 | 378 | 58 | x | x | x | x | x | x |
| nifS protein | nifS | Biosynthesis of cofactors, prosthetic groups, and carriers | BB0084 | 377 | 56 | x | x | x | x | x | x |
| PTS system, maltose and glucose-specific IIABC component | malX | Transport and binding proteins | BBB29 | 376 | 36 | x | x | x | x | x | x |
| V-type ATPase, subunit B | atpB | Energy metabolism | BB0093 | 375 | 50 | x | x | x | x | x | x |
| ribosomal protein L5 | rplE | Protein synthesis | BB0490 | 368 | 35 | x | x | x | x | x | x |
| ribosomal protein S4 | rpsD | Protein synthesis | BB0615 | 366 | 39 | x | x | x | x | x | x |
| spermidine/putrescine ABC transporter, spermidine/putrescine-binding periplasmic protein | potD | Transport and binding proteins | BB0639 | 364 | 53 | x | x | x | x | x | x |
| ribosomal protein L2 | rplB | Protein synthesis | BB0481 | 359 | 33 | x | x | x | x | x | x |
| ribosomal protein S6 | rpsF | Protein synthesis | BB0115 | 356 | 18 | x | x | x | x | x | x |
| serine hydroxymethyltransferase | glyA | Amino acid biosynthesis | BB0601 | 342 | 45 | x | x | x | x | x | x |
| conserved hypothetical protein |  | Hypothetical proteins | BB0504 | 337 | 65 | x | x | x | x | x | x |
| transcription termination factor Rho | rho | Transcription | BB0230 | 335 | 67 | x | x | x | x | x | x |
| hypothetical protein |  |  | BB0028 | 335 | 40 | x | x | x | x | x | x |
| flagellar hook protein | flgE | Cellular processes | BB0283 | 332 | 34 | x | x | x | x | x | x |
| vacuolar X-prolyl dipeptidyl aminopeptidase I | pepX | Protein fate | BB0627 | 329 | 45 | x | x | x | x | x | x |
| ribosomal protein L9 | rplI | Protein synthesis | BB0112 | 328 | 32 | x | x | x | x | x | x |
| rod shape-determining protein | mreB-1 | Cell envelope | BB0715 | 327 | 52 | x | x | x | x | x | x |
| aminoacyl-histidine dipeptidase | pepD | Protein fate | BB0608 | 320 | 51 | x | x | x | x | x | x |
| conserved hypothetical protein |  | Hypothetical proteins | BB0696 | 319 | 17 | x | x | x | x | x | x |
| ribosomal protein L31 | rpmE | Protein synthesis | BB0229 | 313 | 19 | x | x | x | x | x | x |
| ribosomal protein L16 | rplP | Protein synthesis | BB0485 | 312 | 15 | x | x | x | x | x | x |
| pyrophosphate--fructose 6-phosphate 1-phosphotransferase | pfk | Energy metabolism | BB0727 | 311 | 39 | x | x | x | x | x | x |
| basic membrane protein |  | Cell envelope | BB0108 | 307 | 67 | x | x | x | x | x | x |
| decorin binding protein A | dbpA | Cell envelope | BBA24 | 294 | 42 | x | x | x | x | x | x |
| ribosomal protein S9 | rpsI | Protein synthesis | BB0338 | 290 | 19 | x | x | x | x | x | x |
| conserved hypothetical protein |  | Hypothetical proteins | BB0713 | 285 | 57 | x | x | x | x | x | x |
| isoleucyl-tRNA synthetase | ileS | Protein synthesis | BB0833 | 277 | 88 | x | x | x | x | x | x |
| ribosomal protein L10 | rplJ | Protein synthesis | BB0391 | 271 | 35 | x | x | x | x | x | x |
| ribosomal protein L11 | rplK | Protein synthesis | BB0393 | 270 | 36 | x | x | x | x | x | x |
| oligopeptide ABC transporter, ATP-binding protein | oppF | Transport and binding proteins | BB0335 | 268 | 39 | x | x | x | x | x | x |
| hypothetical protein |  |  | BB0739 | 267 | 18 | x | x | x | x | x | x |
| ribosomal protein S7 | rpsG | Protein synthesis | BB0386 | 266 | 30 | x | x | x | x | x | x |
| arginyl-tRNA synthetase | argS | Protein synthesis | BB0594 | 265 | 56 | x | x | x | x | x | x |
| neutrophil activating protein | napA | Cellular processes | BB0690 | 263 | 30 | x | x | x | x | x | x |
| RNA polymerase sigma-70 factor | rpoD | Transcription | BB0712 | 263 | 64 | x | x | x | x | x | x |
| cell division protein | ftsZ | Cellular processes | BB0299 | 262 | 38 | x | x | x | x | x | x |
| methyl-accepting chemotaxis protein | mcp-2 | Cellular processes | BB0596 | 262 | 48 | x | x | x | x | x | x |
| ribosomal protein S5 | rpsE | Protein synthesis | BB0495 | 261 | 28 | x | x | x | x | x | x |
| hypothetical protein |  |  | BB0752 | 260 | 57 | x | x | x | x | x | x |
| nucleotide sugar epimerase |  | Energy metabolism | BB0444 | 255 | 35 | x | x | x | x | x | x |
| ATP-dependent protease LA | lon-1 | Protein fate | BB0253 | 254 | 69 | x | x | x | x | x | x |
| transcription antitermination factor | nusG | Transcription | BB0394 | 253 | 29 | x | x | x | x | x | x |
| methyl-accepting chemotaxis protein | mcp-3 | Cellular processes | BB0597 | 251 | 60 | x | x | x | x | x | x |
| ATP-dependent Clp protease, subunit X | clpX | Protein fate | BB0612 | 247 | 54 | x | x | x | x | x | x |
| leucyl-tRNA synthetase | leuS | Protein synthesis | BB0251 | 247 | 65 | x | x | x | x | x | x |
| hypothetical protein |  |  | BB0039 | 244 | 60 | x | x | x | x | x | x |
| DNA gyrase, subunit B | gyrB | DNA metabolism | BB0436 | 241 | 54 | x | x | x | x | x | x |
| ribosome releasing factor | frr | Protein synthesis | BB0121 | 239 | 41 | x | x | x | x | x | x |
| V-type ATPase, subunit A | atpA | Energy metabolism | BB0094 | 235 | 40 | x | x | x | x | x | x |
| ABC transporter, ATP-binding protein |  | Transport and binding proteins | BB0754 | 233 | 42 | x | x | x | x | x | x |
| conserved hypothetical protein |  | Hypothetical proteins | BB0228 | 228 | 80 | x | x | x | x | x | x |
| ABC transporter, ATP-binding protein |  | Transport and binding proteins | BB0742 | 227 | 41 | x | x | x | x | x | x |
| phosphate ABC transporter, periplasmic phosphate-binding protein | pstS | Transport and binding proteins | BB0215 | 226 | 37 | x | x | x | x | x | x |
| signal recognition particle protein | ffh | Protein fate | BB0694 | 225 | 56 | x | x | x | x | x | x |
| oligopeptide ABC transporter, ATP-binding protein | oppD | Transport and binding proteins | BB0334 | 225 | 38 | x | x | x | x | x | x |
| stage V sporulation protein G |  | Cellular processes | BB0785 | 220 | 12 | x | x | x | x | x | x |
| protein-export membrane protein | secD | Protein fate | BB0652 | 217 | 46 | x | x | x | x | x | x |
| hypothetical protein |  |  | BB0429 | 213 | 21 | x | x | x | x | x | x |
| hypothetical protein |  |  | BBJ34 | 209 | 49 | x | x | x | x | x | x |
| ribosomal protein L13 | rplM | Protein synthesis | BB0339 | 208 | 25 | x | x | x | x | x | x |
| ATP-dependent helicase | hrpA | DNA metabolism | BB0827 | 204 | 50 | x | x | x | x | x | x |
| hypothetical protein |  |  | BB0426 | 201 | 29 | x | x | x | x | x | x |
| ribosomal protein S13 | rpsM | Protein synthesis | BB0500 | 197 | 28 | x | x | x | x | x | x |
| ribosomal protein S11 | rpsK | Protein synthesis | BB0501 | 191 | 18 | x | x | x | x | x | x |
| flagellar motor switch protein | fliN | Cellular processes | BB0277 | 190 | 11 | x | x | x | x | x | x |
| MoxR-related protein |  | Unknown function | BB0176 | 190 | 37 | x | x | x | x | x | x |
| hypothetical protein |  |  | BB0212 | 190 | 51 | x | x | x | x | x | x |
| hypothetical protein |  |  | BBA68 | 190 | 40 | x | x | x | x | x | x |
| oligoendopeptidase F | pepF | Protein fate | BB0248 | 189 | 49 | x | x | x | x | x | x |
| hypothetical protein |  |  | BB0512 | 189 | 105 | x | x | x | x | x | x |
| spoIIIJ-associtated protein | jag | Unknown function | BB0443 | 188 | 32 | x | x | x | x | x | x |
| superoxide dismutase | sodA | Cellular processes | BB0153 | 187 | 21 | x | x | x | x | x | x |
| chemotaxis response regulator | cheY-2 | Cellular processes | BB0570 | 187 | 19 | x | x | x | x | x | x |
| glucosamine-6-phosphate isomerase | nagB | Energy metabolism | BB0152 | 187 | 29 | x | x | x | x | x | x |
| peptidase, putative |  | Protein fate | BB0067 | 183 | 38 | x | x | x | x | x | x |
| adenine phosphoribosyltransferase | apt | Purines, pyrimidines, nucleosides, and nucleotides | BB0777 | 181 | 18 | x | x | x | x | x | x |
| 3-hydroxy-3-methylglutaryl-CoA synthase |  | Fatty acid and phospholipid metabolism | BB0683 | 180 | 33 | x | x | x | x | x | x |
| ribosomal protein L15 | rplO | Protein synthesis | BB0497 | 180 | 27 | x | x | x | x | x | x |
| glutamyl-tRNA synthetase | gltX | Protein synthesis | BB0372 | 179 | 36 | x | x | x | x | x | x |
| phosphocarrier protein HPr | ptsH-2 | Transport and binding proteins | BB0557 | 179 | 15 | x | x | x | x | x | x |
| methionyl-tRNA formyltransferase | fmt | Protein synthesis | BB0064 | 177 | 30 | x | x | x | x | x | x |
| chemotaxis response regulator | cheY-3 | Cellular processes | BB0672 | 176 | 19 | x | x | x | x | x | x |
| translation elongation factor TS | tsf | Protein synthesis | BB0122 | 176 | 44 | x | x | x | x | x | x |
| hypothetical protein |  |  | BB0820 | 175 | 14 | x | x | x | x | x | x |
| conserved hypothetical protein |  | Hypothetical proteins | BB0068 | 174 | 38 | x | x | x | x | x | x |
| hypothetical protein |  |  | BB0032 | 173 | 46 | x | x | x | x | x | x |
| mannose-6-phosphate isomerase | manA | Energy metabolism | BB0407 | 172 | 31 | x | x | x | x | x | x |
| seryl-tRNA synthetase | serS | Protein synthesis | BB0226 | 172 | 45 | x | x | x | x | x | x |
| flagellar motor switch protein | fliG-2 | Cellular processes | BB0290 | 171 | 42 | x | x | x | x | x | x |
| PTS system, fructose-specific IIABC component | fruA-2 | Transport and binding proteins | BB0629 | 171 | 38 | x | x | x | x | x | x |
| asparaginyl-tRNA synthetase | asnS | Protein synthesis | BB0101 | 170 | 48 | x | x | x | x | x | x |
| ribosomal protein L23 | rplW | Protein synthesis | BB0480 | 170 | 26 | x | x | x | x | x | x |
| hypothetical protein |  |  | BBB28 | 170 | 45 | x | x | x | x | x | x |
| conserved hypothetical protein |  | Hypothetical proteins | BB0047 | 169 | 27 | x | x | x | x | x | x |
| recA protein | recA | DNA metabolism | BB0131 | 168 | 29 | x | x | x | x | x | x |
| heat shock protein | dnaJ-1 | Protein fate | BB0517 | 168 | 32 | x | x | x | x | x | x |
| translation initiation factor 2 | infB | Protein synthesis | BB0801 | 167 | 50 | x | x | x | x | x | x |
| ribosomal protein L24 | rplX | Protein synthesis | BB0489 | 166 | 8 | x | x | x | x | x | x |
| lysyl-tRNA synthetase |  | Protein synthesis | BB0659 | 166 | 42 | x | x | x | x | x | x |
| DNA polymerase III, subunit alpha | dnaE | DNA metabolism | BB0579 | 165 | 56 | x | x | x | x | x | x |
| hypothetical protein |  |  | BB0749 | 165 | 47 | x | x | x | x | x | x |
| glycyl-tRNA synthetase | glyS | Protein synthesis | BB0371 | 164 | 39 | x | x | x | x | x | x |
| GMP synthase | guaA | Purines, pyrimidines, nucleosides, and nucleotides | BBB18 | 163 | 37 | x | x | x | x | x | x |
| valyl-tRNA synthetase | valS | Protein synthesis | BB0738 | 162 | 51 | x | x | x | x | x | x |
| grpE protein | grpE | Protein fate | BB0519 | 160 | 36 | x | x | x | x | x | x |
| PTS system, glucose-specific IIA component | crr | Transport and binding proteins | BB0559 | 160 | 21 | x | x | x | x | x | x |
| aspartyl-tRNA synthetase | aspS | Protein synthesis | BB0446 | 159 | 41 | x | x | x | x | x | x |
| penicillin-binding protein | pbp-3 | Cell envelope | BB0732 | 157 | 49 | x | x | x | x | x | x |
| ATP-dependent Clp protease proteolytic component | clpP-1 | Protein fate | BB0611 | 157 | 17 | x | x | x | x | x | x |
| ribosomal protein L17 | rplQ | Protein synthesis | BB0503 | 157 | 17 | x | x | x | x | x | x |
| methionyl-tRNA synthetase | metG | Protein synthesis | BB0587 | 156 | 44 | x | x | x | x | x | x |
| ribosomal protein L21 | rplU | Protein synthesis | BB0778 | 155 | 18 | x | x | x | x | x | x |
| long-chain-fatty-acid CoA ligase |  | Fatty acid and phospholipid metabolism | BB0137 | 154 | 43 | x | x | x | x | x | x |
| hypothetical protein |  |  | BB0553 | 154 | 53 | x | x | x | x | x | x |
| conserved hypothetical integral membrane protein |  | Hypothetical proteins | BB0252 | 153 | 33 | x | x | x | x | x | x |
| phenylalanyl-tRNA synthetase, beta subunit | pheT | Protein synthesis | BB0514 | 153 | 48 | x | x | x | x | x | x |
| flagellum-specific ATP synthase | fliI | Cellular processes | BB0288 | 152 | 31 | x | x | x | x | x | x |
| S-adenosylmethionine synthetase | metK | Central intermediary metabolism | BB0376 | 152 | 31 | x | x | x | x | x | x |
| hypothetical protein |  |  | BBI39 | 152 | 21 | x | x | x | x | x | x |
| trigger factor | tig | Protein fate | BB0610 | 151 | 29 | x | x | x | x | x | x |
| ribosomal protein L18 | rplR | Protein synthesis | BB0494 | 149 | 24 | x | x | x | x | x | x |
| ribosomal protein L20 | rplT | Protein synthesis | BB0188 | 148 | 19 | x | x | x | x | x | x |
| cell division protein | ftsA | Cellular processes | BB0300 | 145 | 24 | x | x | x | x | x | x |
| ribosomal protein S17 | rpsQ | Protein synthesis | BB0487 | 145 | 13 | x | x | x | x | x | x |
| hypothetical protein |  |  | BB0236 | 145 | 41 | x | x | x | x | x | x |
| chemotaxis operon protein | cheX | Unknown function | BB0671 | 143 | 10 | x | x | x | x | x | x |
| conserved hypothetical protein |  | Hypothetical proteins | BBA41 | 142 | 34 | x | x | x | x | x | x |
| ribose 5-phosphate isomerase | rpi | Energy metabolism | BB0657 | 141 | 30 | x | x | x | x | x | x |
| glycerol-3-phosphate O-acyltransferase, putative |  | Fatty acid and phospholipid metabolism | BB0327 | 141 | 29 | x | x | x | x | x | x |
| conserved hypothetical protein |  | Hypothetical proteins | BB0606 | 140 | 31 | x | x | x | x | x | x |
| prolyl-tRNA synthetase | proS | Protein synthesis | BB0402 | 140 | 50 | x | x | x | x | x | x |
| antigen, P35, putative |  | Cell envelope | BBJ41 | 138 | 19 | x | x | x | x | x | x |
| DNA topoisomerase IV | parC | DNA metabolism | BB0035 | 138 | 33 | x | x | x | x | x | x |
| methyl-accepting chemotaxis protein | mcp-5 | Cellular processes | BB0681 | 137 | 36 | x | x | x | x | x | x |
| conserved hypothetical protein |  | Hypothetical proteins | BB0195 | 137 | 33 | x | x | x | x | x | x |
| conserved hypothetical protein |  | Hypothetical proteins | BBK13 | 137 | 30 | x | x | x |  | x |  |
| heat shock protein | hslV | Protein fate | BB0296 | 137 | 19 | x | x | x | x | x | x |
| threonyl-tRNA synthetase | thrZ | Protein synthesis | BB0720 | 137 | 40 | x | x | x | x | x | x |
| basic membrane protein B | bmpB | Cell envelope | BB0382 | 136 | 22 | x | x | x | x | x | x |
| flagellar hook-associated protein | flgK | Cellular processes | BB0181 | 136 | 45 | x | x | x | x | x | x |
| CTP synthase | pyrG | Purines, pyrimidines, nucleosides, and nucleotides | BB0575 | 134 | 34 | x | x | x | x | x | x |
| ribosomal protein L27 | rpmA | Protein synthesis | BB0780 | 133 | 12 | x | x | x | x | x | x |
| ORFZ01944 |  |  | ORFZ01944 | 133 | 28 | x | x | x | x | x | x |
| acetyl-CoA C-acetyltransferase | fadA | Fatty acid and phospholipid metabolism | BB0109 | 132 | 16 | x | x | x | x | x | x |
| hypothetical protein |  |  | BB0326 | 132 | 49 | x | x | x | x | x | x |
| DNA topoisomerase IV | parE | DNA metabolism | BB0036 | 131 | 45 | x | x | x | x | x | x |
| alanyl-tRNA synthetase | alaS | Protein synthesis | BB0220 | 131 | 43 | x | x | x | x | x | x |
| ribose/galactose ABC transporter, ATP-binding protein | mglA | Transport and binding proteins | BB0677 | 131 | 39 | x | x | x | x | x | x |
| conserved hypothetical protein |  | Hypothetical proteins | BB0628 | 130 | 29 | x | x | x | x | x | x |
| glucose inhibited division protein A | gidA | DNA metabolism | BB0178 | 129 | 39 | x | x | x | x | x | x |
| flagellar-associated GTP-binding protein | flhF | Cellular processes | BB0270 | 128 | 40 | x | x | x | x | x | x |
| methyl-accepting chemotaxis protein | mcp-4 | Cellular processes | BB0680 | 127 | 38 | x | x | x | x | x | x |
| outer surface protein, putative |  | Cell envelope | BBB07 | 126 | 34 | x | x | x | x | x | x |
| V-type ATPase, subunit E, putative |  | Energy metabolism | BB0096 | 126 | 26 | x | x | x | x | x | x |
| ribosomal protein S12 | rpsL | Protein synthesis | BB0387 | 126 | 11 | x | x | x | x | x | x |
| PTS system, fructose-specific IIABC component | fruA-1 | Transport and binding proteins | BB0408 | 125 | 28 | x | x | x | x | x | x |
| membrane fusion protein | mtrC | Cellular processes | BB0141 | 124 | 33 | x | x | x | x | x | x |
| ribosomal protein L22 | rplV | Protein synthesis | BB0483 | 122 | 23 | x | x | x | x | x | x |
| hypothetical protein |  |  | BB0546 | 122 | 23 | x | x | x | x | x | x |
| hypothetical protein |  |  | BB0796 | 121 | 29 | x | x | x | x | x | x |
| histidyl-tRNA synthetase | hisS | Protein synthesis | BB0135 | 119 | 32 | x | x | x | x | x | x |
| outer membrane protein |  | Cell envelope | BBA52 | 118 | 28 | x | x | x | x | x | x |
| flagellar motor switch protein | fliM | Cellular processes | BB0278 | 118 | 33 | x | x | x | x | x | x |
| hypothetical protein |  |  | BBG01 | 118 | 32 | x | x | x | x | x | x |
| tryptophanyl-tRNA synthetase | trsA | Protein synthesis | BB0005 | 117 | 30 | x | x | x | x | x | x |
| hypothetical protein |  |  | BB0058 | 117 | 29 | x | x | x | x | x | x |
| surface lipoprotein P27 |  | Cell envelope | BBA60 | 115 | 36 | x | x | x | x | x | x |
| flagellar basal-body rod protein | fliF | Cellular processes | BB0291 | 114 | 34 | x | x | x | x | x | x |
| chemotaxis histidine kinase | cheA-2 | Cellular processes | BB0669 | 114 | 30 | x | x | x | x | x | x |
| conserved hypothetical protein |  | Hypothetical proteins | BB0651 | 113 | 16 | x | x | x | x | x | x |
| immunogenic protein P37, putative |  | Cell envelope | BBK45 | 112 | 37 | x | x | x |  | x |  |
| hypothetical protein |  |  | BB0267 | 111 | 35 | x | x | x | x | x | x |
| translation initiation factor 3 | infC | Protein synthesis | BB0190 | 110 | 25 | x | x | x | x | x | x |
| ribosomal protein S16 | rpsP | Protein synthesis | BB0695 | 110 | 17 | x | x | x | x | x | x |
| hypothetical protein |  |  | BB0351 | 110 | 34 | x | x | x | x | x | x |
| hypothetical protein |  |  | BB0325 | 107 | 31 | x | x | x | x | x | x |
| ATP-dependent Clp protease, subunit C | clpC | Protein fate | BB0834 | 106 | 44 | x | x | x | x | x | x |
| Glu-tRNA(Gln) amidotransferase, subunit A | gatA | Protein synthesis | BB0342 | 105 | 26 | x | x | x | x | x | x |
| flagellar hook-associated protein 2 | fliD | Cellular processes | BB0149 | 104 | 40 | x | x | x | x | x | x |
| flagellar P-ring protein | flgI | Cellular processes | BB0772 | 103 | 33 | x | x | x | x | x | x |
| ORFZ01806 |  |  | ORFZ01806 | 103 | 13 | x | x | x | x | x | x |
| translation elongation factor G | fus-2 | Protein synthesis | BB0691 | 102 | 30 | x | x | x | x | x | x |
| hypothetical protein |  |  | BBA40 | 102 | 23 | x | x | x | x | x | x |
| DNA polymerase I | polA | DNA metabolism | BB0548 | 101 | 51 | x | x | x | x | x | x |
| acetate kinase | ackA | Energy metabolism | BB0622 | 101 | 26 | x | x | x | x | x | x |
| ATP-dependent Clp protease proteolytic component | clpP-2 | Protein fate | BB0757 | 101 | 25 | x | x | x | x | x | x |
| hypothetical protein |  |  | BB0646 | 101 | 17 | x | x | x | x | x | x |
| ORFZ01955 |  |  | ORFZ01955 | 101 | 38 | x | x | x |  | x |  |
| outer membrane protein |  | Cell envelope | BB0795 | 99 | 33 | x | x | x | x | x | x |
| guanosine-3',5'-bis(diphosphate) 3'-pyrophosphohydrolase | spoT | Regulatory functions | BB0198 | 99 | 32 | x | x | x | x | x | x |
| PTS system, cellobiose-specific IIA component | celC | Transport and binding proteins | BBB05 | 99 | 13 | x | x | x | x | x | x |
| hypothetical protein |  |  | BBA69 | 98 | 34 | x | x | x | x | x | x |
| chromosome segregation protein, putative |  | Cellular processes | BB0431 | 97 | 27 | x | x | x | x | x | x |
| peptide chain release factor 1 | prfA | Protein synthesis | BB0196 | 96 | 31 | x | x | x | x | x | x |
| carboxypeptidase, putative |  | Cell envelope | BB0582 | 95 | 28 | x | x | x | x | x | x |
| outer membrane protein |  | Cell envelope | BBA03 | 95 | 18 | x | x | x | x | x | x |
| ribosomal protein L14 | rplN | Protein synthesis | BB0488 | 95 | 14 | x | x | x | x | x | x |
| conserved hypothetical GTP-binding protein |  | Hypothetical proteins | BB0235 | 94 | 18 | x | x | x | x | x | x |
| V-type ATPase, subunit I, putative |  | Energy metabolism | BB0091 | 93 | 19 | x | x | x | x | x | x |
| hypothetical protein |  |  | BB0103 | 93 | 22 | x | x | x | x | x | x |
| chemotaxis histidine kinase | cheA-1 | Cellular processes | BB0567 | 92 | 31 | x | x | x | x | x | x |
| 1-phosphofructokinase | fruK | Energy metabolism | BB0630 | 92 | 15 | x | x | x | x | x | x |
| conserved hypothetical protein |  | Hypothetical proteins | BB0462 | 92 | 22 | x | x | x | x | x | x |
| Glu-tRNA(Gln) amidotransferase, subunit B | gatB | Protein synthesis | BB0341 | 92 | 29 | x | x | x | x | x | x |
| cysteinyl-tRNA synthetase | cysS | Protein synthesis | BB0599 | 92 | 23 | x | x | x | x | x | x |
| hypothetical protein |  |  | BB0345 | 92 | 31 | x | x | x | x | x | x |
| ribosomal protein S18 | rpsR | Protein synthesis | BB0113 | 91 | 21 | x | x | x | x | x | x |
| decorin binding protein B | dbpB | Cell envelope | BBA25 | 90 | 24 |  | x | x | x | x |  |
| excinuclease ABC, subunit A | uvrA | DNA metabolism | BB0837 | 90 | 38 | x | x | x | x | x | x |
| conserved hypothetical protein |  | Hypothetical proteins | BB0644 | 90 | 20 | x | x | x | x | x | x |
| ribosomal protein L30 | rpmD | Protein synthesis | BB0496 | 90 | 14 | x | x | x | x | x | x |
| ribosomal protein L19 | rplS | Protein synthesis | BB0699 | 89 | 18 | x | x | x | x | x | x |
| UDP-N-acetylglucosamine 1-carboxyvinyltransferase | murA | Cell envelope | BB0472 | 87 | 26 | x | x | x | x | x | x |
| ribosomal protein S15 | rpsO | Protein synthesis | BB0804 | 87 | 15 | x | x | x | x | x | x |
| hypothetical protein |  |  | BB0011 | 87 | 22 | x | x | x | x | x | x |
| hypothetical protein |  |  | BB0142 | 86 | 27 | x | x | x | x | x | x |
| exported protein | tpn38b | Cell envelope | BB0319 | 84 | 19 | x | x | x | x | x | x |
| acriflavine resistance protein | acrB | Cellular processes | BB0140 | 84 | 38 | x | x | x | x | x | x |
| phenylalanyl-tRNA synthetase, alpha subunit | pheS | Protein synthesis | BB0513 | 84 | 27 | x | x | x | x | x | x |
| DNA mismatch repair protein | mutL | DNA metabolism | BB0211 | 83 | 28 | x | x | x | x | x | x |
| dnaK suppressor, putative |  | Unknown function | BB0168 | 83 | 17 | x | x | x | x | x | x |
| hypothetical protein |  |  | BB0458 | 83 | 29 | x | x | x | x | x | x |
| glycerol-3-phosphate dehydrogenase, NAD(P)+ | gpsA | Fatty acid and phospholipid metabolism | BB0368 | 82 | 18 | x | x | x | x | x | x |
| hypothetical protein |  |  | BB0733 | 82 | 17 | x | x | x | x | x | x |
| polypeptide deformylase | def | Protein fate | BB0065 | 81 | 13 | x | x | x | x | x | x |
| hypothetical protein |  |  | BB0034 | 81 | 11 | x | x | x | x | x | x |
| hypothetical protein |  |  | BB0227 | 81 | 24 | x | x | x | x | x | x |
| conserved hypothetical protein |  | Hypothetical proteins | BB0734 | 80 | 26 | x | x | x | x | x | x |
| transcription factor, putative |  | Transcription | BB0355 | 80 | 19 | x | x | x | x | x | x |
| GTP-binding membrane protein | lepA | Unknown function | BB0088 | 80 | 28 | x | x | x | x | x | x |
| flagellar hook-associated protein 3 | flgL | Cellular processes | BB0182 | 79 | 28 | x | x | x | x | x | x |
| phosphogluconate dehydrogenase, decarboxylating | gnd | Energy metabolism | BB0561 | 79 | 26 | x | x | x | x | x | x |
| NADH oxidase, water-forming | nox | Energy metabolism | BB0728 | 79 | 25 | x | x | x | x | x | x |
| ribosome-binding factor A | rbfA | Protein synthesis | BB0802 | 79 | 22 | x | x | x | x | x | x |
| phosphoribosyl pyrophosphate synthetase | prs | Purines, pyrimidines, nucleosides, and nucleotides | BB0544 | 79 | 19 | x | x | x | x | x | x |
| minD-related ATP-binding protein | ylxH-3 | Cellular processes | BB0726 | 78 | 22 | x | x | x | x | x | x |
| heat shock protein 70 | dnaK-1 | Protein fate | BB0264 | 77 | 21 | x | x | x | x | x | x |
| hypothetical protein |  |  | BBI29 | 77 | 23 | x | x | x |  | x |  |
| 4-methyl-5(b-hydroxyethyl)-thiazole monophosphate biosynthesis protein | thiJ | Biosynthesis of cofactors, prosthetic groups, and carriers | BB0621 | 76 | 15 | x | x | x | x | x | x |
| DNA polymerase III, subunit beta | dnaN | DNA metabolism | BB0438 | 76 | 20 | x | x | x | x | x | x |
| conserved hypothetical protein |  | Hypothetical proteins | BB0364 | 76 | 19 | x | x | x | x | x | x |
| DNA topoisomerase I | topA | DNA metabolism | BB0828 | 75 | 39 | x |  | x | x | x |  |
| hypothetical protein |  |  | BB0063 | 75 | 20 | x | x | x | x | x | x |
| hypothetical protein |  |  | BB0246 | 75 | 20 | x | x | x | x | x | x |
| methionine aminopeptidase | map | Protein fate | BB0105 | 74 | 12 | x | x | x | x | x | x |
| ATP-dependent protease LA | lon-2 | Protein fate | BB0613 | 74 | 34 | x |  | x | x | x |  |
| ribosomal protein L28 | rpmB | Protein synthesis | BB0350 | 74 | 16 | x | x | x | x | x | x |
| hypothetical protein |  |  | BB0261 | 72 | 24 | x | x | x | x | x | x |
| conserved hypothetical protein |  | Hypothetical proteins | BB0619 | 70 | 20 | x | x | x | x | x | x |
| conserved hypothetical protein |  | Hypothetical proteins | BB0635 | 70 | 26 | x | x | x | x | x | x |
| ribosomal protein L29 | rpmC | Protein synthesis | BB0486 | 70 | 10 | x | x | x | x | x | x |
| nucleoside-diphosphate kinase | ndk | Purines, pyrimidines, nucleosides, and nucleotides | BB0463 | 70 | 19 | x | x | x | x | x | x |
| response regulatory protein | rrp-2 | Regulatory functions | BB0763 | 70 | 26 | x | x | x | x | x | x |
| glycine betaine, L-proline ABC transporter, ATP-binding protein | proV | Transport and binding proteins | BB0146 | 70 | 23 | x | x | x | x | x | x |
| hypothetical protein |  |  | BB0811 | 70 | 36 | x | x | x | x | x | x |
| conserved hypothetical protein |  | Hypothetical proteins | BB0025 | 69 | 20 | x | x | x | x | x | x |
| oligopeptide ABC transporter, permease protein | oppC-1 | Transport and binding proteins | BB0333 | 69 | 17 | x | x | x | x | x | x |
| hypothetical protein |  |  | BBH06 | 69 | 19 | x | x | x | x | x | x |
| methyl-accepting chemotaxis protein | mcp-1 | Cellular processes | BB0578 | 68 | 32 | x | x | x | x | x | x |
| purine-binding chemotaxis protein | cheW-3 | Cellular processes | BB0670 | 68 | 18 | x | x | x | x | x | x |
| chromosomal replication initiator protein | dnaA | DNA metabolism | BB0437 | 68 | 28 | x | x | x | x | x | x |
| glucose-6-phosphate 1-dehydrogenase, putative |  | Energy metabolism | BB0222 | 68 | 14 | x | x | x | x | x | x |
| conserved hypothetical protein |  | Hypothetical proteins | BB0298 | 68 | 16 | x | x | x | x | x | x |
| peptide chain release factor 2 | prfB | Protein synthesis | BB0074 | 68 | 22 | x | x | x | x | x | x |
| xylose operon regulatory protein | xylR-1 | Regulatory functions | BB0693 | 68 | 18 | x | x | x | x | x | x |
| hypothetical protein |  |  | BB0205 | 68 | 25 | x | x | x | x | x | x |
| hypothetical protein |  |  | BB0664 | 68 | 20 | x | x | x | x | x | x |
| antigen, S2, putative |  | Cell envelope | BB0158 | 66 | 19 | x | x | x | x | x | x |
| N-acetylmuramoyl-L-alanine amidase, putative |  | Cell envelope | BB0625 | 66 | 32 | x | x | x | x | x | x |
| ornithine carbamoyltransferase, catabolic | arcB | Energy metabolism | BB0842 | 66 | 26 | x |  | x | x | x |  |
| lipopolysaccharide biosynthesis-related protein |  | Unknown function | BB0454 | 66 | 24 | x | x | x | x | x | x |
| flagellar protein | fliL | Cellular processes | BB0279 | 65 | 8 | x | x | x | x | x | x |
| signal recognition particle-docking protein FtsY | ftsY | Protein fate | BB0076 | 65 | 22 | x | x | x | x | x | x |
| xylose operon regulatory protein | xylR-2 | Regulatory functions | BB0831 | 65 | 11 | x | x | x | x | x | x |
| ABC transporter, ATP-binding protein |  | Transport and binding proteins | BB0466 | 65 | 17 | x | x | x | x | x | x |
| antigen, P35, putative |  | Cell envelope | BBA66 | 64 | 12 |  | x | x | x | x |  |
| flagellar motor rotation protein B | motB | Cellular processes | BB0280 | 64 | 17 | x | x | x | x | x | x |
| glpE protein | glpE | Energy metabolism | BB0016 | 64 | 18 | x | x | x | x | x | x |
| hypothetical protein |  |  | BB0125 | 64 | 20 | x | x | x | x | x | x |
| inner membrane protein |  | Cell envelope | BB0442 | 63 | 28 | x | x | x | x | x | x |
| hypothetical protein |  | Hypothetical proteins | BBD13 | 63 | 7 | x | x | x | x | x | x |
| DNA recombinase | recG | DNA metabolism | BB0581 | 62 | 24 | x | x | x | x | x | x |
| hypothetical protein |  |  | BB0776 | 62 | 16 | x | x | x | x | x | x |
| rare lipoprotein A | rlpA | Cell envelope | BB0735 | 61 | 17 | x | x | x | x | x | x |
| single-stranded DNA-binding protein | ssb | DNA metabolism | BB0114 | 61 | 17 | x | x | x | x | x | x |
| conserved hypothetical protein |  | Hypothetical proteins | BB0086 | 61 | 26 | x | x | x | x | x | x |
| flagellar protein | flbB | Cellular processes | BB0286 | 60 | 15 | x | x | x | x | x | x |
| thioredoxin | trxA | Energy metabolism | BB0061 | 60 | 9 | x | x | x | x | x | x |
| conserved hypothetical protein |  | Hypothetical proteins | BB0537 | 60 | 20 | x | x | x | x | x | x |
| conserved hypothetical protein |  | Hypothetical proteins | BBN33 | 60 | 18 | x | x | x | x | x | x |
| hypothetical protein |  |  | BB0072 | 60 | 26 | x | x | x | x | x | x |
| pyridoxal kinase | pdxK | Biosynthesis of cofactors, prosthetic groups, and carriers | BB0768 | 58 | 21 | x | x | x | x | x | x |
| tyrosyl-tRNA synthetase | tyrS | Protein synthesis | BB0370 | 58 | 17 | x | x | x | x | x | x |
| pheromone shutdown protein | traB | Regulatory functions | BB0416 | 58 | 20 | x | x | x | x | x | x |
| conserved hypothetical protein |  | Hypothetical proteins | BB0381 | 57 | 15 | x | x | x | x | x | x |
| translation elongation factor P | efp | Protein synthesis | BB0214 | 57 | 13 | x | x | x | x | x | x |
| hypothetical protein |  |  | BBH18 | 57 | 22 | x |  | x | x | x |  |
| stage 0 sporulation protein J | spo0J | Cellular processes | BB0434 | 56 | 21 | x | x | x | x | x | x |
| hbbU protein |  | Protein synthesis | BB0232 | 56 | 17 | x | x | x | x | x | x |
| ribosomal protein L33 | rpmG | Protein synthesis | BB0396 | 56 | 6 | x | x | x | x | x | x |
| deoxyguanosine/deoxyadenosine kinase(I) subunit 2 | dck | Purines, pyrimidines, nucleosides, and nucleotides | BB0239 | 56 | 18 | x | x | x | x | x | x |
| conserved hypothetical protein |  | Hypothetical proteins | BB0525 | 55 | 18 | x | x | x | x | x | x |
| conserved hypothetical protein |  | Hypothetical proteins | BBB22 | 55 | 5 | x | x | x | x | x | x |
| heat shock protein | dnaJ-2 | Protein fate | BB0655 | 55 | 25 | x | x | x | x | x | x |
| ribosomal protein S19 | rpsS | Protein synthesis | BB0482 | 55 | 4 | x | x | x | x | x | x |
| DNA polymerase III, subunits gamma and tau | dnaX | DNA metabolism | BB0461 | 54 | 15 | x |  | x | x | x |  |
| ribosomal protein S20 | rpsT | Protein synthesis | BB0233 | 54 | 8 | x | x | x | x | x | x |
| hypothetical protein |  |  | BB0840 | 54 | 25 | x | x | x | x | x | x |
| ribosomal protein S10 | rpsJ | Protein synthesis | BB0477 | 52 | 4 | x | x | x | x | x | x |
| adenylate kinase | adk | Purines, pyrimidines, nucleosides, and nucleotides | BB0417 | 52 | 23 | x | x | x | x | x | x |
| antigen S2-related protein |  | Cell envelope | BB0159 | 51 | 21 | x | x | x | x | x | x |
| UDP-N-acetylmuramoylalanyl-D-glutamate--2,6-diaminopimelate ligase | murE | Cell envelope | BB0201 | 50 | 23 | x | x | x | x | x | x |
| conserved hypothetical protein |  | Hypothetical proteins | BB0194 | 50 | 14 | x | x | x | x | x | x |
| conserved hypothetical protein |  | Hypothetical proteins | BBM33 | 50 | 18 | x | x | x | x | x | x |
| conserved hypothetical protein |  | Hypothetical proteins | BB0709 | 49 | 13 | x | x | x | x | x | x |
| conserved hypothetical protein |  | Hypothetical proteins | BBH13 | 49 | 14 | x |  | x | x | x |  |
| ribonuclease III | rnc | Transcription | BB0705 | 49 | 11 | x | x | x | x | x | x |
| hypothetical protein |  |  | BB0397 | 49 | 15 | x | x | x | x | x | x |
| hypothetical protein |  |  | BB0689 | 49 | 18 | x | x | x | x | x | x |
| phosphate transport system regulatory protein | phoU | Regulatory functions | BB0042 | 48 | 11 | x | x | x | x | x | x |
| hypothetical protein |  |  | BB0024 | 48 | 25 | x | x | x | x | x | x |
| hypothetical protein |  |  | BB0155 | 48 | 20 | x | x | x | x | x | x |
| glycosyl transferase | lgtD | Cell envelope | BB0572 | 47 | 13 | x | x | x | x | x | x |
| phosphoglucomutase | femD | Energy metabolism | BB0004 | 47 | 19 | x | x | x | x | x | x |
| hypothetical protein |  |  | BB0126 | 47 | 13 | x | x | x | x | x | x |
| P115 protein |  | Unknown function | BB0045 | 46 | 24 | x | x | x | x | x | x |
| hypothetical protein |  |  | BBI16 | 46 | 16 | x | x | x |  | x |  |
| chemotaxis response regulator | cheY-1 | Cellular processes | BB0551 | 45 | 10 | x | x | x | x | x | x |
| arginine deiminase | arcA | Energy metabolism | BB0841 | 45 | 16 | x | x | x | x | x | x |
| phosphate ABC transporter, ATP-binding protein | pstB | Transport and binding proteins | BB0218 | 45 | 13 | x | x | x | x | x | x |
| hypothetical protein |  |  | BB0760 | 45 | 12 | x | x | x | x | x | x |
| flagellar motor rotation protein A | motA | Cellular processes | BB0281 | 44 | 14 | x | x | x | x | x | x |
| hypothetical protein |  |  | BB0133 | 44 | 14 | x | x | x | x | x | x |
| flagellar basal-body rod protein | flgG | Cellular processes | BB0774 | 43 | 12 | x | x | x | x | x | x |
| plasmid partition protein, putative |  | Cellular processes | BBD21 | 43 | 13 | x | x | x | x | x | x |
| conserved hypothetical protein |  | Hypothetical proteins | BB0538 | 43 | 13 | x | x | x | x | x | x |
| hypothetical protein |  |  | BB0532 | 43 | 9 | x | x | x | x | x | x |
| flagellar assembly protein | fliH | Cellular processes | BB0289 | 42 | 10 | x | x | x | x | x | x |
| flagellar hook-basal body complex protein | flhO | Cellular processes | BB0775 | 42 | 18 | x | x | x | x | x | x |
| single-stranded-DNA-specific exonuclease | recJ | DNA metabolism | BB0254 | 42 | 19 | x | x | x | x | x | x |
| conserved hypothetical protein |  | Hypothetical proteins | BB0129 | 42 | 20 | x | x | x | x | x | x |
| conserved hypothetical protein |  | Hypothetical proteins | BBB01 | 42 | 12 | x | x | x | x | x | x |
| oligopeptide ABC transporter, periplasmic oligopeptide-binding protein | oppAV | Transport and binding proteins | BBA34 | 42 | 18 |  | x | x | x | x |  |
| hypothetical protein |  |  | BB0783 | 42 | 15 | x | x | x | x | x | x |
| D-alanine--D-alanine ligase | ddlA | Cell envelope | BB0200 | 41 | 9 | x | x | x | x | x | x |
| conserved hypothetical protein |  | Hypothetical proteins | BB0755 | 41 | 16 | x | x | x | x | x | x |
| hypothetical protein |  |  | BB0038 | 41 | 21 | x |  | x | x | x |  |
| hypothetical protein |  |  | BB0259 | 41 | 23 | x | x | x | x | x | x |
| hypothetical protein |  |  | BB0324 | 41 | 14 | x | x | x | x | x | x |
| UDP-N-acetylmuramate--alanine ligase | murC | Cell envelope | BB0817 | 40 | 15 | x | x | x | x | x | x |
| flagellar biosynthesis protein | flhA | Cellular processes | BB0271 | 40 | 15 | x | x | x | x | x | x |
| protein-glutamate methylesterase | cheB-2 | Cellular processes | BB0568 | 40 | 18 | x | x | x | x | x | x |
| UTP--glucose-1-phosphate uridylyltransferase | gtaB | Energy metabolism | BB0207 | 40 | 17 | x | x | x | x | x | x |
| ferric uptake regulation protein | fur | Regulatory functions | BB0647 | 40 | 17 | x | x | x | x | x | x |
| spermidine/putrescine ABC transporter, ATP-binding protein | potA | Transport and binding proteins | BB0642 | 40 | 12 | x | x | x | x | x | x |
| hypothetical protein |  |  | BB0624 | 40 | 15 | x | x | x | x | x | x |
| octaprenyl-diphosphate synthase | ispB | Biosynthesis of cofactors, prosthetic groups, and carriers | BB0314 | 39 | 14 | x | x | x | x | x | x |
| antigen, P35, putative |  | Cell envelope | BBH32 | 39 | 15 | x |  | x | x | x |  |
| conserved hypothetical protein |  | Hypothetical proteins | BBG33 | 39 | 22 | x | x | x | x | x | x |
| translation initiation factor 1 | infA | Protein synthesis | BB0169 | 39 | 10 | x | x | x | x | x | x |
| uridine kinase | udk | Purines, pyrimidines, nucleosides, and nucleotides | BB0015 | 39 | 13 | x | x | x | x | x | x |
| excinuclease ABC, subunit B | uvrB | DNA metabolism | BB0836 | 38 | 13 | x |  | x | x | x |  |
| conserved hypothetical protein |  | Hypothetical proteins | BBQ41 | 38 | 14 | x |  | x | x | x |  |
| hydrolase |  | Unknown function | BB0421 | 38 | 12 | x | x | x | x | x | x |
| aldose reductase, putative |  | Unknown function | BB0528 | 38 | 13 | x | x | x | x | x | x |
| rev protein | rev | Unknown function | BBP27 | 38 | 11 |  | x | x | x | x |  |
| hypothetical protein |  |  | BB0044 | 38 | 12 | x | x | x | x | x | x |
| hypothetical protein |  |  | BB0161 | 38 | 18 | x | x | x | x | x | x |
| hypothetical protein |  |  | BB0509 | 38 | 9 | x | x | x | x | x | x |
| hypothetical protein |  |  | BB0569 | 38 | 20 | x |  | x | x | x |  |
| hypothetical protein |  |  | BBA47 | 38 | 10 | x | x | x | x | x | x |
| erpB2 protein | erpB2 | Cell envelope | BBL40 | 37 | 13 | x | x | x | x | x | x |
| conserved hypothetical protein |  | Hypothetical proteins | BB0231 | 37 | 9 | x | x | x | x | x | x |
| zinc protease, putative |  | Protein fate | BB0118 | 37 | 19 | x |  | x | x | x |  |
| protein-export membrane protein | secF | Protein fate | BB0653 | 37 | 9 | x | x | x | x | x | x |
| ribosomal protein L35 | rpmI | Protein synthesis | BB0189 | 37 | 8 | x | x | x | x | x | x |
| hypothetical protein |  |  | BB0346 | 37 | 14 | x | x | x | x | x | x |
| excinuclease ABC, subunit C | uvrC | DNA metabolism | BB0457 | 36 | 21 | x |  | x | x | x |  |
| glucose-6-phosphate 1-dehydrogenase | zwf | Energy metabolism | BB0636 | 36 | 16 | x |  | x | x | x |  |
| conserved hypothetical protein |  | Hypothetical proteins | BBA21 | 36 | 13 | x | x | x | x | x | x |
| lipopolysaccharide biosynthesis-related protein | kdtB | Unknown function | BB0702 | 36 | 12 | x | x | x | x | x | x |
| hypothetical protein |  |  | BB0543 | 36 | 5 | x | x | x | x | x | x |
| hypothetical protein |  |  | BBA39 | 36 | 17 | x | x | x |  | x |  |
| erpB2 protein | erpB2 | Cell envelope | BBP39 | 35 | 13 | x | x | x | x | x | x |
| PTS system, cellobiose-specific IIB component | celA | Transport and binding proteins | BBB06 | 35 | 8 | x | x | x | x | x | x |
| phosphoglycolate phosphatase | gph | Energy metabolism | BB0676 | 34 | 9 | x | x | x | x | x | x |
| conserved hypothetical protein |  | Hypothetical proteins | BB0467 | 34 | 8 | x | x | x | x | x | x |
| conserved hypothetical protein |  | Hypothetical proteins | BBP33 | 34 | 7 | x | x | x | x | x | x |
| protein kinase C1 inhibitor | pkcI | Regulatory functions | BB0379 | 34 | 10 | x | x | x | x | x | x |
| hypothetical protein |  |  | BB0009 | 34 | 17 | x |  | x | x | x |  |
| ribosomal protein S21 | rpsU | Protein synthesis | BB0256 | 33 | 7 | x | x | x | x | x | x |
| hypothetical protein |  |  | BB0852 | 33 | 14 | x | x | x | x | x | x |
| hypothetical protein |  |  | BBA50 | 33 | 19 | x |  | x | x | x |  |
| hypothetical protein |  |  | BBB03 | 33 | 14 | x | x | x | x | x | x |
| protein-glutamate methylesterase | cheB-1 | Cellular processes | BB0415 | 32 | 12 | x | x | x | x | x | x |
| conserved hypothetical protein |  | Hypothetical proteins | BBH29 | 32 | 12 | x |  | x | x | x |  |
| conserved hypothetical protein |  | Hypothetical proteins | BBS36 | 32 | 5 | x | x | x | x | x | x |
| hypothetical protein |  |  | BB0526 | 32 | 12 | x |  | x | x | x |  |
| UDP-N-acetylglucosamine--N-acetylmuramyl-(pentapeptide) pyrophosphoryl-undecaprenol N-acetylglucosamine transferase | murG | Cell envelope | BB0767 | 31 | 17 | x |  | x | x | x |  |
| minD-related ATP-binding protein | ylxH-2 | Cellular processes | BB0361 | 31 | 16 | x | x | x | x | x | x |
| GTP-binding protein | obg | Cellular processes | BB0781 | 31 | 12 | x | x | x | x | x | x |
| conserved hypothetical protein |  | Hypothetical proteins | BB0725 | 31 | 8 | x | x | x | x | x | x |
| conserved hypothetical protein |  | Hypothetical proteins | BB0770 | 31 | 12 | x | x | x | x | x | x |
| conserved hypothetical protein |  | Hypothetical proteins | BB0818 | 31 | 14 | x | x | x | x | x | x |
| cytidine deaminase | cdd | Purines, pyrimidines, nucleosides, and nucleotides | BB0618 | 31 | 14 | x | x | x |  | x |  |
| hypothetical protein |  |  | BB0374 | 31 | 15 | x | x | x | x | x | x |
| erpA protein | erpA | Cell envelope | BBL39 | 30 | 7 | x | x | x | x | x | x |
| plasmid partition protein, putative |  | Cellular processes | BBA20 | 30 | 5 | x | x | x | x | x | x |
| conserved hypothetical protein |  | Hypothetical proteins | BB0225 | 30 | 14 | x |  | x | x | x |  |
| conserved hypothetical protein |  | Hypothetical proteins | BB0682 | 30 | 10 | x |  | x | x | x |  |
| conserved hypothetical protein |  | Hypothetical proteins | BBJ16 | 30 | 17 | x | x | x |  | x |  |
| response regulatory protein | rrp-1 | Regulatory functions | BB0419 | 30 | 7 | x | x | x | x | x | x |
| ABC transporter, ATP-binding protein |  | Transport and binding proteins | BB0573 | 30 | 8 | x | x | x | x | x | x |
| ORFZ01931 |  |  | ORFZ01931 | 30 | 14 | x |  | x |  | x |  |
| pantothenate metabolism flavoprotein | dfp | Biosynthesis of cofactors, prosthetic groups, and carriers | BB0812 | 29 | 17 | x | x | x | x | x | x |
| antigen, P35, putative |  | Cell envelope | BBI36 | 29 | 11 | x | x | x |  | x |  |
| plasmid partition protein, putative |  | Cellular processes | BBR33 | 29 | 10 | x | x | x | x | x | x |
| replicative DNA helicase | dnaB | DNA metabolism | BB0111 | 29 | 12 | x | x | x | x | x | x |
| conserved hypothetical protein |  | Hypothetical proteins | BB0268 | 29 | 8 | x | x | x | x | x | x |
| conserved hypothetical protein |  | Hypothetical proteins | BBU06 | 29 | 16 |  |  | x |  | x |  |
| sensory transduction histidine kinase/response regulator |  | Regulatory functions | BB0420 | 29 | 19 | x | x | x | x | x | x |
| thymidylate synthase-complementing protein | thy1 | Unknown function | BBA76 | 29 | 13 | x |  | x | x | x |  |
| hypothetical protein |  |  | BB0367 | 29 | 12 | x | x | x | x | x | x |
| purine-binding chemotaxis protein | cheW-2 | Cellular processes | BB0565 | 28 | 7 | x | x | x | x | x | x |
| GTP-binding protein | era | Cellular processes | BB0660 | 28 | 12 | x |  | x | x | x |  |
| conserved hypothetical protein |  | Hypothetical proteins | BB0782 | 28 | 7 | x | x | x | x | x | x |
| serine/threonine kinase, putative |  | Protein fate | BB0648 | 28 | 15 | x | x | x | x | x | x |
| dimethyladenosine transferase | ksgA | Protein synthesis | BB0590 | 28 | 13 | x | x | x | x | x | x |
| hypothetical protein |  |  | BB0082 | 28 | 13 | x |  | x | x | x |  |
| NH(3)-dependent NAD+ synthetase |  | Biosynthesis of cofactors, prosthetic groups, and carriers | BB0522 | 27 | 10 | x | x | x | x | x | x |
| phosphomannomutase | cpsG | Energy metabolism | BB0835 | 27 | 18 | x |  | x | x | x |  |
| conserved hypothetical protein |  | Hypothetical proteins | BB0247 | 27 | 9 | x | x | x | x | x | x |
| conserved hypothetical protein |  | Hypothetical proteins | BB0449 | 27 | 7 | x | x | x | x | x | x |
| plasmid partition protein, putative |  | Hypothetical proteins | BBB12 | 27 | 10 | x | x | x | x | x | x |
| conserved hypothetical protein |  | Hypothetical proteins | BBC03 | 27 | 14 | x |  | x |  | x |  |
| conserved hypothetical protein |  | Hypothetical proteins | BBO33 | 27 | 12 | x | x | x | x | x | x |
| conserved hypothetical protein |  | Hypothetical proteins | BBR34 | 27 | 9 | x |  | x | x | x |  |
| conserved hypothetical protein |  | Hypothetical proteins | BBS29 | 27 | 9 | x | x | x | x | x | x |
| conserved hypothetical protein |  | Hypothetical proteins | BBS37 | 27 | 14 | x | x | x | x | x | x |
| hypothetical protein |  |  | BB0418 | 27 | 9 | x | x | x | x | x | x |
| lipoprotein |  | Cell envelope | BBA62 | 26 | 8 | x | x | x | x | x | x |
| flagellar motor switch protein | fliG-1 | Cellular processes | BB0221 | 26 | 13 | x | x | x | x | x | x |
| flagellar basal-body rod protein | flgC | Cellular processes | BB0293 | 26 | 9 | x |  | x | x | x |  |
| plasmid partition protein, putative |  | Cellular processes | BBH28 | 26 | 9 | x |  | x | x | x |  |
| conserved hypothetical integral membrane protein |  | Hypothetical proteins | BB0616 | 26 | 10 | x | x | x | x | x | x |
| conserved hypothetical protein, |  | Hypothetical proteins | BBI22 | 26 | 7 | x | x | x | x | x | x |
| ribosomal protein L32 | rpmF | Protein synthesis | BB0703 | 26 | 3 | x | x | x | x | x | x |
| hypothetical protein |  |  | BB0106 | 26 | 12 | x | x | x | x | x | x |
| hypothetical protein |  |  | BB0353 | 26 | 12 | x | x | x | x | x | x |
| methylenetetrahydrofolate dehydrogenase | folD | Biosynthesis of cofactors, prosthetic groups, and carriers | BB0026 | 25 | 10 | x |  | x | x | x |  |
| acyl carrier protein |  | Fatty acid and phospholipid metabolism | BB0704 | 25 | 3 | x | x | x | x | x | x |
| conserved hypothetical protein |  | Hypothetical proteins | BB0731 | 25 | 13 | x | x | x | x | x | x |
| conserved hypothetical protein |  | Hypothetical proteins | BB0799 | 25 | 7 | x |  | x | x | x |  |
| exodeoxyribonuclease V, alpha chain | recD | DNA metabolism | BB0632 | 24 | 11 | x | x | x | x | x | x |
| conserved hypothetical protein |  | Hypothetical proteins | BB0099 | 24 | 9 | x | x | x | x | x | x |
| conserved hypothetical protein |  | Hypothetical proteins | BBA13 | 24 | 9 | x | x | x | x | x | x |
| hypothetical protein |  |  | BB0400 | 24 | 16 | x | x | x |  | x |  |
| hypothetical protein |  |  | BB0816 | 24 | 9 | x | x | x | x | x | x |
| plasmid partition protein, putative |  | Cellular processes | BBG08 | 23 | 12 | x | x | x | x | x | x |
| conserved hypothetical protein |  | Hypothetical proteins | BBA11 | 23 | 13 | x | x | x | x | x | x |
| conserved hypothetical protein |  | Hypothetical proteins | BBL34 | 23 | 14 |  |  | x |  | x |  |
| thymidylate kinase | tmk | Purines, pyrimidines, nucleosides, and nucleotides | BB0793 | 23 | 11 | x |  | x | x | x |  |
| hypothetical protein |  |  | BB0139 | 23 | 5 | x | x | x | x | x | x |
| hypothetical protein |  |  | BB0165 | 23 | 10 | x | x | x | x | x | x |
| hypothetical protein |  |  | BBI38 | 23 | 10 | x | x | x |  | x |  |
| hypothetical protein |  |  | BBJ36 | 23 | 16 | x | x | x |  | x |  |
| hemolysin | tlyC | Cellular processes | BB0059 | 22 | 11 | x | x | x | x | x | x |
| minD-related ATP-binding protein | ylxH-1 | Cellular processes | BB0269 | 22 | 14 | x | x | x | x | x | x |
| sialoglycoprotease | gcp | Protein fate | BB0769 | 22 | 14 | x | x | x | x | x | x |
| alanine racemase | alr | Cell envelope | BB0160 | 21 | 11 | x | x | x | x | x | x |
| penicillin-binding protein | pbp-2 | Cell envelope | BB0718 | 21 | 10 | x |  | x | x | x |  |
| plasmid partition protein, putative |  | Cellular processes | BBI21 | 21 | 11 | x | x | x |  | x |  |
| plasmid partition protein, putative |  | Cellular processes | BBN32 | 21 | 10 | x |  | x |  | x |  |
| spoU protein | spoU | Protein synthesis | BB0052 | 21 | 8 | x | x | x | x | x | x |
| hypothetical protein |  |  | BB0309 | 21 | 9 | x |  | x | x | x |  |
| hypothetical protein |  |  | BB0398 | 21 | 10 | x |  | x | x | x |  |
| hypothetical protein |  |  | BBA46 | 21 | 8 | x | x | x | x | x | x |
| rod shape-determining protein | mreC | Cell envelope | BB0716 | 20 | 7 | x |  | x | x | x |  |
| transcription-repair coupling factor | mfd | DNA metabolism | BB0623 | 20 | 13 | x | x | x | x | x | x |
| DNA mismatch repair protein | mutS | DNA metabolism | BB0797 | 20 | 13 |  |  | x | x | x |  |
| conserved hypothetical GTP-binding protein |  | Hypothetical proteins | BB0643 | 20 | 11 | x | x | x | x | x | x |
| conserved hypothetical protein |  | Hypothetical proteins | BBK40 | 20 | 12 | x |  | x |  | x |  |
| ribosomal protein S14 | rpsN | Protein synthesis | BB0491 | 20 | 2 | x | x | x | x | x | x |
| PTS system, glucose-specific IIBC component | ptsG | Transport and binding proteins | BB0645 | 20 | 9 | x |  | x | x | x |  |
| smg protein |  | Unknown function | BB0297 | 20 | 9 | x | x | x | x | x | x |
| hypothetical protein |  |  | BB0162 | 20 | 4 | x | x | x | x | x | x |
| hypothetical protein |  |  | BB0405 | 20 | 5 | x | x | x | x | x | x |
| hypothetical protein |  |  | BBJ23 | 20 | 11 |  | x | x |  | x |  |
| DNA ligase | lig | DNA metabolism | BB0552 | 19 | 13 | x | x | x | x | x | x |
| conserved hypothetical protein |  | Hypothetical proteins | BB0505 | 19 | 9 | x | x | x | x | x | x |
| conserved hypothetical integral membrane protein |  | Hypothetical proteins | BB0843 | 19 | 4 | x |  | x | x | x |  |
| hypothetical protein, paralogous family 85 |  | Hypothetical proteins | BBD15 | 19 | 6 | x |  | x | x | x |  |
| pseudouridylate synthase I | hisT | Protein synthesis | BB0012 | 19 | 6 | x | x | x | x | x | x |
| hypothetical protein |  |  | BB0083 | 19 | 6 | x | x | x | x | x | x |
| antigen, P35 |  | Cell envelope | BBA64 | 18 | 9 |  | x |  | x | x |  |
| DNA mismatch repair protein, putative |  | DNA metabolism | BB0098 | 18 | 14 |  |  | x | x | x |  |
| conserved hypothetical protein |  | Hypothetical proteins | BB0377 | 18 | 8 | x |  | x | x | x |  |
| glutamate transporter | gltP | Transport and binding proteins | BB0729 | 18 | 6 | x |  | x | x | x |  |
| hypothetical protein |  |  | BB0134 | 18 | 10 | x |  | x | x | x |  |
| hypothetical protein |  |  | BB0163 | 18 | 11 | x |  | x | x | x |  |
| hypothetical protein |  |  | BB0674 | 18 | 9 | x | x | x | x | x | x |
| hypothetical protein |  |  | BB0832 | 18 | 10 | x |  | x | x | x |  |
| primosomal protein N' | priA | DNA metabolism | BB0014 | 17 | 11 | x |  | x | x | x |  |
| exodeoxyribonuclease III | exoA | DNA metabolism | BB0534 | 17 | 8 | x | x | x | x | x | x |
| phosphate acetyltransferase | pta | Energy metabolism | BB0589 | 17 | 8 |  | x | x | x | x |  |
| conserved hypothetical protein |  | Hypothetical proteins | BBG09 | 17 | 10 | x |  | x |  | x |  |
| hypothetical protein |  |  | BB0066 | 17 | 11 | x |  | x | x | x |  |
| hypothetical protein |  |  | BB0071 | 17 | 8 | x |  | x | x | x |  |
| hypothetical protein |  |  | BB0663 | 17 | 8 | x | x |  | x | x |  |
| hypothetical protein |  |  | BB0806 | 17 | 6 | x | x | x | x | x | x |
| hypothetical protein |  |  | BBH09 | 17 | 4 | x | x | x | x | x | x |
| ORFZ01935 |  |  | ORFZ01935 | 17 | 6 |  | x | x | x | x |  |
| penicillin-binding protein | pbp-1 | Cell envelope | BB0136 | 16 | 9 | x |  | x | x | x |  |
| antigen, S1 |  | Cell envelope | BBA05 | 16 | 11 | x | x | x | x | x | x |
| plasmid partition protein, putative |  | Cellular processes | BBJ17 | 16 | 9 | x |  | x |  | x |  |
| plasmid partition protein, putative |  | Cellular processes | BBQ40 | 16 | 9 | x | x | x |  | x |  |
| thioredoxin reductase | trxB | Energy metabolism | BB0515 | 16 | 7 | x | x | x | x | x | x |
| conserved hypothetical protein |  | Hypothetical proteins | BB0819 | 16 | 3 | x | x | x | x | x | x |
| conserved hypothetical protein |  | Hypothetical proteins | BBO34 | 16 | 10 | x |  | x | x | x |  |
| conserved hypothetical protein |  | Hypothetical proteins | BBQ42 | 16 | 8 | x | x | x | x | x | x |
| ABC transporter, ATP-binding protein |  | Transport and binding proteins | BB0080 | 16 | 5 | x |  | x | x | x |  |
| hypothetical protein |  |  | BB0654 | 16 | 8 | x |  |  | x | x |  |
| hypothetical protein |  |  | BBA38 | 16 | 8 |  | x | x | x | x |  |
| thiophene and furan oxidation protein | thdF | Cellular processes | BB0179 | 15 | 10 | x |  | x | x | x |  |
| phnP protein | phnP | Central intermediary metabolism | BB0533 | 15 | 6 |  | x | x | x | x |  |
| conserved hypothetical protein |  | Hypothetical proteins | BB0306 | 15 | 9 | x |  | x | x | x |  |
| conserved hypothetical protein |  | Hypothetical proteins | BBA09 | 15 | 7 | x | x | x |  | x |  |
| conserved hypothetical protein |  | Hypothetical proteins | BBB11 | 15 | 6 | x | x | x | x | x | x |
| hypothetical protein |  |  | BB0089 | 15 | 9 | x |  | x | x | x |  |
| hypothetical protein |  |  | BB0170 | 15 | 5 | x | x | x | x | x | x |
| hypothetical protein |  |  | BB0432 | 15 | 10 |  |  | x | x | x |  |
| B_burgdorferi_B31:21974_22252 |  |  | NA | 15 | 7 | x |  | x | x | x |  |
| erpB2 protein | erpB2 | Cell envelope | BBN39 | 14 | 9 | x | x | x |  | x |  |
| plasmid partition protein, putative |  | Cellular processes | BBO32 | 14 | 9 | x | x | x | x | x | x |
| V-type ATPase, subunit D | atpD | Energy metabolism | BB0092 | 14 | 7 | x |  | x | x | x |  |
| 3-hydroxy-3-methylglutaryl-CoA reductase | mvaA | Energy metabolism | BB0685 | 14 | 7 | x |  | x | x | x |  |
| conserved hypothetical protein |  | Hypothetical proteins | BBA43 | 14 | 6 | x | x | x |  | x |  |
| conserved hypothetical protein |  | Hypothetical proteins | BBL35 | 14 | 8 | x |  | x | x | x |  |
| signal peptidase I | lepB-2 | Protein fate | BB0031 | 14 | 10 |  | x | x | x | x |  |
| cytidylate kinase | cmk | Purines, pyrimidines, nucleosides, and nucleotides | BB0128 | 14 | 8 | x | x | x | x | x | x |
| PTS system, cellobiose-specific IIC component | celB | Transport and binding proteins | BBB04 | 14 | 4 | x | x | x | x | x | x |
| hypothetical protein |  |  | BB0260 | 14 | 2 | x | x | x | x | x | x |
| hypothetical protein |  |  | BB0577 | 14 | 8 | x | x |  | x | x |  |
| erpA protein | erpA | Cell envelope | BBP38 | 13 | 10 | x | x | x | x | x | x |
| plasmid partition protein, putative |  | Cellular processes | BBS35 | 13 | 5 | x | x | x | x |  |  |
| conserved hypothetical protein |  | Hypothetical proteins | BBB13 | 13 | 7 | x | x | x | x | x | x |
| hypothetical protein |  | Hypothetical proteins | BBD10 | 13 | 7 | x | x | x | x | x | x |
| conserved hypothetical protein |  | Hypothetical proteins | BBP34 | 13 | 11 | x | x | x | x | x | x |
| conserved hypothetical protein |  | Hypothetical proteins | BBR27 | 13 | 5 | x | x | x | x | x | x |
| sensory transduction histidine kinase, putative |  | Regulatory functions | BB0764 | 13 | 6 | x |  | x | x | x |  |
| hypothetical protein |  |  | BB0003 | 13 | 5 | x | x | x | x | x | x |
| hypothetical protein |  |  | BB0352 | 13 | 4 | x | x | x | x | x | x |
| hypothetical protein |  |  | BB0790 | 13 | 7 | x |  |  | x | x |  |
| hypothetical protein |  |  | BB0838 | 13 | 10 | x | x | x | x | x | x |
| hypothetical protein |  |  | BBA55 | 13 | 8 | x | x | x |  | x |  |
| hypothetical protein |  |  | BBB09 | 13 | 9 | x |  | x | x | x |  |
| B_burgdorferi_B31:501012_501212 |  |  | NA | 13 | 3 | x | x | x | x | x | x |
| B_burgdorferi_B31:4099_1 |  |  | NA | 13 | 9 | x | x | x | x | x | x |
| femA protein | femA | Cell envelope | BB0586 | 12 | 7 |  | x | x | x | x |  |
| flagellar protein | flbD | Cellular processes | BB0282 | 12 | 7 |  |  | x | x | x |  |
| endonuclease III | nth | DNA metabolism | BB0745 | 12 | 7 |  |  | x | x | x |  |
| beta-N-acetylhexosaminidase, putative |  | Energy metabolism | BB0002 | 12 | 6 | x |  | x | x | x |  |
| conserved hypothetical protein |  | Hypothetical proteins | BB0175 | 12 | 6 | x | x | x | x | x | x |
| conserved hypothetical protein |  | Hypothetical proteins | BBN34 | 12 | 10 |  | x | x | x | x |  |
| conserved hypothetical protein |  | Hypothetical proteins | BBQ34 | 12 | 6 | x | x | x | x | x | x |
| conserved hypothetical protein |  | Hypothetical proteins | BBS34 | 12 | 3 | x | x | x | x | x | x |
| apolipoprotein N-acyltransferase, putative |  | Protein fate | BB0237 | 12 | 8 |  |  | x | x | x |  |
| carbon storage regulator | csrA | Regulatory functions | BB0184 | 12 | 4 |  | x | x | x | x |  |
| histidine phosphokinase/phophatase, putative |  | Regulatory functions | BB0737 | 12 | 6 | x |  | x | x | x |  |
| oligopeptide ABC transporter, permease protein | oppB-1 | Transport and binding proteins | BB0332 | 12 | 3 | x | x | x | x | x | x |
| hypothetical protein |  |  | BB0075 | 12 | 7 | x | x | x | x | x | x |
| hypothetical protein |  |  | BB0665 | 12 | 5 | x |  |  | x |  |  |
| hypothetical protein |  |  | BB0794 | 12 | 9 |  | x |  | x | x |  |
| outer membrane protein | tpn50 | Cell envelope | BB0167 | 11 | 9 | x |  |  | x | x |  |
| immunogenic protein P37 |  | Cell envelope | BBK50 | 11 | 6 | x |  | x |  | x |  |
| purine-binding chemotaxis protein | cheW-1 | Cellular processes | BB0312 | 11 | 5 |  | x |  | x | x |  |
| plasmid partition protein, putative |  | Cellular processes | BBP32 | 11 | 9 | x | x | x | x | x | x |
| exodeoxyribonuclease V, beta chain | recB | DNA metabolism | BB0633 | 11 | 11 |  | x | x | x | x |  |
| exodeoxyribonuclease V, gamma chain | recC | DNA metabolism | BB0634 | 11 | 9 |  | x | x |  | x |  |
| exonuclease SbcC | sbcC | DNA metabolism | BB0830 | 11 | 8 | x |  | x |  | x |  |
| conserved hypothetical protein |  | Hypothetical proteins | BBF20 | 11 | 6 | x |  | x |  | x |  |
| conserved hypothetical protein |  | Hypothetical proteins | BBP31 | 11 | 3 | x |  | x | x | x |  |
| ribosomal protein L36 | rpmJ | Protein synthesis | BB0499 | 11 | 1 | x |  | x | x | x |  |
| Na+/H+ antiporter | napA | Transport and binding proteins | BB0447 | 11 | 5 | x | x | x | x | x | x |
| P26 |  | Unknown function | BB0336 | 11 | 6 | x |  |  | x | x |  |
| hypothetical protein |  |  | BB0209 | 11 | 8 | x | x | x | x | x | x |
| hypothetical protein |  |  | BB0245 | 11 | 6 | x |  | x | x | x |  |
| hypothetical protein |  |  | BB0614 | 11 | 5 | x | x | x | x | x | x |
| hypothetical protein |  |  | BB0675 | 11 | 6 | x |  | x | x | x |  |
| hypothetical protein |  |  | BBA48 | 11 | 4 | x | x | x | x | x | x |
| hypothetical protein |  |  | BBJ08 | 11 | 8 | x |  | x |  | x |  |
| hypothetical protein |  |  | BBJ27 | 11 | 6 | x |  | x |  | x |  |
| UDP-N-acetylmuramoylalanine--D-glutamate ligase | murD | Cell envelope | BB0585 | 10 | 5 | x |  | x | x | x |  |
| flagellar protein, putative |  | Cellular processes | BB0180 | 10 | 4 | x | x | x | x | x | x |
| hemolysin, putative |  | Cellular processes | BB0202 | 10 | 7 | x |  | x | x | x |  |
| flagellar biosynthesis protein | fliZ | Cellular processes | BB0276 | 10 | 6 | x |  | x | x | x |  |
| chemotaxis protein methyltransferase | cheR-2 | Cellular processes | BB0414 | 10 | 4 | x | x | x | x | x | x |
| phosphomevalonate kinase, putative |  | Fatty acid and phospholipid metabolism | BB0687 | 10 | 4 |  | x | x | x | x |  |
| conserved hypothetical protein |  | Hypothetical proteins | BB0185 | 10 | 3 | x | x | x | x | x | x |
| conserved hypothetical protein |  | Hypothetical proteins | BBH41 | 10 | 2 | x | x | x |  | x |  |
| conserved hypothetical protein |  | Hypothetical proteins | BBK24 | 10 | 8 | x |  | x |  | x |  |
| glycine betaine, L-proline ABC transporter, permease protein | proW | Transport and binding proteins | BB0145 | 10 | 2 | x | x | x | x | x | x |
| Mg2+ transport protein | mgtE | Transport and binding proteins | BB0380 | 10 | 8 |  |  | x | x | x |  |
| ABC transporter, ATP-binding protein |  | Transport and binding proteins | BBJ26 | 10 | 6 |  |  | x | x | x |  |
| hypothetical protein |  |  | BB0007 | 10 | 6 | x | x |  | x | x |  |
| hypothetical protein |  |  | BB0265 | 10 | 4 | x |  | x | x | x |  |
| hypothetical protein |  |  | BB0459 | 10 | 6 |  | x | x | x | x |  |
| hypothetical protein |  |  | BB0521 | 10 | 5 |  |  | x | x | x |  |
| hypothetical protein |  |  | BB0617 | 10 | 6 | x |  |  | x | x |  |
| fibronectin/fibrinogen-binding protein, putative |  | Cell envelope | BB0347 | 9 | 7 | x | x | x | x | x | x |
| lipoprotein |  | Cell envelope | BBA36 | 9 | 2 | x | x | x |  | x |  |
| lipoprotein |  | Cell envelope | BBA59 | 9 | 3 | x | x |  | x | x |  |
| lipoprotein |  | Cell envelope | BBP28 | 9 | 5 |  | x | x | x | x |  |
| conserved hypothetical protein |  | Hypothetical proteins | BB0183 | 9 | 3 | x | x | x | x | x | x |
| conserved hypothetical protein |  | Hypothetical proteins | BB0471 | 9 | 3 | x |  | x | x | x |  |
| hypothetical protein |  | Hypothetical proteins | BBD18 | 9 | 4 | x |  | x | x |  |  |
| chaperonin, putative |  | Protein fate | BB0602 | 9 | 5 |  |  | x | x | x |  |
| L-lactate permease | lctP | Transport and binding proteins | BB0604 | 9 | 2 |  |  | x | x | x |  |
| hypothetical protein |  |  | BB0542 | 9 | 3 | x |  |  | x | x |  |
| hypothetical protein |  |  | BB0600 | 9 | 6 | x |  |  | x | x |  |
| hypothetical protein |  |  | BB0701 | 9 | 5 | x |  | x | x | x |  |
| hypothetical protein |  |  | BBR01 | 9 | 2 | x |  | x | x | x |  |
| antigen, P35, putative |  | Cell envelope | BBA73 | 8 | 6 |  | x |  | x | x |  |
| DNA helicase | uvrD | DNA metabolism | BB0344 | 8 | 4 | x |  | x | x | x |  |
| mevalonate pyrophosphate decarboxylase |  | Fatty acid and phospholipid metabolism | BB0686 | 8 | 5 | x |  |  | x | x |  |
| conserved hypothetical protein |  | Hypothetical proteins | BBD11 | 8 | 6 | x |  | x |  | x |  |
| conserved hypothetical protein |  | Hypothetical proteins | BBF23 | 8 | 4 | x |  | x |  | x |  |
| conserved hypothetical protein |  | Hypothetical proteins | BBP29 | 8 | 5 |  | x |  | x | x |  |
| prolipoprotein diacylglyceryl transferase | lgt | Protein fate | BB0362 | 8 | 5 |  | x | x | x | x |  |
| tRNA (guanine-N1)-methyltransferase | trmD | Protein synthesis | BB0698 | 8 | 6 |  | x | x | x | x |  |
| uridylate kinase | smbA | Purines, pyrimidines, nucleosides, and nucleotides | BB0571 | 8 | 4 |  |  |  | x | x |  |
| HemK family methylase, putative |  | Unknown function | BB0197 | 8 | 4 | x |  | x | x |  |  |
| periplasmic protein |  | Unknown function | BB0363 | 8 | 5 |  | x | x | x | x |  |
| hypothetical protein |  |  | BB0013 | 8 | 6 | x | x | x |  | x |  |
| hypothetical protein |  |  | BB0085 | 8 | 6 |  |  | x |  | x |  |
| hypothetical protein |  |  | BB0171 | 8 | 4 | x |  | x | x | x |  |
| hypothetical protein |  |  | BB0199 | 8 | 6 | x |  |  | x | x |  |
| hypothetical protein |  |  | BB0535 | 8 | 3 | x | x |  | x | x |  |
| hypothetical protein |  |  | BB0562 | 8 | 3 | x | x | x | x | x | x |
| hypothetical protein |  |  | BB0765 | 8 | 6 | x |  | x | x | x |  |
| hypothetical protein |  |  | BBK47 | 8 | 6 |  | x | x |  | x |  |
| lipoprotein | lp | Cell envelope | BBO28 | 7 | 4 |  |  | x | x | x |  |
| cell division protein, putative |  | Cellular processes | BB0257 | 7 | 7 | x |  | x | x | x |  |
| uracil DNA glycosylase | ung | DNA metabolism | BB0053 | 7 | 7 | x |  |  | x | x |  |
| conserved hypothetical protein |  | Hypothetical proteins | BB0008 | 7 | 4 | x |  | x | x | x |  |
| conserved hypothetical protein |  | Hypothetical proteins | BBC02 | 7 | 4 | x |  | x |  | x |  |
| plasmid partition protein, putative |  | Hypothetical proteins | BBF24 | 7 | 4 | x |  | x |  | x |  |
| conserved hypothetical protein |  | Hypothetical proteins | BBL27 | 7 | 2 |  | x |  | x | x |  |
| polynucleotide adenylyltransferase | papS | Transcription | BB0706 | 7 | 6 |  |  | x | x | x |  |
| RNA polymerase sigma factor | rpoS | Transcription | BB0771 | 7 | 5 |  | x | x | x | x |  |
| carotenoid biosynthesis protein, putative |  | Unknown function | BB0684 | 7 | 4 | x |  | x | x | x |  |
| rev protein | rev | Unknown function | BBM27 | 7 | 5 |  | x |  | x | x |  |
| hypothetical protein |  |  | BB0077 | 7 | 4 | x |  | x | x | x |  |
| hypothetical protein |  |  | BB0531 | 7 | 5 | x |  | x |  |  |  |
| hypothetical protein |  |  | BB0556 | 7 | 3 | x |  | x | x | x |  |
| hypothetical protein |  |  | BB0707 | 7 | 7 | x | x | x | x | x | x |
| hypothetical protein |  |  | BB0722 | 7 | 4 |  | x |  | x | x |  |
| hypothetical protein |  |  | BB0756 | 7 | 6 | x |  | x |  | x |  |
| hypothetical protein |  |  | BBB08 | 7 | 3 |  |  | x | x | x |  |
| rod shape-determining protein | mreB-2 | Cell envelope | BB0719 | 6 | 2 | x |  |  | x | x |  |
| antigen, S2 |  | Cell envelope | BBA04 | 6 | 5 |  | x | x |  | x |  |
| erpM protein | erpM | Cell envelope | BBO40 | 6 | 1 | x | x | x | x | x | x |
| chemotaxis protein methyltransferase | cheR-1 | Cellular processes | BB0040 | 6 | 4 |  | x |  | x | x |  |
| endonuclease precursor | nucA | DNA metabolism | BB0411 | 6 | 4 |  |  | x | x | x |  |
| conserved hypothetical protein |  | Hypothetical proteins | BB0244 | 6 | 3 |  |  | x | x | x |  |
| conserved hypothetical protein |  | Hypothetical proteins | BB0784 | 6 | 2 | x |  | x | x | x |  |
| conserved hypothetical protein |  | Hypothetical proteins | BB0788 | 6 | 4 | x |  |  | x | x |  |
| conserved hypothetical integral membrane protein |  | Hypothetical proteins | BB0807 | 6 | 3 |  |  |  | x | x |  |
| conserved hypothetical protein |  | Hypothetical proteins | BBO27 | 6 | 6 |  |  | x |  | x |  |
| conserved hypothetical protein |  | Hypothetical proteins | BBU11 | 6 | 4 |  |  | x |  | x |  |
| ATP-dependent Clp protease, subunit A | clpA | Protein fate | BB0369 | 6 | 3 | x |  | x | x | x |  |
| N-utilization substance protein B | nusB | Transcription | BB0107 | 6 | 3 | x |  |  | x | x |  |
| GTP-binding protein |  | Unknown function | BB0508 | 6 | 3 |  |  | x | x | x |  |
| rev protein | rev | Unknown function | BBC10 | 6 | 4 |  |  |  |  | x |  |
| hypothetical protein |  |  | BB0356 | 6 | 3 |  |  | x | x | x |  |
| hypothetical protein |  |  | BB0808 | 6 | 4 |  |  | x | x | x |  |
| hypothetical protein |  |  | BBJ28 | 6 | 5 |  |  | x |  | x |  |
| hypothetical protein |  |  | BBK49 | 6 | 3 | x |  | x |  | x |  |
| B_burgdorferi_B31:540776_541234 |  |  | NA | 6 | 2 | x |  | x | x |  |  |
| B_burgdorferi_B31:4099_2 |  |  | NA | 6 | 4 | x |  | x | x | x | x |
| undecaprenyl diphosphate synthase |  | Biosynthesis of cofactors, prosthetic groups, and carriers | BB0120 | 5 | 4 |  |  |  | x | x |  |
| UDP-N-acetylmuramate dehydrogenase | murB | Cell envelope | BB0598 | 5 | 4 |  |  |  | x |  |  |
| immunogenic protein P35 |  | Cell envelope | BBK32 | 5 | 3 | x |  | x | x | x |  |
| outer surface protein F | ospF | Cell envelope | BBR42 | 5 | 2 | x |  | x | x | x |  |
| plasmid partition protein, putative |  | Cellular processes | BBK21 | 5 | 2 |  | x | x |  | x |  |
| Holliday junction DNA helicase | ruvB | DNA metabolism | BB0022 | 5 | 4 |  |  | x | x | x |  |
| Holliday junction DNA helicase | ruvA | DNA metabolism | BB0023 | 5 | 4 |  |  | x | x | x |  |
| DNA primase | dnaG | DNA metabolism | BB0710 | 5 | 4 | x |  |  |  | x |  |
| 1-acyl-sn-glycerol-3-phosphate acyltransferase | plsC | Fatty acid and phospholipid metabolism | BB0037 | 5 | 3 | x |  |  | x | x |  |
| conserved hypothetical protein |  | Hypothetical proteins | BB0262 | 5 | 3 |  |  | x | x | x |  |
| conserved hypothetical protein |  | Hypothetical proteins | BB0427 | 5 | 4 |  |  |  | x |  |  |
| conserved hypothetical protein |  | Hypothetical proteins | BB0761 | 5 | 4 | x |  |  | x | x |  |
| conserved hypothetical protein |  | Hypothetical proteins | BBA19 | 5 | 4 |  |  |  | x | x |  |
| conserved hypothetical protein |  | Hypothetical proteins | BBG31 | 5 | 4 | x | x | x | x |  |  |
| conserved hypothetical protein |  | Hypothetical proteins | BBJ19 | 5 | 4 |  |  | x |  | x |  |
| conserved hypothetical protein |  | Hypothetical proteins | BBM34 | 5 | 4 |  | x | x | x | x |  |
| conserved hypothetical protein |  | Hypothetical proteins | BBR22 | 5 | 1 |  |  | x | x | x |  |
| preprotein translocase subunit | secY | Protein fate | BB0498 | 5 | 4 |  |  | x | x | x |  |
| rRNA methylase | yacO | Protein synthesis | BB0516 | 5 | 4 |  |  |  | x | x |  |
| thymidine kinase | tdk | Purines, pyrimidines, nucleosides, and nucleotides | BB0791 | 5 | 5 | x |  | x |  | x |  |
| adenine deaminase | adeC | Purines, pyrimidines, nucleosides, and nucleotides | BBK17 | 5 | 3 | x | x |  |  | x |  |
| hypothetical protein |  |  | BB0079 | 5 | 3 |  |  | x | x | x |  |
| hypothetical protein |  |  | BB0315 | 5 | 3 | x |  |  | x | x |  |
| hypothetical protein |  |  | BB0373 | 5 | 2 |  |  | x | x | x |  |
| hypothetical protein |  |  | BB0406 | 5 | 4 |  |  |  | x | x |  |
| hypothetical protein |  |  | BB0465 | 5 | 3 | x |  |  | x | x |  |
| hypothetical protein |  |  | BB0609 | 5 | 1 | x |  |  | x | x |  |
| hypothetical protein |  |  | BBJ24 | 5 | 3 | x |  | x | x |  |  |
| ORFZ01792 |  |  | ORFZ01792 | 5 | 4 | x |  | x |  | x |  |
| ORFZ01811 |  |  | ORFZ01811 | 5 | 3 |  |  |  | x | x |  |
| B_burgdorferi_B31:382974_383078 |  |  | NA | 5 | 2 | x | x | x |  | x |  |
| B_burgdorferi_B31:32498_33220 |  |  | NA | 5 | 3 | x |  | x |  | x |  |
| lipoprotein | lp | Cell envelope | BBM28 | 4 | 2 |  |  |  | x | x |  |
| flagellar protein | flbA | Cellular processes | BB0287 | 4 | 3 |  |  | x | x | x |  |
| colicin V production protein, putative |  | Cellular processes | BB0766 | 4 | 1 | x |  |  | x | x |  |
| plasmid partition protein, putative |  | Cellular processes | BBL32 | 4 | 2 |  |  | x |  | x |  |
| plasmid partition protein, putative |  | Cellular processes | BBU05 | 4 | 4 |  |  |  |  | x |  |
| conserved hypothetical protein |  | Hypothetical proteins | BB0081 | 4 | 4 |  | x | x |  | x |  |
| conserved hypothetical protein |  | Hypothetical proteins | BB0468 | 4 | 1 |  |  | x | x | x |  |
| conserved hypothetical protein |  | Hypothetical proteins | BB0527 | 4 | 1 |  |  |  | x | x |  |
| conserved hypothetical integral membrane protein |  | Hypothetical proteins | BB0539 | 4 | 2 | x |  |  | x | x |  |
| hypothetical protein |  | Hypothetical proteins | BBD09 | 4 | 4 |  |  |  | x | x |  |
| hypothetical protein |  | Hypothetical proteins | BBD22 | 4 | 3 |  |  |  | x | x |  |
| conserved hypothetical protein |  | Hypothetical proteins | BBG29 | 4 | 3 | x |  | x |  | x |  |
| conserved hypothetical protein |  | Hypothetical proteins | BBN27 | 4 | 3 |  | x | x |  | x |  |
| conserved hypothetical protein |  | Hypothetical proteins | BBS31 | 4 | 4 |  |  |  | x |  |  |
| signal peptidase I | lepB-1 | Protein fate | BB0030 | 4 | 2 |  |  | x | x | x |  |
| 5'-methylthioadenosine/S-adenosylhomocysteine nucleosidase, putative | pfs-I | Purines, pyrimidines, nucleosides, and nucleotides | BBI06 | 4 | 2 |  |  |  | x | x |  |
| phosphate ABC transporter, permease protein | pstA | Transport and binding proteins | BB0217 | 4 | 2 |  |  |  | x | x |  |
| methylgalactoside ABC transporter, ATP-binding protein | mglA | Transport and binding proteins | BB0318 | 4 | 3 |  | x |  |  | x |  |
| hypothetical protein |  |  | BB0130 | 4 | 3 | x |  |  | x | x |  |
| hypothetical protein |  |  | BB0193 | 4 | 4 |  |  | x | x |  |  |
| hypothetical protein |  |  | BB0213 | 4 | 3 |  |  |  | x | x |  |
| hypothetical protein |  |  | BB0340 | 4 | 2 |  |  |  | x | x |  |
| hypothetical protein |  |  | BB0403 | 4 | 3 |  |  | x | x |  |  |
| hypothetical protein |  |  | BB0464 | 4 | 2 |  |  |  | x | x |  |
| hypothetical protein |  |  | BB0566 | 4 | 2 |  |  | x | x | x |  |
| hypothetical protein |  |  | BB0773 | 4 | 3 | x |  | x | x |  |  |
| hypothetical protein |  |  | BB0825 | 4 | 1 | x |  | x | x |  |  |
| hypothetical protein |  |  | BB0839 | 4 | 3 |  |  | x | x | x |  |
| hypothetical protein |  |  | BBA30 | 4 | 3 |  | x | x |  | x |  |
| hypothetical protein |  |  | BBB26 | 4 | 3 |  | x |  |  | x |  |
| hypothetical protein |  |  | BBG10 | 4 | 4 | x | x |  |  | x |  |
| hypothetical protein |  |  | BBG24 | 4 | 3 | x |  | x |  | x |  |
| hypothetical protein |  |  | BBP12 | 4 | 2 | x |  | x | x |  |  |
| B_burgdorferi_B31:3395_5188 |  |  | NA | 4 | 2 | x |  | x | x |  |  |
| B_burgdorferi_B31:28294_28704 |  |  | NA | 4 | 2 |  |  | x | x | x |  |
| basic membrane protein C | bmpC | Cell envelope | BB0384 | 3 | 3 |  | x |  |  | x |  |
| serine-type D-Ala-D-Ala carboxypeptidase | dacA | Cell envelope | BB0605 | 3 | 3 |  |  |  | x | x |  |
| flagellar biosynthesis protein | fliP | Cellular processes | BB0275 | 3 | 2 |  |  | x | x |  |  |
| hemolysin accessory protein | blyB | Cellular processes | BBS24 | 3 | 1 |  |  | x |  | x |  |
| rep helicase, single-stranded DNA-dependent ATPase | rep | DNA metabolism | BB0607 | 3 | 3 |  |  | x |  | x |  |
| adenine specific DNA methyltransferase |  | DNA metabolism | BBQ67 | 3 | 3 |  |  | x |  | x |  |
| beta-glucosidase, putative |  | Energy metabolism | BB0620 | 3 | 3 |  |  | x | x | x |  |
| conserved hypothetical integral membrane protein |  | Hypothetical proteins | BB0017 | 3 | 1 |  |  |  | x | x |  |
| conserved hypothetical protein |  | Hypothetical proteins | BB0070 | 3 | 3 |  |  |  | x | x |  |
| conserved hypothetical protein |  | Hypothetical proteins | BB0673 | 3 | 3 |  |  |  | x | x |  |
| conserved hypothetical protein |  | Hypothetical proteins | BB0697 | 3 | 3 | x | x |  |  | x |  |
| conserved hypothetical protein |  | Hypothetical proteins | BBA14 | 3 | 2 |  |  | x |  | x |  |
| conserved hypothetical protein |  | Hypothetical proteins | BBA45 | 3 | 2 | x |  |  |  | x |  |
| conserved hypothetical protein, pseudogene |  | Hypothetical proteins | BBI40 | 3 | 2 |  |  |  |  | x |  |
| conserved hypothetical protein |  | Hypothetical proteins | BBL29 | 3 | 1 |  |  |  | x |  |  |
| conserved hypothetical protein |  | Hypothetical proteins | BBM22 | 3 | 2 |  |  |  | x | x |  |
| conserved hypothetical protein |  | Hypothetical proteins | BBN31 | 3 | 2 | x |  | x |  | x |  |
| conserved hypothetical protein |  | Hypothetical proteins | BBP22 | 3 | 2 |  |  | x | x | x |  |
| conserved hypothetical protein |  | Hypothetical proteins | BBQ37 | 3 | 2 |  | x | x | x |  |  |
| conserved hypothetical protein |  | Hypothetical proteins | BBQ39 | 3 | 3 | x |  |  |  |  |  |
| conserved hypothetical protein |  | Hypothetical proteins | BBR06 | 3 | 2 |  |  |  | x | x |  |
| conserved hypothetical protein |  | Hypothetical proteins | BBR41 | 3 | 2 |  |  | x |  | x |  |
| peptidyl-tRNA hydrolase | pth | Protein synthesis | BB0787 | 3 | 2 | x |  |  |  | x |  |
| tRNA pseudouridine 55 synthase | truB | Protein synthesis | BB0803 | 3 | 2 |  | x | x | x |  |  |
| adenylyl cyclase, CyaB-type, putative |  | Regulatory functions | BB0723 | 3 | 3 |  |  |  |  | x |  |
| ribonuclease H | rnhB | Transcription | BB0046 | 3 | 2 | x | x |  |  |  |  |
| ribonuclease P protein component | rnpA | Transcription | BB0441 | 3 | 1 |  |  | x | x |  |  |
| oligopeptide ABC transporter, permease protein | oppC-2 | Transport and binding proteins | BB0746 | 3 | 1 |  |  | x |  | x |  |
| hypothetical protein |  |  | BB0027 | 3 | 3 |  | x |  | x |  |  |
| hypothetical protein |  |  | BB0102 | 3 | 2 |  |  | x |  | x |  |
| hypothetical protein |  |  | BB0511 | 3 | 2 |  |  |  |  | x |  |
| hypothetical protein |  |  | BB0555 | 3 | 1 | x |  | x |  | x |  |
| hypothetical protein |  |  | BB0666 | 3 | 2 |  |  |  | x | x |  |
| hypothetical protein |  |  | BB0823 | 3 | 2 |  |  |  | x | x |  |
| hypothetical protein |  |  | BBA57 | 3 | 3 |  |  | x |  | x |  |
| hypothetical protein |  |  | BBA65 | 3 | 2 |  |  | x |  | x |  |
| hypothetical protein |  |  | BBB24 | 3 | 2 |  |  | x | x |  |  |
| hypothetical protein |  |  | BBB27 | 3 | 2 | x |  |  | x |  |  |
| hypothetical protein |  |  | BBE02 | 3 | 3 |  | x |  |  | x |  |
| hypothetical protein |  |  | BBG13 | 3 | 3 | x |  |  |  | x |  |
| hypothetical protein |  |  | BBG17 | 3 | 3 |  |  |  | x | x |  |
| hypothetical protein |  |  | BBG30 | 3 | 1 | x |  |  |  | x |  |
| hypothetical protein |  |  | BBH03 | 3 | 1 |  |  |  | x | x |  |
| hypothetical protein |  |  | BBJ01 | 3 | 1 | x |  | x |  | x |  |
| hypothetical protein |  |  | BBL12 | 3 | 1 |  |  | x | x |  |  |
| hypothetical protein |  |  | BBR15 | 3 | 1 | x |  |  | x | x |  |
| ORFZ01949 |  |  | ORFZ01949 | 3 | 2 |  |  | x |  | x |  |
| antigen, P35, putative |  | Cell envelope | BBE31 | 2 | 2 |  |  |  |  | x |  |
| erpK protein | erpK | Cell envelope | BBM38 | 2 | 2 |  |  |  | x | x |  |
| erpA protein | erpA | Cell envelope | BBN38 | 2 | 2 |  |  |  |  | x |  |
| erpL protein | erpL | Cell envelope | BBO39 | 2 | 2 |  |  | x |  | x |  |
| lipoprotein | lp | Cell envelope | BBR28 | 2 | 2 | x |  |  |  |  |  |
| outer surface protein G | ospG | Cell envelope | BBS41 | 2 | 2 |  |  | x |  | x |  |
| flagellar protein | flbC | Cellular processes | BB0285 | 2 | 2 |  | x | x |  |  |  |
| flagellar basal-body rod protein | flgB | Cellular processes | BB0294 | 2 | 1 |  |  |  | x | x |  |
| flagellar protein | flaJ | Cellular processes | BB0550 | 2 | 2 | x |  |  |  |  |  |
| replicative DNA helicase, putative |  | DNA metabolism | BBG32 | 2 | 2 |  |  |  | x | x |  |
| 4-alpha-glucanotransferase | malQ | Energy metabolism | BB0166 | 2 | 2 | x |  |  | x |  |  |
| holo-acyl-carrier protein synthase, putative |  | Fatty acid and phospholipid metabolism | BB0010 | 2 | 1 |  |  | x |  | x |  |
| conserved hypothetical protein |  | Hypothetical proteins | BB0455 | 2 | 1 |  | x | x |  |  |  |
| conserved hypothetical protein |  | Hypothetical proteins | BB0740 | 2 | 2 | x |  |  |  | x |  |
| conserved hypothetical protein |  | Hypothetical proteins | BBA08 | 2 | 2 |  |  | x |  | x |  |
| conserved hypothetical protein |  | Hypothetical proteins | BBA10 | 2 | 1 |  |  | x |  |  |  |
| conserved hypothetical protein |  | Hypothetical proteins | BBA42 | 2 | 2 |  |  | x |  | x |  |
| hypothetical protein |  | Hypothetical proteins | BBB14 | 2 | 1 | x |  |  |  | x |  |
| conserved hypothetical protein |  | Hypothetical proteins | BBC01 | 2 | 1 |  | x | x |  |  |  |
| conserved hypothetical protein |  | Hypothetical proteins | BBF26 | 2 | 2 |  |  | x |  | x |  |
| conserved hypothetical protein |  | Hypothetical proteins | BBG07 | 2 | 2 | x | x |  |  |  |  |
| conserved hypothetical protein |  | Hypothetical proteins | BBG25 | 2 | 1 |  | x |  |  | x |  |
| conserved hypothetical protein |  | Hypothetical proteins | BBL43 | 2 | 1 |  | x | x |  |  |  |
| conserved hypothetical protein |  | Hypothetical proteins | BBM20 | 2 | 1 |  |  |  | x | x |  |
| conserved hypothetical protein |  | Hypothetical proteins | BBM31 | 2 | 2 |  |  | x | x |  |  |
| conserved hypothetical protein |  | Hypothetical proteins | BBN30 | 2 | 2 |  |  | x | x |  |  |
| conserved hypothetical protein |  | Hypothetical proteins | BBQ38 | 2 | 2 |  |  | x |  | x |  |
| conserved hypothetical protein |  | Hypothetical proteins | BBS22 | 2 | 1 |  |  |  |  | x |  |
| conserved hypothetical protein |  | Hypothetical proteins | BBT06 | 2 | 2 |  | x |  |  | x |  |
| S-adenosylmethionine: tRNA ribosyltransferase-isomerase |  | Protein synthesis | BB0021 | 2 | 2 |  |  | x |  | x |  |
| 2-methylthio-N6-isopentyladenosine tRNA modification enzyme | miaA | Protein synthesis | BB0821 | 2 | 2 |  |  |  | x |  |  |
| chpAI protein, putative |  | Regulatory functions | BBA07 | 2 | 1 |  |  |  | x | x |  |
| RNA polymerase sigma-54 factor | ntrA | Transcription | BB0450 | 2 | 2 | x |  |  |  | x |  |
| gufA protein |  | Unknown function | BB0219 | 2 | 1 |  |  | x |  | x |  |
| hypothetical protein |  |  | BB0019 | 2 | 2 |  |  | x | x |  |  |
| hypothetical protein |  |  | BB0110 | 2 | 2 |  |  |  | x |  |  |
| hypothetical protein |  |  | BB0172 | 2 | 2 | x |  | x |  |  |  |
| hypothetical protein |  |  | BB0174 | 2 | 2 |  |  |  |  | x |  |
| hypothetical protein |  |  | BB0255 | 2 | 2 |  |  | x | x |  |  |
| hypothetical protein |  |  | BB0322 | 2 | 2 |  |  |  | x | x |  |
| hypothetical protein |  |  | BB0354 | 2 | 2 | x |  |  |  | x |  |
| hypothetical protein |  |  | BB0428 | 2 | 1 |  |  | x | x |  |  |
| hypothetical protein |  |  | BB0460 | 2 | 2 |  |  |  | x | x |  |
| hypothetical protein |  |  | BB0563 | 2 | 2 | x |  |  | x |  |  |
| hypothetical protein |  |  | BB0714 | 2 | 2 |  |  | x |  | x |  |
| hypothetical protein |  |  | BB0743 | 2 | 1 | x |  |  |  | x |  |
| hypothetical protein |  |  | BB0748 | 2 | 2 |  |  | x |  |  |  |
| hypothetical protein |  |  | BB0792 | 2 | 2 | x |  |  | x |  |  |
| hypothetical protein |  |  | BB0824 | 2 | 1 |  |  | x | x |  |  |
| hypothetical protein |  |  | BB0826 | 2 | 2 |  |  |  | x |  |  |
| hypothetical protein |  |  | BBA49 | 2 | 1 | x |  | x |  |  |  |
| hypothetical protein |  |  | BBA53 | 2 | 2 |  |  |  |  | x |  |
| hypothetical protein |  |  | BBB25 | 2 | 2 |  |  | x |  | x |  |
| hypothetical protein |  |  | BBG21 | 2 | 2 |  |  |  | x |  |  |
| hypothetical protein |  |  | BBK03 | 2 | 1 | x |  | x |  |  |  |
| hypothetical protein |  |  | BBK23 | 2 | 2 |  | x |  |  | x |  |
| hypothetical protein |  |  | BBO12 | 2 | 1 |  |  | x | x |  |  |
| hypothetical protein |  |  | BBR12 | 2 | 1 | x | x |  |  |  |  |
| ORFZ01805 |  |  | ORFZ01805 | 2 | 1 |  |  |  | x | x |  |
| ORFZ01936 |  |  | ORFZ01936 | 2 | 1 |  |  |  | x | x |  |
| ORFZ01938 |  |  | ORFZ01938 | 2 | 2 |  |  | x | x |  |  |
| B_burgdorferi_B31:468114_468458 |  |  | NA | 2 | 2 | x |  |  |  |  |  |
| B_burgdorferi_B31:37927_38331 |  |  | NA | 2 | 2 | x |  |  |  |  |  |
| B_burgdorferi_B31:468843_469106 |  |  | NA | 2 | 2 | x |  |  | x |  |  |
| B_burgdorferi_B31:14923_15177 |  |  | NA | 2 | 2 | x |  |  | x |  |  |
| B_burgdorferi_B31:18108_18329 |  |  | NA | 2 | 2 |  |  |  |  | x |  |
| B_burgdorferi_B31:482328_482501 |  |  | NA | 2 | 2 |  |  |  | x | x |  |
| B_burgdorferi_B31:10624_10878 |  |  | NA | 2 | 2 |  |  |  | x | x |  |
| UDP-N-acetylmuramoylalanyl-D-glutamyl-2,6-diaminopimelate--D-alanyl-D-alanine ligase | murF | Cell envelope | BB0304 | 1 | 1 |  |  |  |  | x |  |
| membrane spanning protein, putative |  | Cell envelope | BB0753 | 1 | 1 |  |  |  |  | x |  |
| exported protein A | eppA | Cell envelope | BBC06 | 1 | 1 |  |  |  |  | x |  |
| protein p23 |  | Cell envelope | BBE09 | 1 | 1 |  |  |  | x |  |  |
| antigen, P35, putative |  | Cell envelope | BBK15 | 1 | 1 | x |  |  |  |  |  |
| immunogenic protein P37, putative |  | Cell envelope | BBK48 | 1 | 1 |  |  | x |  |  |  |
| lipoprotein |  | Cell envelope | BBL28 | 1 | 1 |  |  |  | x |  |  |
| lipoprotein | lp | Cell envelope | BBS30 | 1 | 1 |  |  |  | x |  |  |
| flagellar biosynthesis protein | flhB | Cellular processes | BB0272 | 1 | 1 |  |  |  |  | x |  |
| cell division protein | divIB | Cellular processes | BB0301 | 1 | 1 |  |  |  | x |  |  |
| competence protein F, putative |  | Cellular processes | BB0798 | 1 | 1 |  |  | x |  |  |  |
| multidrug-efflux transporter |  | Cellular processes | BBI26 | 1 | 1 | x |  |  |  |  |  |
| plasmid partition protein, putative |  | Cellular processes | BBM32 | 1 | 1 |  |  |  |  | x |  |
| glucose inhibited division protein B | gidB | DNA metabolism | BB0177 | 1 | 1 |  |  |  | x |  |  |
| 3-methyladenine DNA glycosylase | mag | DNA metabolism | BB0422 | 1 | 1 |  |  |  | x |  |  |
| xylulokinase | xylB | Energy metabolism | BB0545 | 1 | 1 | x |  |  |  |  |  |
| phosphatidyltransferase |  | Fatty acid and phospholipid metabolism | BB0249 | 1 | 1 |  |  |  |  | x |  |
| conserved hypothetical protein |  | Hypothetical proteins | BB0143 | 1 | 1 | x |  |  |  |  |  |
| conserved hypothetical protein |  | Hypothetical proteins | BB0206 | 1 | 1 |  |  |  |  | x |  |
| conserved hypothetical protein |  | Hypothetical proteins | BB0439 | 1 | 1 | x |  |  |  |  |  |
| conserved hypothetical protein |  | Hypothetical proteins | BBA18 | 1 | 1 |  | x |  |  |  |  |
| conserved hypothetical protein |  | Hypothetical proteins | BBB23 | 1 | 1 |  |  |  | x |  |  |
| conserved hypothetical protein |  | Hypothetical proteins | BBC11 | 1 | 1 |  |  |  | x |  |  |
| conserved hypothetical protein |  | Hypothetical proteins | BBE18 | 1 | 1 |  |  |  |  | x |  |
| conserved hypothetical protein |  | Hypothetical proteins | BBF03 | 1 | 1 |  |  |  |  | x |  |
| conserved hypothetical protein |  | Hypothetical proteins | BBG06 | 1 | 1 |  | x |  |  |  |  |
| conserved hypothetical protein |  | Hypothetical proteins | BBG27 | 1 | 1 |  |  |  |  | x |  |
| conserved hypothetical protein |  | Hypothetical proteins | BBH27 | 1 | 1 |  |  |  |  | x |  |
| conserved hypothetical protein |  | Hypothetical proteins | BBL06 | 1 | 1 |  |  | x |  |  |  |
| conserved hypothetical protein |  | Hypothetical proteins | BBL22 | 1 | 1 |  |  |  | x |  |  |
| conserved hypothetical protein |  | Hypothetical proteins | BBL30 | 1 | 1 |  |  |  |  | x |  |
| conserved hypothetical protein |  | Hypothetical proteins | BBM30 | 1 | 1 | x |  |  |  |  |  |
| conserved hypothetical protein |  | Hypothetical proteins | BBM35 | 1 | 1 |  |  |  |  | x |  |
| conserved hypothetical protein |  | Hypothetical proteins | BBM37 | 1 | 1 |  |  |  | x |  |  |
| conserved hypothetical protein |  | Hypothetical proteins | BBO30 | 1 | 1 |  |  |  |  | x |  |
| conserved hypothetical protein |  | Hypothetical proteins | BBP30 | 1 | 1 |  |  |  |  | x |  |
| conserved hypothetical protein |  | Hypothetical proteins | BBQ07 | 1 | 1 |  |  |  |  | x |  |
| conserved hypothetical protein |  | Hypothetical proteins | BBQ13 | 1 | 1 |  |  | x |  |  |  |
| conserved hypothetical protein |  | Hypothetical proteins | BBQ29 | 1 | 1 |  |  | x |  |  |  |
| conserved hypothetical protein |  | Hypothetical proteins | BBR36 | 1 | 1 |  |  | x |  |  |  |
| conserved hypothetical protein |  | Hypothetical proteins | BBR38 | 1 | 1 |  |  |  |  | x |  |
| conserved hypothetical protein |  | Hypothetical proteins | BBS06 | 1 | 1 |  |  |  | x |  |  |
| conserved hypothetical protein |  | Hypothetical proteins | BBS33 | 1 | 1 |  |  |  | x |  |  |
| conserved hypothetical protein |  | Hypothetical proteins | BBU04 | 1 | 1 |  |  |  |  | x |  |
| tRNA-guanine transglycosylase | tgt | Protein synthesis | BB0809 | 1 | 1 |  |  |  |  | x |  |
| phosphocarrier protein HPr | ptsH-1 | Transport and binding proteins | BB0448 | 1 | 1 | x |  |  |  |  |  |
| ribose/galactose ABC transporter, permease protein | rbsC-1 | Transport and binding proteins | BB0678 | 1 | 1 |  |  |  |  | x |  |
| ribose/galactose ABC transporter, permease protein | rbsC-2 | Transport and binding proteins | BB0679 | 1 | 1 |  |  |  | x |  |  |
| inositol monophosphatase |  | Unknown function | BB0524 | 1 | 1 | x |  |  |  |  |  |
| hypothetical protein |  |  | BB0208 | 1 | 1 |  |  | x |  |  |  |
| hypothetical protein |  |  | BB0223 | 1 | 1 |  |  | x |  |  |  |
| hypothetical protein |  |  | BB0242 | 1 | 1 |  |  |  |  | x |  |
| hypothetical protein |  |  | BB0266 | 1 | 1 |  | x |  |  |  |  |
| hypothetical protein |  |  | BB0305 | 1 | 1 |  |  |  | x |  |  |
| hypothetical protein |  |  | BB0399 | 1 | 1 |  | x |  |  |  |  |
| hypothetical protein |  |  | BB0452 | 1 | 1 | x |  |  |  |  |  |
| hypothetical protein |  |  | BB0456 | 1 | 1 |  |  |  |  | x |  |
| hypothetical protein |  |  | BB0530 | 1 | 1 | x |  |  |  |  |  |
| hypothetical protein |  |  | BB0554 | 1 | 1 | x |  |  |  |  |  |
| hypothetical protein |  |  | BB0758 | 1 | 1 |  | x |  |  |  |  |
| hypothetical protein |  |  | BBA33 | 1 | 1 |  |  |  | x |  |  |
| hypothetical protein |  |  | BBA37 | 1 | 1 |  |  |  | x |  |  |
| hypothetical protein |  |  | BBA56 | 1 | 1 | x |  |  |  |  |  |
| hypothetical protein |  |  | BBE17 | 1 | 1 |  |  |  | x |  |  |
| hypothetical protein |  |  | BBF10 | 1 | 1 |  |  |  |  | x |  |
| hypothetical protein |  |  | BBG22 | 1 | 1 |  |  | x |  |  |  |
| hypothetical protein |  |  | BBG26 | 1 | 1 |  |  | x |  |  |  |
| hypothetical protein |  |  | BBI27 | 1 | 1 |  |  |  | x |  |  |
| hypothetical protein |  |  | BBI28 | 1 | 1 |  |  |  |  | x |  |
| hypothetical protein |  |  | BBI34 | 1 | 1 |  |  |  | x |  |  |
| hypothetical protein |  |  | BBJ31 | 1 | 1 |  |  |  | x |  |  |
| hypothetical protein |  |  | BBK19 | 1 | 1 |  |  |  |  | x |  |
| hypothetical protein |  |  | BBM05 | 1 | 1 |  |  | x |  |  |  |
| hypothetical protein |  |  | BBM11 | 1 | 1 |  |  |  | x |  |  |
| hypothetical protein |  |  | BBM18 | 1 | 1 | x |  |  |  |  |  |
| hypothetical protein |  |  | BBM39 | 1 | 1 |  |  |  |  | x |  |
| hypothetical protein |  |  | BBM41 | 1 | 1 |  |  |  | x |  |  |
| hypothetical protein |  |  | BBN02 | 1 | 1 |  | x |  |  |  |  |
| hypothetical protein |  |  | BBO04 | 1 | 1 |  |  |  |  | x |  |
| hypothetical protein |  |  | BBO11 | 1 | 1 |  |  |  | x |  |  |
| hypothetical protein |  |  | BBO14 | 1 | 1 |  |  |  |  | x |  |
| hypothetical protein |  |  | BBO29 | 1 | 1 |  |  |  | x |  |  |
| hypothetical protein |  |  | BBO43 | 1 | 1 |  |  | x |  |  |  |
| hypothetical protein |  |  | BBP03 | 1 | 1 |  |  |  |  | x |  |
| hypothetical protein |  |  | BBP11 | 1 | 1 |  |  | x |  |  |  |
| hypothetical protein |  |  | BBP41 | 1 | 1 |  |  |  | x |  |  |
| hypothetical protein |  |  | BBQ23 | 1 | 1 |  |  |  | x |  |  |
| hypothetical protein |  |  | BBQ24 | 1 | 1 | x |  |  |  |  |  |
| hypothetical protein |  |  | BBQ25 | 1 | 1 | x |  |  |  |  |  |
| hypothetical protein |  |  | BBQ48 | 1 | 1 |  |  |  | x |  |  |
| hypothetical protein, paralogous family 60, pseudogene |  |  | BBQ80 | 1 | 1 |  |  |  |  | x |  |
| hypothetical protein |  |  | BBR11 | 1 | 1 |  |  |  |  | x |  |
| hypothetical protein |  |  | BBR17 | 1 | 1 |  |  |  |  | x |  |
| hypothetical protein |  |  | BBR43 | 1 | 1 |  | x |  |  |  |  |
| hypothetical protein |  |  | BBR44 | 1 | 1 |  |  |  |  | x |  |
| hypothetical protein |  |  | BBS27 | 1 | 1 |  | x |  |  |  |  |
| hypothetical protein |  |  | BBU01 | 1 | 1 |  |  |  |  | x |  |
| ORFZ01827 |  |  | ORFZ01827 | 1 | 1 |  |  |  | x |  |  |
| ORFZ01876 |  |  | ORFZ01876 | 1 | 1 |  |  | x |  |  |  |
