# Supplementary material for: Proteome Analysis of Borrelia burgdorferi Response to Environmental Change
Source: PLoS One. 2010 Nov 2;5(11):e13800. doi: 10.1371/journal.pone.0013800 (PMC2970547; doi:10.1371/journal.pone.0013800)
Supplement: Table S2 — Supplementary Table S2 (0.03 MB DOC) [file pone.0013800.s003.doc]

| Supplementary table S2. Proteins unique to the RpH culture. | | |  |
| --- | --- | --- | --- |
| **Protein name** | **Gene** | **Functional annotation** | **Annotated ORF** |
| rev protein | rev | Unknown function | BBM27 |
| lipoprotein |  | Cell envelope | BBP28 |
| plasmid partition protein, putative |  | Cellular processes | BBO32 |
| chemotaxis protein methyltransferase | cheR-1 | Cellular processes | BB0040 |
| antigen, S2 |  | Cell envelope | BBA04 |
| antigen, P35, putative |  | Cell envelope | BBA73 |
| antigen, P35 |  | Cell envelope | BBA64 |
| hypothetical protein |  |  | BB0459 |
