# Supplementary material for: Proteome Analysis of Borrelia burgdorferi Response to Environmental Change
Source: PLoS One. 2010 Nov 2;5(11):e13800. doi: 10.1371/journal.pone.0013800 (PMC2970547; doi:10.1371/journal.pone.0013800)
Supplement: Table S3 — Supplementary Table S3 (0.10 MB DOC) [file pone.0013800.s004.doc]

| pantothenate metabolism flavoprotein | dfp | Biosynthesis of cofactors, prosthetic groups, and carriers | | BB0812 | 29 | 17 |
| --- | --- | --- | --- | --- | --- | --- |
| surface-located membrane protein 1 | lmp1 | | Cell envelope | BB0210 | 567 | 129 |
| lipoprotein LA7 |  | | Cell envelope | BB0365 | 812 | 27 |
| antigen, S2 |  | | Cell envelope | BBA04 | 6 | 5 |
| decorin binding protein A | dbpA | | Cell envelope | BBA24 | 294 | 42 |
| decorin binding protein B | dbpB | | Cell envelope | BBA25 | 90 | 24 |
| lipoprotein |  | | Cell envelope | BBA36 | 9 | 2 |
| antigen, P35 |  | | Cell envelope | BBA64 | 18 | 9 |
| antigen, P35, putative |  | | Cell envelope | BBA66 | 64 | 12 |
| antigen, P35, putative |  | | Cell envelope | BBA73 | 8 | 6 |
| outer surface protein C | ospC | | Cell envelope | BBB19 | 2412 | 79 |
| immunogenic protein P35 |  | | Cell envelope | BBK32 | 5 | 3 |
| immunogenic protein P37 |  | | Cell envelope | BBK50 | 11 | 6 |
| lipoprotein | lp | | Cell envelope | BBM28 | 4 | 2 |
| lipoprotein |  | | Cell envelope | BBP28 | 9 | 5 |
| lipoprotein | lp | | Cell envelope | BBS30 | 1 | 1 |
| chemotaxis protein methyltransferase | cheR-1 | | Cellular processes | BB0040 | 6 | 4 |
| flagellar protein | fliL | | Cellular processes | BB0279 | 65 | 8 |
| protein-glutamate methylesterase | cheB-1 | | Cellular processes | BB0415 | 32 | 12 |
| chromosome segregation protein, putative |  | | Cellular processes | BB0431 | 97 | 27 |
| plasmid partition protein, putative |  | | Cellular processes | BBR33 | 29 | 10 |
| hemolysin accessory protein | blyB | | Cellular processes | BBS24 | 3 | 1 |
| S-adenosylmethionine synthetase | metK | | Central intermediary metabolism | BB0376 | 152 | 31 |
| DNA mismatch repair protein | mutL | | DNA metabolism | BB0211 | 83 | 28 |
| excinuclease ABC, subunit C | uvrC | | DNA metabolism | BB0457 | 36 | 21 |
| ATP-dependent helicase | hrpA | | DNA metabolism | BB0827 | 204 | 50 |
| conserved hypothetical integral membrane protein |  | | Hypothetical proteins | BB0017 | 3 | 1 |
| conserved hypothetical protein |  | | Hypothetical proteins | BB0086 | 61 | 26 |
| conserved hypothetical protein |  | | Hypothetical proteins | BB0175 | 12 | 6 |
| conserved hypothetical protein |  | | Hypothetical proteins | BB0377 | 18 | 8 |
| conserved hypothetical protein |  | | Hypothetical proteins | BB0467 | 34 | 8 |
| conserved hypothetical protein |  | | Hypothetical proteins | BB0740 | 2 | 2 |
| conserved hypothetical protein |  | | Hypothetical proteins | BB0819 | 16 | 3 |
| conserved hypothetical protein |  | | Hypothetical proteins | BBQ13 | 1 | 1 |
| conserved hypothetical protein |  | | Hypothetical proteins | BBS22 | 2 | 1 |
| conserved hypothetical protein |  | | Hypothetical proteins | BBS31 | 4 | 4 |
| conserved hypothetical protein |  | | Hypothetical proteins | BBS34 | 12 | 3 |
| Lambda CII stability-governing protein | hflK | | Protein fate | BB0203 | 419 | 39 |
| apolipoprotein N-acyltransferase, putative |  | | Protein fate | BB0237 | 12 | 8 |
| zinc protease, putative |  | | Protein fate | BB0536 | 472 | 98 |
| aspartyl-tRNA synthetase | aspS | | Protein synthesis | BB0446 | 159 | 41 |
| RNA polymerase sigma factor | rpoS | | Transcription | BB0771 | 7 | 5 |
| phosphate ABC transporter, ATP-binding protein | pstB | | Transport and binding proteins | BB0218 | 45 | 13 |
| Na+/H+ antiporter | napA | | Transport and binding proteins | BB0447 | 11 | 5 |
| outer membrane porin | oms28 | | Transport and binding proteins | BBA74 | 636 | 71 |
| PTS system, cellobiose-specific IIA component | celC | | Transport and binding proteins | BBB05 | 99 | 13 |
| PTS system, cellobiose-specific IIB component | celA | | Transport and binding proteins | BBB06 | 35 | 8 |
| hypothetical protein |  | |  | BB0013 | 8 | 6 |
| hypothetical protein |  | |  | BB0075 | 12 | 7 |
| hypothetical protein |  | |  | BB0077 | 7 | 4 |
| hypothetical protein |  | |  | BB0126 | 47 | 13 |
| hypothetical protein |  | |  | BB0162 | 20 | 4 |
| hypothetical protein |  | |  | BB0374 | 31 | 15 |
| hypothetical protein |  | |  | BB0405 | 20 | 5 |
| hypothetical protein |  | |  | BB0459 | 10 | 6 |
| hypothetical protein |  | |  | BB0689 | 49 | 18 |
| hypothetical protein |  | |  | BB0751 | 505 | 74 |
| hypothetical protein |  | |  | BB0832 | 18 | 10 |
| hypothetical protein |  | |  | BBA37 | 1 | 1 |
| hypothetical protein |  | |  | BBA57 | 3 | 3 |
| hypothetical protein |  | |  | BBA65 | 3 | 2 |
| hypothetical protein |  | |  | BBA69 | 98 | 34 |
| hypothetical protein |  | |  | BBP12 | 4 | 2 |
| hypothetical protein |  | |  | BBQ24 | 1 | 1 |
| hypothetical protein |  | |  | BBR12 | 2 | 1 |
| hypothetical protein |  | |  | BBR17 | 1 | 1 |
