# Supplementary material for: Proteome Analysis of Borrelia burgdorferi Response to Environmental Change
Source: PLoS One. 2010 Nov 2;5(11):e13800. doi: 10.1371/journal.pone.0013800 (PMC2970547; doi:10.1371/journal.pone.0013800)
Supplement: Table S4 — Supplementary Table S4 (0.27 MB DOC) [file pone.0013800.s005.doc]

| ornithine carbamoyltransferase, catabolic | arcB | Energy metabolism | BB0842 | 42 | 22 |
| --- | --- | --- | --- | --- | --- |
| excinuclease ABC, subunit A | uvrA | DNA metabolism | BB0837 | 34 | 22 |
| hypothetical protein |  |  | BB0024 | 25 | 19 |
| conserved hypothetical protein |  | Hypothetical proteins | BB0129 | 20 | 14 |
| glucose-6-phosphate 1-dehydrogenase, putative |  | Energy metabolism | BB0222 | 18 | 12 |
| transcription factor, putative |  | Transcription | BB0355 | 18 | 9 |
|  |  |  | ORFZ01931 | 16 | 11 |
| arginine deiminase | arcA | Energy metabolism | BB0841 | 16 | 11 |
| hypothetical protein |  |  | BBH18 | 13 | 10 |
| translation initiation factor 1 | infA | Protein synthesis | BB0169 | 12 | 6 |
| conserved hypothetical protein |  | Hypothetical proteins | BBQ41 | 12 | 7 |
| conserved hypothetical protein |  | Hypothetical proteins | BBS37 | 12 | 10 |
| hypothetical protein |  |  | BBJ36 | 12 | 10 |
| aldose reductase, putative |  | Unknown function | BB0528 | 12 | 9 |
| hypothetical protein |  |  | BB0072 | 12 | 10 |
| conserved hypothetical protein |  | Hypothetical proteins | BBJ16 | 11 | 9 |
| ribosomal protein L35 | rpmI | Protein synthesis | BB0189 | 11 | 5 |
| ribosomal protein L33 | rpmG | Protein synthesis | BB0396 | 11 | 4 |
| lipoprotein |  | Cell envelope | BBA62 | 11 | 6 |
| cytidine deaminase | cdd | Purines, pyrimidines, nucleosides, and nucleotides | BB0618 | 11 | 9 |
| response regulatory protein | rrp-2 | Regulatory functions | BB0763 | 11 | 9 |
| DNA topoisomerase I | topA | DNA metabolism | BB0828 | 11 | 9 |
| plasmid partition protein, putative |  | Cellular processes | BBJ17 | 10 | 6 |
| single-stranded-DNA-specific exonuclease | recJ | DNA metabolism | BB0254 | 10 | 9 |
| hypothetical protein |  |  | BB0345 | 10 | 9 |
| plasmid partition protein, putative |  | Cellular processes | BBA20 | 10 | 3 |
| excinuclease ABC, subunit C | uvrC | DNA metabolism | BB0457 | 10 | 10 |
| DNA polymerase III, subunits gamma and tau | dnaX | DNA metabolism | BB0461 | 10 | 9 |
| ATP-dependent protease LA | lon-2 | Protein fate | BB0613 | 10 | 8 |
| uridine kinase | udk | Purines, pyrimidines, nucleosides, and nucleotides | BB0015 | 9 | 7 |
| conserved hypothetical protein |  | Hypothetical proteins | BB0225 | 9 | 7 |
| flagellar biosynthesis protein | flhA | Cellular processes | BB0271 | 9 | 7 |
| response regulatory protein | rrp-1 | Regulatory functions | BB0419 | 9 | 5 |
| hypothetical protein |  |  | BB0509 | 9 | 5 |
| GTP-binding protein | obg | Cellular processes | BB0781 | 9 | 7 |
| excinuclease ABC, subunit B | uvrB | DNA metabolism | BB0836 | 9 | 6 |
| hypothetical protein |  |  | BB0009 | 9 | 6 |
| chemotaxis response regulator | cheY-1 | Cellular processes | BB0551 | 8 | 6 |
| glucose-6-phosphate 1-dehydrogenase | zwf | Energy metabolism | BB0636 | 8 | 8 |
| conserved hypothetical protein |  | Hypothetical proteins | BBG09 | 8 | 7 |
| protein-export membrane protein | secF | Protein fate | BB0653 | 8 | 5 |
| conserved hypothetical protein |  | Hypothetical proteins | BBH29 | 7 | 5 |
| adenylate kinase | adk | Purines, pyrimidines, nucleosides, and nucleotides | BB0417 | 7 | 6 |
| N-acetylmuramoyl-L-alanine amidase, putative |  | Cell envelope | BB0625 | 7 | 6 |
| hypothetical protein |  |  | BB0760 | 7 | 5 |
| UDP-N-acetylglucosamine--N-acetylmuramyl-(pentapeptide) pyrophosphoryl-undecaprenol N-acetylglucosamine transferase | murG | Cell envelope | BB0767 | 7 | 6 |
| hypothetical protein |  |  | BB0838 | 7 | 7 |
| zinc protease, putative |  | Protein fate | BB0118 | 6 | 6 |
| plasmid partition protein, putative |  | Cellular processes | BBQ40 | 6 | 5 |
| conserved hypothetical protein |  | Hypothetical proteins | BBK40 | 6 | 6 |
| hypothetical protein |  |  | BBJ08 | 6 | 6 |
| minD-related ATP-binding protein | ylxH-1 | Cellular processes | BB0269 | 6 | 6 |
| hypothetical protein |  |  | BB0324 | 6 | 6 |
| hypothetical protein |  |  | BBH09 | 6 | 2 |
| GTP-binding protein | era | Cellular processes | BB0660 | 6 | 5 |
| hypothetical protein |  |  | BB0082 | 6 | 4 |
| conserved hypothetical protein |  | Hypothetical proteins | BBH13 | 6 | 4 |
| primosomal protein N' | priA | DNA metabolism | BB0014 | 5 | 5 |
| hypothetical protein |  |  | BB0162 | 5 | 2 |
| hypothetical protein |  |  | BB0170 | 5 | 3 |
| plasmid partition protein, putative |  | Cellular processes | BBN32 | 5 | 5 |
| flagellar basal-body rod protein | flgC | Cellular processes | BB0293 | 5 | 5 |
| octaprenyl-diphosphate synthase | ispB | Biosynthesis of cofactors, prosthetic groups, and carriers | BB0314 | 5 | 5 |
| hypothetical protein |  | Hypothetical proteins | BBD18 | 5 | 3 |
| conserved hypothetical protein |  | Hypothetical proteins | BBA43 | 5 | 4 |
|  |  |  | B_burgdorferi_B31:501012_501212 | 5 | 2 |
| thioredoxin reductase | trxB | Energy metabolism | BB0515 | 5 | 3 |
| conserved hypothetical protein |  | Hypothetical proteins | BB0538 | 5 | 5 |
| dimethyladenosine transferase | ksgA | Protein synthesis | BB0590 | 5 | 5 |
| conserved hypothetical protein |  | Hypothetical proteins | BB0682 | 5 | 4 |
| penicillin-binding protein | pbp-2 | Cell envelope | BB0718 | 5 | 5 |
| hypothetical protein |  |  | BB0832 | 5 | 4 |
| replicative DNA helicase | dnaB | DNA metabolism | BB0111 | 4 | 3 |
| hypothetical protein |  |  | BB0013 | 4 | 4 |
| penicillin-binding protein | pbp-1 | Cell envelope | BB0136 | 4 | 3 |
| plasmid partition protein, putative |  | Cellular processes | BBH28 | 4 | 4 |
| conserved hypothetical protein |  | Hypothetical proteins | BBC03 | 4 | 4 |
| conserved hypothetical protein |  | Hypothetical proteins | BBP31 | 4 | 3 |
| conserved hypothetical protein |  | Hypothetical proteins | BBO33 | 4 | 3 |
| methylenetetrahydrofolate dehydrogenase | folD | Biosynthesis of cofactors, prosthetic groups, and carriers | BB0026 | 4 | 4 |
| conserved hypothetical protein |  | Hypothetical proteins | BBQ42 | 4 | 4 |
| conserved hypothetical protein |  | Hypothetical proteins | BB0268 | 4 | 3 |
| smg protein |  | Unknown function | BB0297 | 4 | 3 |
| conserved hypothetical protein |  | Hypothetical proteins | BB0306 | 4 | 4 |
| hypothetical protein |  |  | BB0309 | 4 | 4 |
| hypothetical protein |  |  | BB0367 | 4 | 3 |
| hypothetical protein |  |  | BB0374 | 4 | 4 |
| conserved hypothetical protein |  | Hypothetical proteins | BB0377 | 4 | 2 |
| conserved hypothetical protein |  | Hypothetical proteins | BBD11 | 4 | 4 |
|  |  |  | B_burgdorferi_B31:4099_1 | 4 | 4 |
| plasmid partition protein, putative |  | Hypothetical proteins | BBF24 | 4 | 2 |
| protein-glutamate methylesterase | cheB-1 | Cellular processes | BB0415 | 4 | 4 |
| hypothetical protein |  |  | BB0526 | 4 | 4 |
| hypothetical protein |  |  | BB0531 | 4 | 3 |
| hypothetical protein |  |  | BB0542 | 4 | 3 |
| hypothetical protein |  |  | BB0614 | 4 | 3 |
| exodeoxyribonuclease V, alpha chain | recD | DNA metabolism | BB0632 | 4 | 4 |
| phosphoglycolate phosphatase | gph | Energy metabolism | BB0676 | 4 | 2 |
| DNA primase | dnaG | DNA metabolism | BB0710 | 4 | 3 |
| beta-N-acetylhexosaminidase, putative |  | Energy metabolism | BB0002 | 4 | 3 |
| hypothetical protein |  |  | BB0756 | 4 | 4 |
| hypothetical protein |  |  | BB0765 | 4 | 4 |
| conserved hypothetical protein |  | Hypothetical proteins | BB0799 | 4 | 3 |
| phosphomannomutase | cpsG | Energy metabolism | BB0835 | 4 | 4 |
| cytidylate kinase | cmk | Purines, pyrimidines, nucleosides, and nucleotides | BB0128 | 3 | 3 |
| hypothetical protein |  |  | BB0155 | 3 | 3 |
| outer membrane protein | tpn50 | Cell envelope | BB0167 | 3 | 3 |
| thiophene and furan oxidation protein | thdF | Cellular processes | BB0179 | 3 | 3 |
| hypothetical protein |  |  | BB0209 | 3 | 3 |
| antigen, P35, putative |  | Cell envelope | BBH32 | 3 | 2 |
| conserved hypothetical protein |  | Hypothetical proteins | BBR34 | 3 | 2 |
| conserved hypothetical protein |  | Hypothetical proteins | BBO34 | 3 | 3 |
| conserved hypothetical protein |  | Hypothetical proteins | BBP34 | 3 | 3 |
| conserved hypothetical protein |  | Hypothetical proteins | BBQ39 | 3 | 3 |
| conserved hypothetical protein |  | Hypothetical proteins | BB0247 | 3 | 2 |
| hypothetical protein |  |  | BB0259 | 3 | 2 |
| hypothetical protein |  |  | BB0353 | 3 | 2 |
| hypothetical protein |  |  | BB0038 | 3 | 2 |
| minD-related ATP-binding protein | ylxH-2 | Cellular processes | BB0361 | 3 | 3 |
| hypothetical protein |  |  | BB0398 | 3 | 3 |
| conserved hypothetical protein |  | Hypothetical proteins | BBF23 | 3 | 3 |
| conserved hypothetical protein |  | Hypothetical proteins | BBF20 | 3 | 3 |
| thymidylate synthase-complementing protein | thy1 | Unknown function | BBA76 | 3 | 3 |
| sensory transduction histidine kinase/response regulator |  | Regulatory functions | BB0420 | 3 | 3 |
| conserved hypothetical protein |  | Hypothetical proteins | BB0505 | 3 | 3 |
| hypothetical protein |  |  | BB0562 | 3 | 3 |
| conserved hypothetical integral membrane protein |  | Hypothetical proteins | BB0616 | 3 | 3 |
| hypothetical protein |  |  | BB0654 | 3 | 3 |
| hypothetical protein |  |  | BB0075 | 3 | 3 |
| hypothetical protein |  |  | BB0077 | 3 | 3 |
| rod shape-determining protein | mreC | Cell envelope | BB0716 | 3 | 3 |
| ABC transporter, ATP-binding protein |  | Transport and binding proteins | BB0080 | 3 | 3 |
| glutamate transporter | gltP | Transport and binding proteins | BB0729 | 3 | 3 |
| sialoglycoprotease | gcp | Protein fate | BB0769 | 3 | 3 |
| hypothetical protein |  |  | BB0790 | 3 | 3 |
| V-type ATPase, subunit D | atpD | Energy metabolism | BB0092 | 3 | 2 |
| hypothetical protein |  |  | BB0852 | 3 | 3 |
| conserved hypothetical protein |  | Hypothetical proteins | BBB13 | 2 | 2 |
| hypothetical protein |  |  | BB0106 | 2 | 2 |
| hypothetical protein |  |  | BBG13 | 2 | 2 |
| hypothetical protein |  |  | BB0163 | 2 | 2 |
| lipoprotein | lp | Cell envelope | BBR28 | 2 | 2 |
| hypothetical protein |  |  | BB0171 | 2 | 2 |
| conserved hypothetical protein |  | Hypothetical proteins | BB0175 | 2 | 2 |
| conserved hypothetical protein |  | Hypothetical proteins | BB0183 | 2 | 2 |
| HemK family methylase, putative |  | Unknown function | BB0197 | 2 | 2 |
| conserved hypothetical protein |  | Hypothetical proteins | BBP33 | 2 | 2 |
|  |  |  | B_burgdorferi_B31:21974_22252 | 2 | 2 |
| flagellar motor switch protein | fliG-1 | Cellular processes | BB0221 | 2 | 2 |
| conserved hypothetical protein |  | Hypothetical proteins | BBG29 | 2 | 2 |
| PTS system, cellobiose-specific IIC component | celB | Transport and binding proteins | BBB04 | 2 | 2 |
| hypothetical protein |  |  | BB0245 | 2 | 2 |
| conserved hypothetical protein |  | Hypothetical proteins | BBG31 | 2 | 2 |
| hypothetical protein |  |  | BB0315 | 2 | 2 |
| ATP-dependent Clp protease, subunit A | clpA | Protein fate | BB0369 | 2 | 2 |
|  |  |  | B_burgdorferi_B31:37927_38331 | 2 | 2 |
| hypothetical protein, paralogous family 85 |  | Hypothetical proteins | BBD15 | 2 | 2 |
| hypothetical protein |  |  | BBA50 | 2 | 2 |
| conserved hypothetical protein |  | Hypothetical proteins | BBA11 | 2 | 2 |
| PTS system, cellobiose-specific IIB component | celA | Transport and binding proteins | BBB06 | 2 | 2 |
|  |  |  | B_burgdorferi_B31:468114_468458 | 2 | 2 |
| hemolysin | tlyC | Cellular processes | BB0059 | 2 | 2 |
| flagellar protein | flaJ | Cellular processes | BB0550 | 2 | 2 |
| hypothetical protein |  |  | BB0071 | 2 | 2 |
| conserved hypothetical GTP-binding protein |  | Hypothetical proteins | BB0643 | 2 | 2 |
| PTS system, glucose-specific IIBC component | ptsG | Transport and binding proteins | BB0645 | 2 | 2 |
| hypothetical protein |  |  | BB0665 | 2 | 2 |
| carotenoid biosynthesis protein, putative |  | Unknown function | BB0684 | 2 | 2 |
| 3-hydroxy-3-methylglutaryl-CoA reductase | mvaA | Energy metabolism | BB0685 | 2 | 2 |
| mevalonate pyrophosphate decarboxylase |  | Fatty acid and phospholipid metabolism | BB0686 | 2 | 2 |
| hypothetical protein |  |  | BB0701 | 2 | 2 |
|  |  |  | BB0707 | 2 | 2 |
| hypothetical protein |  |  | BB0007 | 2 | 2 |
| rod shape-determining protein | mreB-2 | Cell envelope | BB0719 | 2 | 2 |
| conserved hypothetical protein |  | Hypothetical proteins | BB0725 | 2 | 2 |
| sensory transduction histidine kinase, putative |  | Regulatory functions | BB0764 | 2 | 2 |
| hypothetical protein |  |  | BB0773 | 2 | 2 |
| peptidyl-tRNA hydrolase | pth | Protein synthesis | BB0787 | 2 | 2 |
| conserved hypothetical protein |  | Hypothetical proteins | BB0008 | 2 | 2 |
| hypothetical protein |  |  | BB0806 | 2 | 2 |
| pantothenate metabolism flavoprotein | dfp | Biosynthesis of cofactors, prosthetic groups, and carriers | BB0812 | 2 | 2 |
| exonuclease SbcC | sbcC | DNA metabolism | BB0830 | 2 | 2 |
|  |  |  |  |  |  |
| **Fed only** |  |  |  |  |  |
| decorin binding protein B | dbpB | Cell envelope | BBA25 | 24 | 18 |
| rev protein | rev | Unknown function | BBP27 | 9 | 7 |
| antigen, P35, putative |  | Cell envelope | BBA66 | 9 | 5 |
| conserved hypothetical protein |  | Hypothetical proteins | BBA13 | 8 | 6 |
| oligopeptide ABC transporter, periplasmic oligopeptide-binding protein | oppAV | Transport and binding proteins | BBA34 | 7 | 6 |
|  |  |  | ORFZ01935 | 6 | 5 |
| hypothetical protein |  |  | BBA48 | 5 | 4 |
| erpA protein | erpA | Cell envelope | BBL39 | 4 | 3 |
| ABC transporter, ATP-binding protein |  | Transport and binding proteins | BB0573 | 4 | 2 |
| antigen, S2 |  | Cell envelope | BBA04 | 3 | 2 |
| antigen, P35, putative |  | Cell envelope | BBA73 | 3 | 3 |
| antigen, P35 |  | Cell envelope | BBA64 | 3 | 2 |
| hypothetical protein |  |  | BBA38 | 3 | 3 |
| Na+/H+ antiporter | napA | Transport and binding proteins | BB0447 | 3 | 3 |
| NH(3)-dependent NAD+ synthetase |  | Biosynthesis of cofactors, prosthetic groups, and carriers | BB0522 | 3 | 3 |
| ferric uptake regulation protein | fur | Regulatory functions | BB0647 | 3 | 3 |
| rev protein | rev | Unknown function | BBM27 | 2 | 2 |
| lipoprotein |  | Cell envelope | BBP28 | 2 | 2 |
| plasmid partition protein, putative |  | Cellular processes | BBO32 | 2 | 2 |
| hypothetical protein |  |  | BBK47 | 2 | 2 |
| chemotaxis protein methyltransferase | cheR-1 | Cellular processes | BB0040 | 2 | 2 |
| hypothetical protein |  |  | BB0418 | 2 | 2 |
| hypothetical protein |  |  | BB0459 | 2 | 2 |
| conserved hypothetical protein |  | Hypothetical proteins | BB0782 | 2 | 2 |
