# Supplementary material for: Proteome Analysis of Borrelia burgdorferi Response to Environmental Change
Source: PLoS One. 2010 Nov 2;5(11):e13800. doi: 10.1371/journal.pone.0013800 (PMC2970547; doi:10.1371/journal.pone.0013800)
Supplement: Table S5 — Supplementary Table S5 (0.07 MB DOC) [file pone.0013800.s006.doc]

| chemotaxis protein methyltransferase | CheR methyltransferase, SAM binding domain (cheR) [2.1.1.80] {Borrelia burgdorferi B31} | BB0040 | cheR-1 | Cellular processes |
| --- | --- | --- | --- | --- |
| hypothetical protein | conserved hypothetical protein {Borrelia burgdorferi B31} | BB0165 |  |  |
| lipoprotein LA7 | outer surface 22 kda lipoprotein (antigen ipla7) {Borrelia burgdorferi B31} | BB0365 |  | Cell envelope |
| stage 0 sporulation protein J | stage 0 sporulation protein J (spo0J) {Borrelia burgdorferi B31} | BB0434 | spo0J | Cellular processes |
| Na+/H+ antiporter | Na+-H+ antiporter {Borrelia burgdorferi B31} | BB0447 | napA | Transport and binding proteins |
| hypothetical protein | tetratricopeptide repeat domain protein {Borrelia burgdorferi B31} | BB0459 |  |  |
| NH(3)-dependent NAD+ synthetase | NAD+ synthetase (nadE) [6.3.1.5] {Borrelia burgdorferi B31} | BB0522 |  | Biosynthesis of cofactors, prosthetic groups, and carriers |
| purine-binding chemotaxis protein | purine-binding chemotaxis protein {Borrelia burgdorferi B31} | BB0565 | cheW-2 | Cellular processes |
| ABC transporter, ATP-binding protein | ABC transporter, ATP-binding protein {Borrelia burgdorferi B31} | BB0573 |  | Transport and binding proteins |
| 1-phosphofructokinase | 1-phosphofructokinase (pfkB) [2.7.1.56] {Borrelia burgdorferi B31} | BB0630 | fruK | Energy metabolism |
| ferric uptake regulation protein | ferric uptake regulation protein {Borrelia burgdorferi B31} | BB0647 | fur | Regulatory functions |
| chemotaxis operon protein | CheC-like family protein {Borrelia burgdorferi B31} | BB0671 | cheX | Unknown function |
| hypothetical protein | lipoprotein, putative {Borrelia burgdorferi B31} | BB0689 |  |  |
| UDP-N-acetylmuramate--alanine ligase | UDP-N-acetylmuramate--alanine ligase (murC) [6.3.2.8] {Borrelia burgdorferi B31} | BB0817 | murC | Cell envelope |
| antigen, S2 | antigen, S2 {Borrelia burgdorferi B31} | BBA04 |  | Cell envelope |
| antigen, S1 | antigen, S1 {Borrelia burgdorferi B31} | BBA05 |  | Cell envelope |
| conserved hypothetical protein | conserved hypothetical protein {Borrelia burgdorferi B31} | BBA13 |  | Hypothetical proteins |
| decorin binding protein A | decorin-binding protein A {Borrelia burgdorferi B31} | BBA24 | dbpA | Cell envelope |
| decorin binding protein B | decorin-binding protein B {Borrelia burgdorferi B31} | BBA25 | dbpB | Cell envelope |
| oligopeptide ABC transporter, periplasmic oligopeptide-binding protein | oligopeptide ABC transporter, periplasmic oligopeptide-binding protein (oppA) {Borrelia burgdorferi B31} | BBA34 | oppAV | Transport and binding proteins |
| lipoprotein | lipoprotein {Borrelia burgdorferi B31} | BBA36 |  | Cell envelope |
| hypothetical protein | conserved hypothetical protein {Borrelia burgdorferi B31} | BBA38 |  |  |
| hypothetical protein | conserved hypothetical protein {Borrelia burgdorferi B31} | BBA39 |  |  |
| hypothetical protein | lyme disease proteins of unknown function {Borrelia burgdorferi B31} | BBA40 |  |  |
| conserved hypothetical protein | conserved hypothetical protein {Borrelia burgdorferi B31} | BBA41 |  | Hypothetical proteins |
| hypothetical protein | conserved hypothetical protein {Borrelia burgdorferi B31} | BBA48 |  |  |
| antigen, P35 | antigen, P35 {Borrelia burgdorferi B31} | BBA64 |  | Cell envelope |
| antigen, P35, putative | outer surface protein {Borrelia burgdorferi B31} | BBA66 |  | Cell envelope |
| antigen, P35, putative | antigen, P35, putative {Borrelia burgdorferi B31} | BBA73 |  | Cell envelope |
| outer surface protein C | outer surface protein C (PC) {Borrelia burgdorferi B31} | BBB19 | ospC | Cell envelope |
| hypothetical protein | lipoprotein, putative {Borrelia burgdorferi B31} | BBG01 |  |  |
| hypothetical protein | conserved hypothetical protein {Borrelia burgdorferi B31} | BBK47 |  |  |
| erpA protein | ErpA {Borrelia burgdorferi B31} | BBL39 | erpA | Cell envelope |
| erpB2 protein | ErpB2 {Borrelia burgdorferi B31} | BBL40 | erpB2 | Cell envelope |
| rev protein | rev protein {Borrelia burgdorferi B31} | BBM27 | rev | Unknown function |
| plasmid partition protein, putative | PF-32 protein {Borrelia burgdorferi B31} | BBO32 |  | Cellular processes |
| rev protein | Borrelia burgdorferi REV protein {Borrelia burgdorferi B31} | BBP27 | rev | Unknown function |
| lipoprotein | lipoprotein {Borrelia burgdorferi B31} | BBP28 |  | Cell envelope |
|  | lipoprotein, putative {Borrelia burgdorferi B31} | ORFZ01935 |  |  |
