# Supplementary material for: Proteome Analysis of Borrelia burgdorferi Response to Environmental Change
Source: PLoS One. 2010 Nov 2;5(11):e13800. doi: 10.1371/journal.pone.0013800 (PMC2970547; doi:10.1371/journal.pone.0013800)
Supplement: Table S6 — Supplementary Table S6 (0.07 MB DOC) [file pone.0013800.s007.doc]

| chemotaxis protein methyltransferase | Cellular processes | BB0040 | cheR-1 |
| --- | --- | --- | --- |
| leucyl-tRNA synthetase | Protein synthesis | BB0251 | leuS |
| cell division protein, putative | Cellular processes | BB0257 |  |
|  |  | BB0265 |  |
| oligopeptide ABC transporter, periplasmic oligopeptide-binding protein | Transport and binding proteins | BB0329 | oppA-2 |
| hypothetical protein |  | BB0418 |  |
| hypothetical protein |  | BB0509 |  |
| hypothetical protein |  | BB0555 |  |
| hypothetical protein |  | BB0563 |  |
| purine-binding chemotaxis protein | Cellular processes | BB0565 | cheW-2 |
| hypothetical protein |  | BB0566 |  |
| chemotaxis histidine kinase | Cellular processes | BB0567 | cheA-1 |
| hypothetical protein |  | BB0646 |  |
| purine-binding chemotaxis protein | Cellular processes | BB0670 | cheW-3 |
| chemotaxis operon protein | Unknown function | BB0671 | cheX |
| chemotaxis response regulator | Cellular processes | BB0672 | cheY-3 |
| methyl-accepting chemotaxis protein | Cellular processes | BB0680 | mcp-4 |
| methyl-accepting chemotaxis protein | Cellular processes | BB0681 | mcp-5 |
| hypothetical protein |  | BB0689 |  |
| NADH oxidase, water-forming | Energy metabolism | BB0728 | nox |
| glutamate transporter | Transport and binding proteins | BB0729 | gltP |
| RNA polymerase sigma factor | Transcription | BB0771 | rpoS |
| hypothetical protein |  | BB0776 |  |
| adenine phosphoribosyltransferase | Purines, pyrimidines, nucleosides, and nucleotides | BB0777 | apt |
| DNA mismatch repair protein | DNA metabolism | BB0797 | mutS |
| ornithine carbamoyltransferase, catabolic | Energy metabolism | BB0842 | arcB |
| antigen, S2 | Cell envelope | BBA04 |  |
| antigen, S1 | Cell envelope | BBA05 |  |
| chpAI protein, putative | Regulatory functions | BBA07 |  |
| decorin binding protein A | Cell envelope | BBA24 | dbpA |
| decorin binding protein B | Cell envelope | BBA25 | dbpB |
| hypothetical protein |  | BBA33 |  |
| oligopeptide ABC transporter, periplasmic oligopeptide-binding protein | Transport and binding proteins | BBA34 | oppAV |
| lipoprotein | Cell envelope | BBA36 |  |
| hypothetical protein |  | BBA37 |  |
| hypothetical protein |  | BBA57 |  |
| lipoprotein | Cell envelope | BBA62 |  |
| antigen, P35 | Cell envelope | BBA64 |  |
| hypothetical protein |  | BBA65 |  |
| antigen, P35, putative | Cell envelope | BBA66 |  |
| antigen, P35, putative | Cell envelope | BBA73 |  |
| hypothetical protein |  | BBB09 |  |
| hypothetical protein |  | BBH06 |  |
| antigen, P35, putative | Cell envelope | BBH32 |  |
|  |  | BBJ01 |  |
| hypothetical protein |  | BBJ28 |  |
| adenine deaminase | Purines, pyrimidines, nucleosides, and nucleotides | BBK17 | adeC |
| immunogenic protein P35 | Cell envelope | BBK32 |  |
| conserved hypothetical protein | Hypothetical proteins | BBL29 |  |
| conserved hypothetical protein | Hypothetical proteins | BBM35 |  |
| hypothetical protein |  | BBO29 |  |
| hypothetical protein |  | BBO43 |  |
| conserved hypothetical protein | Hypothetical proteins | BBP29 |  |
| conserved hypothetical protein | Hypothetical proteins | BBR36 |  |
| conserved hypothetical protein | Hypothetical proteins | BBS31 |  |
|  |  |  |  |
|  |  |  |  |
